# Cyclization–endoperoxidation cascade reactions of dienes mediated by a pyrylium photoredox catalyst

**DOI:** 10.3762/bjoc.10.128

**Published:** 2014-06-03

**Authors:** Nathan J Gesmundo, David A Nicewicz

**Affiliations:** 1 Department of Chemistry, University of North Carolina at Chapel Hill, Chapel Hill, NC 27599-3290, USA

**Keywords:** alkene, cascade, endoperoxide, oxidation, photoredox catalysis

## Abstract

Triarylpyrylium salts were employed as single electron photooxidants to catalyze a cyclization–endoperoxidation cascade of dienes. The transformation is presumed to proceed via the intermediacy of diene cation radicals. The nature of the diene component was investigated in this context to determine the structural requirements necessary for successful reactivity. Several unique endoperoxide structures were synthesized in yields up to 79%.

## Introduction

**Figure 1:**
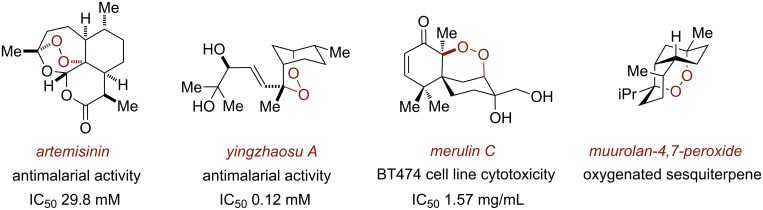
Selected examples of endoperoxide-containing natural products.

Endoperoxides are a structurally unique class of naturally-occurring compounds that feature a reactive cyclic peroxide moiety of varying ring sizes (Figure 1). The lability of the endocyclic peroxide O–O bond engenders these compounds with a range of important biological functions, most notably, antimalarial and antitumor activity (e.g., artemisinin, yingzhaosu A and merulin C) [1–4]. From a synthetic standpoint, the installation of the endoperoxide moiety presents a significant challenge due to its susceptibility to reduction and for this reason, is ideally introduced late-stage in target-oriented synthesis. Additionally, many endoperoxide natural products possess architecturally complex frameworks (e.g., artemisinin, yingzhaosu A, muurolan-4,7-peroxide) [5] that pose significant synthetic challenges.

Classical approaches to the introduction of cyclic peroxides typically rely on cycloadditions of alkenes and dienes with singlet oxygen. However, ene processes can often compete, leading to complex mixtures of hydroperoxide adducts [1,6–8]. More recently, cyclization reactions of hydroperoxides with pendant alkenes or alkynes have been developed. Selected examples include Pd(II)-catalyzed hydroalkoxylation reactions of unsaturated hydroperoxides [9], Au(I)-catalyzed endoperoxidation of alkynes [10] and Brønsted acid-catalyzed enantioselective acetalization/oxa-Michael addition cascade reactions of peroxyquinols [11].

**Scheme 1:**
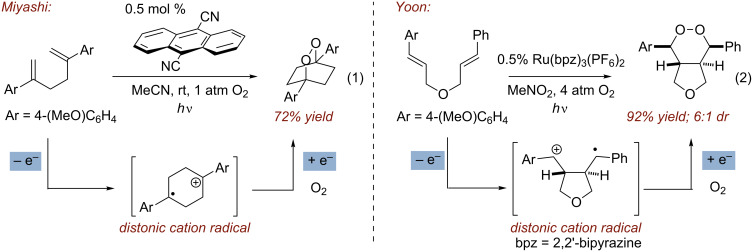
Endoperoxide formation via cation radicals. In both examples, single electron oxidation is followed quickly by cyclization to form stabilized distonic cation radical intermediates. The distonic intermediates are trapped by O_2_ and furnish the shown bicyclic products after reduction.

While these extant methods are effective at installing the endoperoxide functional group, our interest lay in developing strategies that simultaneously forged both the cyclic peroxide as well as the carbon framework to rapidly build molecular complexity. For this reason, we were inspired by the work of Miyashi, who, during the course of the investigation of the cation radical Cope rearrangement, discovered an intriguing endoperoxide-forming reaction (Scheme 1, reaction 1). Upon exposure of 1,5 and 1,6-dienes to catalytic quantities of 9,10-dicyanoanthracene (DCA) under UV irradiation in the presence of oxygen, bicyclic endoperoxides were obtained [12]. Formation of the 1,2-dioxanes was presumed to occur via single electron oxidation of the diene by the excited state DCA followed by either 6-*endo* or 7-*endo* cyclization modes to generate a fleeting distonic cation radical species. Interception of the distonic cation radical by triplet oxygen and back electron transfer completes the catalytic cycle. While later reports expanded the scope of this transformation modestly [13–14], we felt that this strategy had potential to be a more general method. Indeed, during the course of this work, the Yoon research group disclosed a similar strategy employing Ru(bpz)_3_^2+^ as the photooxidant to effect a 5-*exo* cyclization/endoperoxidation cascade of bis(styrene) substrates (Scheme 1, reaction 2) [15]. More recently, Kamata and Kim have employed this reaction manifold to forge endoperoxides from 1,2-divinylarene precursors [16].

**Scheme 2:**
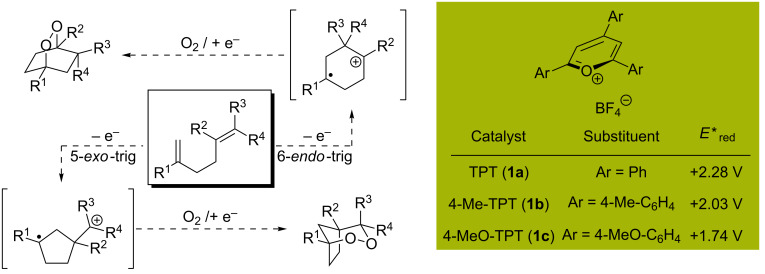
Diversification strategy for endoperoxide synthesis by single electron transfer. E*_red_ vs SCE [20].

We envisioned that the scope of this transformation could be extended to include non-styrenal dienes as well as alternative cyclization modes, provided that a potent single electron oxidant could be identified (*E*_red_ > +1.5 V vs SCE). Given the paucity of ground-state single electron oxidants capable of this task, we elected to employ photooxidation catalysts. Additionally, we sought to select visible light-activated organic single electron oxidants that do not readily sensitize singlet oxygen [17–19]. For these reasons, we were attracted to the use of triarylpyrylium salts, as they have excited state reduction potentials in excess of +1.7 V vs SCE (Scheme 2) [20]. In addition, prior work demonstrates that these catalysts are productive in cation radical mediated [4 + 2], [2 + 2], oxygenation, and rearrangement chemistry [21–22]. We also sought to delineate the reactivity of the diene with respect to its structure to better predict the mode of diene cation radical cyclization (5-*exo* vs 6-*endo*). Herein is reported an organocatalytic photoredox-mediated strategy for the endoperoxidation of 1,5-dienes using ^3^O_2_ to rapidly generate complex endoperoxide frameworks.

## Results and Discussion

### Reaction optimization

**Figure 2:**
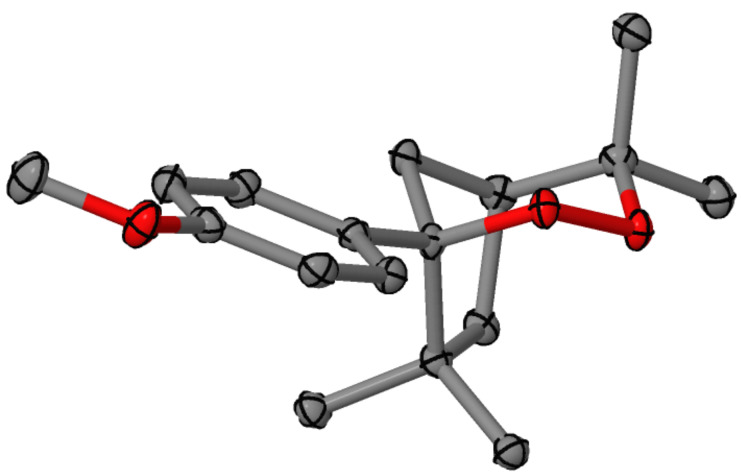
ORTEP of **3a**.

We began our investigation into endoperoxidation conditions with diene **2a** as the substrate as it contained both a styene and an aliphatic alkene. Using catalyst **1c** in DCM at −41 °C under irradiation with 470 nm LEDs afforded endoperoxide **3a** in 40% yield after 5 hours (Table 1, entry 1). The observed endoperoxide was attributed to a 5-*exo* cyclization mode of the diene cation radical followed by capture of molecular oxygen. The use of acetonitrile as solvent gave none of the desired adducts (Table 1, entry 2). Further improvement of the chemical yield of **3a** was realized by increasing the reaction concentration (Table 1, entries 3–5), resulting in a 70% yield (^1^H NMR) of the endoperoxide (Table 1, entry 5). In all reactions, substrate conversion was 100%, with the remainder of the mass balance in all cases representing oxidative decomposition pathways. When O_2_ was excluded from the reaction (Table 1, entry 6), none of the endoperoxide was observed. Not surprisingly, exclusion of light or catalyst **1c** from the reaction (Table, 1 entries 7 and 8 respectively) resulted in no product formation. Interestingly, when 9-mesityl-10-methylacridinium tetrafluoroborate, which has been used with success in other photooxidation processes [23–26], was used in place of **1c**, complete consumption of **2a** was observed, but **3a** was not produced. Once again only oxidative decomposition pathways seemed active, this time possibly due to superoxide formation. Lastly, to exclude the intervention of a singlet oxygen mechanism, we conducted the reaction in the presence of Rose Bengal. Under these conditions, we observed only ^1^O_2_ ene reactivity with the isoprenyl group (65% yield of hydroperoxide), underscoring the unique reactivity garnered by this catalyst system (see Supporting Information File 1 for hydroperoxide characterization). Suitable crystals of **3a** provided X-ray confirmation of the endoperoxide structure (Figure 2).

**Table 1:** Reaction Optimization and Control Experiments.

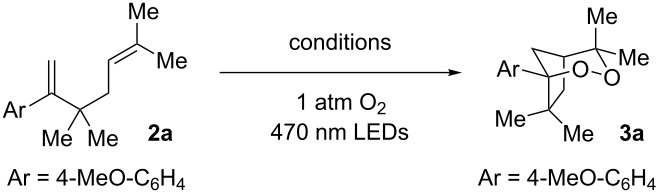			

Entry	Conditions^a^	Conversion	Yield^b^

1	2 mol % **1c** 0.01 M DCM, −41 °C	100%	40%
2	2 mol % **1c** 0.01 M MeCN, −41 °C	100%	0%
3	2 mol % **1c** 0.02 M DCM, −41 °C	100%	63%
4	1 mol % **1c** 0.05 M DCM, −41 °C	100%	63%
5	0.7 mol % **1c** 0.07 M DCM, −41 °C	100%	70%
6^c^	Excluding O_2_	11%	0%
7	Excluding *h*ν	0%	0%
8	Excluding catalyst **1c**	0%	0%
9	9-Mes-10-Me-Acr-BF_4_ in place of **1c**	100%	0%
10^d^	Rose Bengal in place of **1c**	100%	0%

All reactions carried out in oxygen-saturated solvents unless otherwise noted. ^a^−41 °C found to be the optimum temperature during initial substrate/reaction optimization. ^b^Yields with respect to (Me_3_Si)_2_O ^1^H NMR internal standard. ^c^Reaction carried out under N_2_ atmosphere in DCM. ^d^Reaction carried out in oxygen saturated MeOH at room temperature using a white flood lamp.

**Scheme 3:**
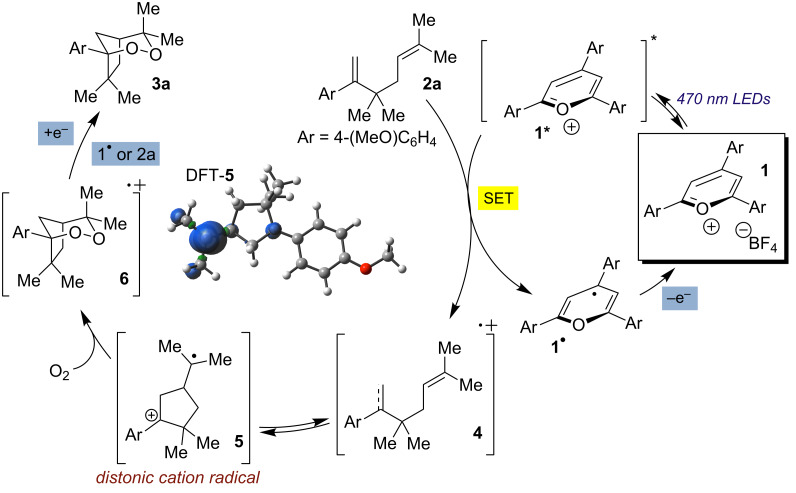
Proposed mechanism for endoperoxide synthesis from tethered dienes.

We invoke a mechanism similar to that proposed by Miyashi [12] and Yoon [15] in their respective transformations (Scheme 3). Following excitation of triarylpyrylium tetrafluoroborate catalyst **1**, one-electron oxidation of the 1,5-diene substrate produces localized cation radical intermediate **4** and pyranyl radical **1^•^**. Cyclization of diene cation radical **4** then forms stabilized distonic cation radical intermediate **5**, which is intercepted by O_2_ to form **6**. Single electron reduction, either from **1^•^** to regenerate active catalyst **1** or from another substrate equivalent in a chain process, forms the desired bicyclic endoperoxide.

DFT calculations suggest that the formation of the initial distonic cation radical **5** is exothermic by approximately 3 kcal/mol relative to cation radical **4** [27]. Superficially, this is rationalized by the increased substitution on **5** relative to **4**. In addition, the majority of the spin density on **5** is located on the isoprenyl group (DFT-**5**, Scheme 3). This may be fortuitous as stereoselectivity in the oxygen addition step is irrelevant, whereas the opposite scenario involving a benzylic radical intermediate would require a stereospecific addition of oxygen to the same face of the cyclopentane system as the isoprenyl cation in order for endoperoxide formation to occur.

With optimized conditions identified, we sought to examine the scope of the reaction with respect to the diene structure. Miyashi’s 1,5-diene (**2b**; *E*_1/2_^Ox^ = 1.22 V vs SCE [12]) afforded a 50% yield of the expected endoperoxide along with ~5% of a 1,4-dione, presumably from double oxidative cleavage of the 1,5-diene (Table 2, entry 1). Unfortunately, attempts to move away from **2b** to less electron-rich dienes such as **2c**, **2d** and **2e** (Table 1, entries 2–4), failed to produce any of the desired endoperoxide products and mainly oxidative cleavage adducts were observed.

**Table 2:** Diene structure investigation for endoperoxidation cascade.

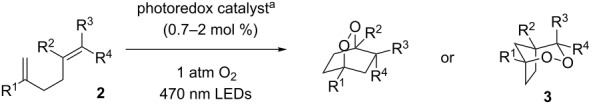			

Entry	Substrate	Expected adduct	Yield^b^

1	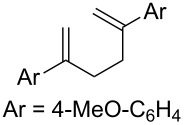 **2b**	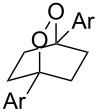 **3b**	50%
2	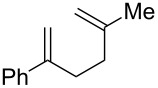 **2c**	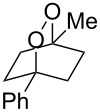 **3c**	0%
3	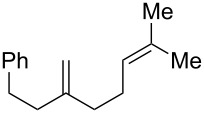 **2d**	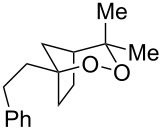 **3d**	0%
4	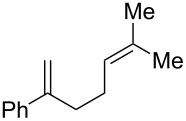 **2e**	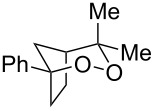 **3e**	0%
5	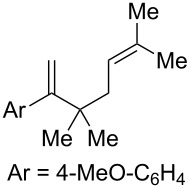 **2a**	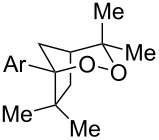 **3a**	66%
6	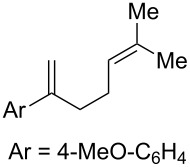 **2f**	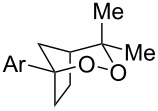 **3f**	32%
7	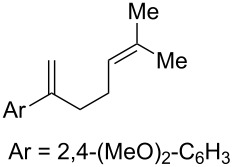 **2g**	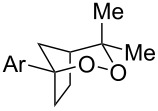 **3g**	9%
8	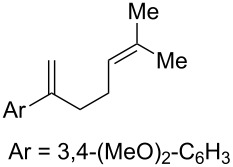 **2h**	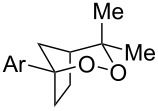 **3h**	0%
9	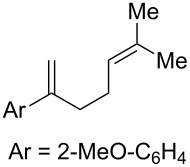 **2i**	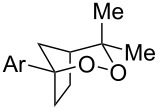 **3i**	16%
10	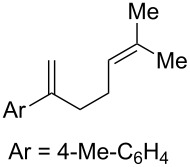 **2j**	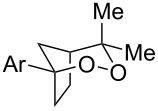 **3j**	0%
11	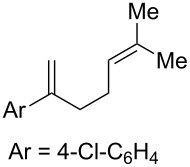 **2k**	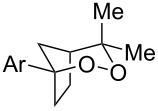 **3k**	0%
12	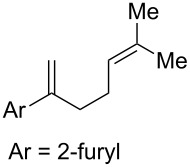 **2l**	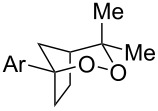 **3l**	0%

All reactions carried out in oxygen-saturated solvents. Solvents examined: DCM, CHCl_3_, MeCN, PhMe, acetone. ^a^Catalysts **1a**, **1b**, and **1c** screened for reactivity with all substrates. ^b^Average of two isolated yields.

Given the lack of reactivity of this alkene substitution pattern, we turned our attention to the investigation of diene structures similar to successful endoperoxidation substrate **2a**. Removal of the geminal dimethyl group (**2f**) resulted in significantly diminished yields of the endoperoxide adduct, likely due to the lack of a Thorpe–Ingold effect present in **2a**. These experiments also demonstrated that the electron-rich arene was necessary for reactivity (cf. **2e** and **2f**; Table 2, entries 4 and 6). The importance of the electron-rich arene may lie in the necessary distonic cation radical intermediate: the electron-rich arene may provide greater stability to the distonic intermediate formed after 5-*exo*-trig cyclization, ultimately ensuring it remains to intercept oxygen. A further survey of the styrene component supported this hypothesis. While electron-rich styrenes gave modest amounts of product formation (**2f**, **2g** and **2i**; Table 2, entries 6, 7 and 9), styrenes with either weakly donating (4-Me; Table 2, entry 10) or even withdrawing (4-Cl; Table 2, entry 11) functionality furnished none of the expected endoperdoxides. In these cases, oxidative degradation was observed as was the case with the highly oxidizable 2-furyl group (Table 2, entry 12). Interestingly, 3,4-dimethoxystyrene-substituted diene **2h** also gave none of the desired adduct, which we attributed to lack of charge density on the alkene [28–29].

**Scheme 4:**
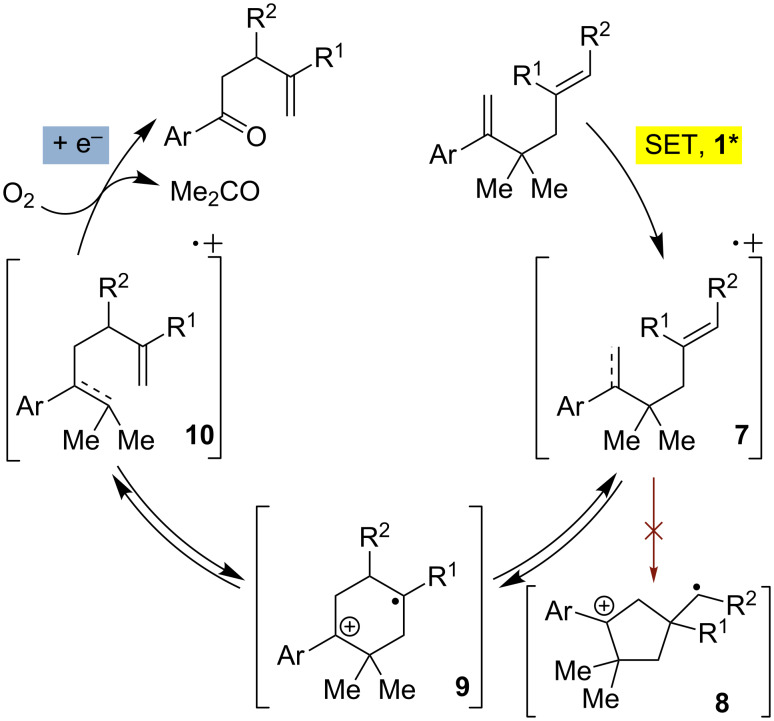
Competing formal [3,3] pathway.

We next investigated the endoperoxidation cascade by replacing the isoprenyl substituent with a variety of other alkenes. A pendant styrene afforded the desired endoperoxide adduct in 68% yield, albeit with no diastereocontrol (**2m**, 1:1 dr; Table 3, entry 1). A diene bearing a tetrasubstituted alkene (**2n**) was reactive in this context, giving polycyclic endoperoxide **3n** in 64% yield (6.5:1 dr). The use of 1,2-, 1,1-dialkyl as well as monoalkyl-substituted alkenes appeared to completely disfavor the endoperoxidation pathway and resulted in the unexpected isolation of α-allyl ketones (Table 3, entries 3–5) in modest yields. Based on the Miyashi precedent, the formation of these adducts can be rationalized by invoking a formal [3,3]-rearrangement of the initial cation radical intermediate. The competing 6-*endo* cyclization mode and formation of distonic cation radical **9** ultimately provides access to the more stabilized cation radical **10**, which undergoes oxidative cleavage to afford the corresponding α-allyl ketones (Scheme 4). In the absence of the geminal dimethyl group (**2r**, **2s**), neither the Cope-like reactivity or the endoperoxidation was observed (Table 3, entries 6 and 7).

**Table 3:** Variation of the tethered alkene component.

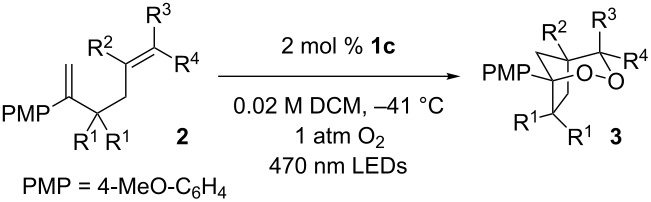			

Entry	Substrate	Observed product	Yield^a^

1^b^	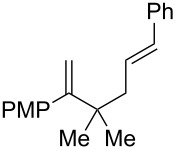 **2m**	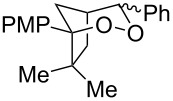 **3m**	68%, 1:1 d.r.
2^c^	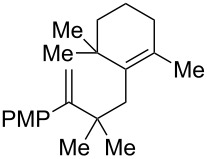 **2n**	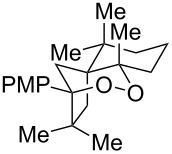 **3n**	64%, 6.5:1 d.r.
3^d^	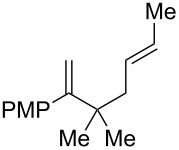 **2o**	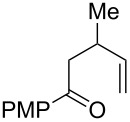 **3o**	36%
4^d^	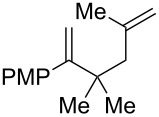 **2p**	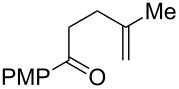 **3p**	37%
5^d^	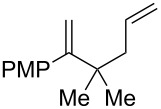 **2q**	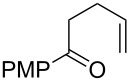 **3q**	27%
6^e^	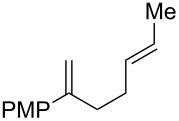 **2r**	–	0%
7^e^	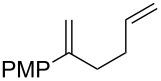 **2s**	–	0%

Reactions carried out in oxygen-saturated solvents. ^a^Average of two isolated yields. ^b^1:1 mixture of separable diastereomers. ^c^6.5:1 mixture of inseparable diastereomers. Presumed major diastereomer shown. ^d^Desired endoperoxide never observed. ^e^Multiple conditions tested, no productive chemistry observed.

Lastly, we elected to explore cyclization modes similar to the Yoon work under the developed conditions. Diene **2t** was anticipated to undergo 6-*exo*-trig cyclization to form the necessary distonic cation radical intermediate (Table 4, entry 1). The expected trioxabicyclo[3.3.1]nonane product was formed, albeit in low yields (16%), where the remainder of the mass balance was attributed to oxidative degradation.

**Table 4:** Other cyclization modes and substrate designs.

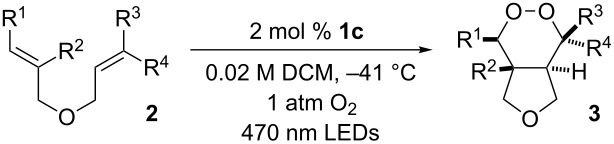			

Entry	Substrate	Desired product	Yield^a^

1^b^	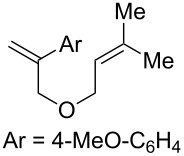 **2t**	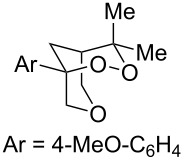 **3t**	16%
2	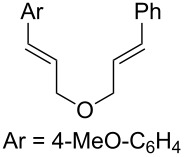 **2u**	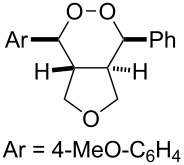 **3u**	79%, 5.7:1 d.r.
3	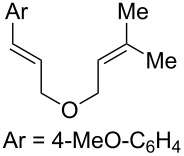 **2v**	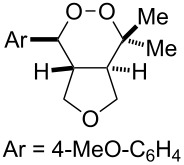 **3v**	<5%
4^c^	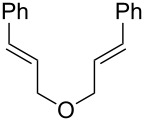 **2w**	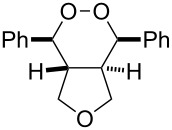	0%

Reactions carried out in oxygen-saturated dichloromethane. ^a^Average of two isolated yields. ^b^Carried out in 0.4 M DCE after solvent and concentration optimization. ^c^Catalysts **1a** or **1b** were also tested but failed to furnish the endoperoxide.

Bis(styrene) **2u** afforded the identical fused 1,2-dioxane observed in Yoon’s report in 79% yield (5.7:1 dr). Tethered trisubstituted aliphatic alkene substrate **2v**, along with bis(styrene) substrate **2w** were unfortunately unsuccessful, producing neither of the desired fused 1,2-dioxane products in appreciable amounts. Degradation pathways were dominant for **2v** and mainly unreacted starting material was observed **2w**.

## Conclusion

In the presence of an organic single electron photooxidant, a variety of dienes were demonstrated to undergo a cyclization/endoperoxidation cascade sequence to form 1,2-dioxanes. Requirements for successful diene reactivity are the presence of an oxidizable olefin and an alkene that can efficiently react with the putative alkene cation radical to form a more stable distonic cation radical. If available, a Cope-like pathway can compete and suppress endoperoxide formation. With these parameters in mind, this reaction could provide a platform for the discovery of novel biologically-active endoperoxides.

## Supporting Information

File 1 Experimental procedures and characterization data.

File 2 X-ray data.
