# Supplementary material for: Cyclization–endoperoxidation cascade reactions of dienes mediated by a pyrylium photoredox catalyst
Source: Beilstein J Org Chem. 2014 Jun 3;10:1272–81. doi: 10.3762/bjoc.10.128 (PMC4077508; doi:10.3762/bjoc.10.128)
Supplement: File 1 — Experimental procedures and characterization data. [file Beilstein_J_Org_Chem-10-1272-s001.pdf]

Supporting Information File 1

for

Cyclization–endoperoxidation cascade reactions of dienes mediated by a pyrylium photoredox catalyst

Nathan J. Gesmundo and David A. Nicewicz*

Address: Department of Chemistry, University of North Carolina at Chapel Hill, Chapel Hill, NC 27599-3290, USA

Email: David A. Nicewicz - nicewicz@unc.edu

*Corresponding author

Experimental procedures and characterization data

Table of Contents

I. General Information S2

II. Optimization of conditions for substrate **2f** S3

III. Thermogravimetric analysis for **3a** S4

IV. Preparation of 2,4,6-triarylpyrylium tetrafluoroborate photosensitizers S4

V. Preparation of unconjugated diene substrates S5–S14

VI. General procedures for PET endoperoxidation S15

3a–**3u** S15–S19

VII. Hydroperoxide via $^1O_2$ S19

VIII. ^{1}H and ^{13}C NMR spectra S20–S91

IX. References S91

I. General information. Proton and carbon nuclear magnetic resonance spectra (^{1}H NMR and ^{13}C NMR) were recorded on a Bruker model DRX 400 or AVANCE III 600 CryoProbe (^{1}H NMR at 400 MHz or 600 MHz and ^{13}C NMR at 100 MHz or 150 MHz) spectrometer. Chemical shifts for protons are reported in parts per million downfield from tetramethylsilane and are referenced to residual protium in solvent (^{1}H NMR: $CHCl_3$ at 7.24 ppm). Chemical shifts for carbons are reported in parts per million downfield from tetramethylsilane and are referenced to the carbon resonances of the residual solvent peak (^{13}C NMR: $CDCl_3$ at 77.0 ppm). NMR data are represented as follows: chemical shift, multiplicity (s = singlet, bs = broad singlet, d = doublet, dd = doublet of doublet, t = triplet, ddd = doublet of doublet of doublet, q = quartet, m = multiplet), coupling constants (Hz), and integration. Mass spectra were obtained using a Micromass Quattro II (triple quad) instrument with nanoelectrospray ionization. Analytical thin layer chromatography (TLC) was performed on SiliaPlate 250 µm thick silica gel plates provided by Silicycle. Visualization was accomplished using fluorescence quenching, $KMnO_4$ stain, or ceric ammonium molybdate (CAM) stain followed by heating. Organic solutions were concentrated under reduced pressure using a Büchi rotary evaporator. Purification of the reaction products was carried out by chromatography using Siliaflash-P60 (40–63 µm) or Siliaflash-T60 (5–20 µm) silica gel purchased from Silicycle. All reactions were carried out under an inert atmosphere of nitrogen in flame-dried glassware with magnetic stirring or under an atmosphere of oxygen in flame-dried glassware with magnetic stirring unless otherwise noted. Irradiation of photochemical reactions was carried out using a SE-WFLS-B60 blue (470 nm) LED flexible light strip purchased from Super Bright LEDs Inc. (St. Louis, MO, USA), with standard borosilicate glass vials purchased from Fisher Scientific. Yield refers to isolated yield of analytically pure material unless otherwise noted. NMR yields were determined using hexamethyldisiloxane, $(Me_3Si)_2O$, as an internal standard.

Procedure for yield determination using $(Me_3Si)_2O$ NMR internal standard: upon consumption of starting material (as determined by TLC), crude reaction mixture was passed through a short silica gel plug with DCM. The crude mixture was concentrated and then taken up in deuterated NMR solvent ($CDCl_3$). A carefully measured amount of $(Me_3Si)_2O$ was then added to the solution via micro syringe and a ^{1}H NMR spectrum acquired.

Materials. Commercially available reagents were purchased from Sigma–Aldrich, Acros, Alfa Aesar, or TCI, and used as received unless otherwise noted. Diethyl ether (Et_2O), dichloromethane (DCM), tetrahydrofuran (THF), toluene (PhMe), and dimethylformamide (DMF) were dried by passing through activated alumina columns under nitrogen prior to use. Other common solvents and chemical reagents were purified by standard published methods where noted.

II. Optimization of conditions for substrate 2f

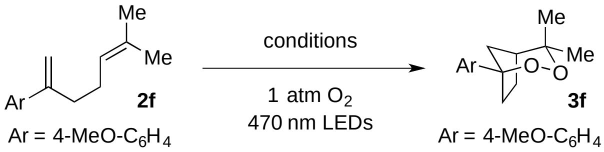

Entry	Conditions	Conversion	Yield[a]
1	2 mol% **1c**<br>0.01 M DCM, -41 °C	100%	22%
2	2 mol% **1c**<br>0.01 M DCM, -78 °C	100%	10%
3[b]	2 mol% **1c**<br>0.01 M DCM, -41 °C, 1 atm Air	100%	20%
4	2 mol% **1c**<br>0.02 M DCM, -41 °C	100%	16%
5	2 mol% **1c**<br>0.02 M DCM, -10 °C	100%	20%
6	2 mol% **1c**<br>0.02 M $CHCl_3$, -10 °C	100%	22%
7	2 mol% **1c**<br>0.02 M DCE, -10 °C	100%	30%
8	2 mol% **1c**<br>0.02 M DCE, rt	100%	28%

All reactions carried out in oxygen saturated solvents unless otherwise noted. [a]Yields reported with respect to $(Me_3Si)_2O$ ^1H NMR internal standard. [b]Reaction carried out under balloon of air, in air saturdated DCM.

III. Thermogravimetric analysis for product 3a

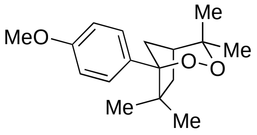

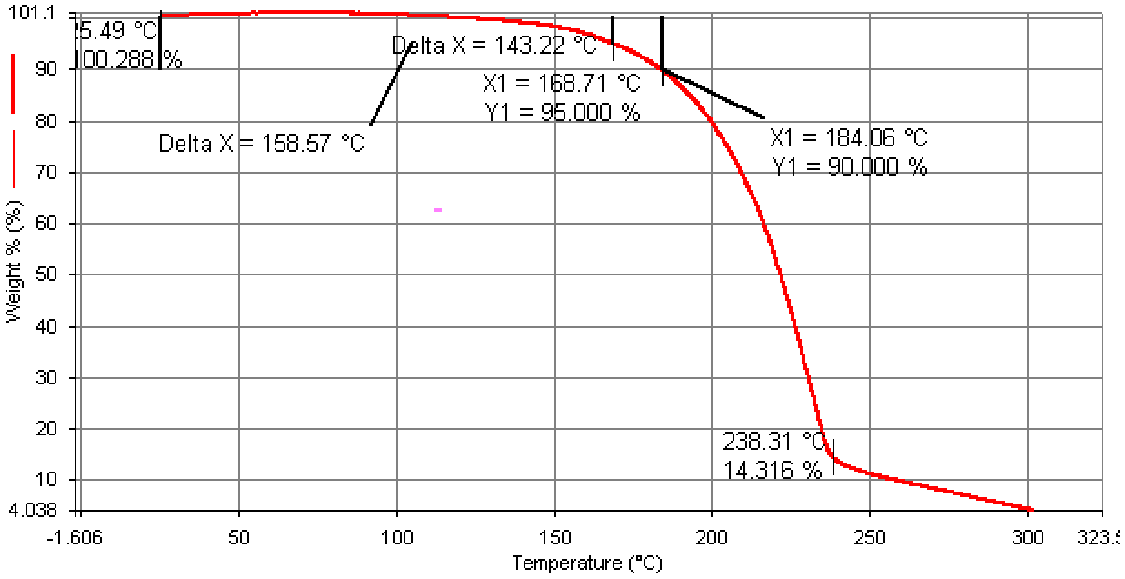

IV. Preparation of 2,4,6-triarylpyrylium tetrafluoroborate photosensitizers

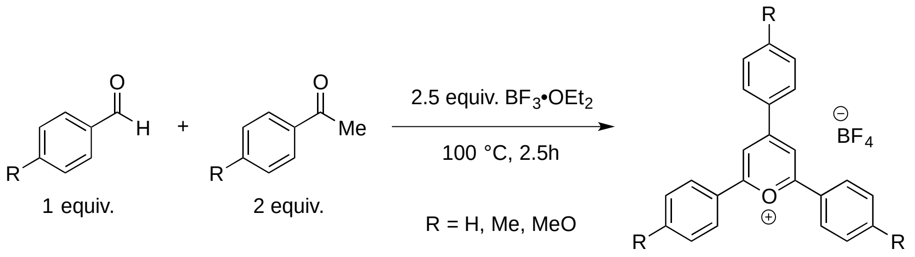

All variants prepared according to a published procedure; spectral data were in agreement with the literature values.[1]

V. Preparation of unconjugated diene substrates

4,4'-(Hexa-1,5-diene-2,5-diyl)bis(methoxybenzene) (2b)

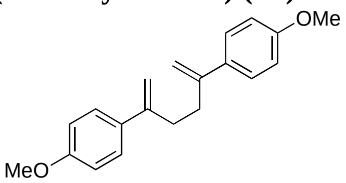

Prepared according to a published procedure; spectral data were in agreement with the literature values.[2]

(5-Methylhexa-1,5-dien-2-yl)benzene (2c)

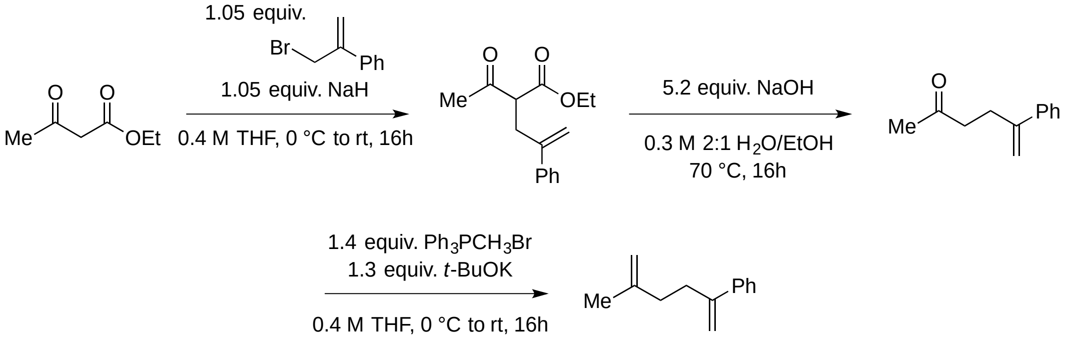

1.05 equiv of sodium hydride were dispensed into a flame-dried round bottom flask, which was subsequently purged with N_2. The sodium hydride was suspended in dry THF (0.4 mmol/mL) and the stirred suspension was cooled to 0 °C in an ice bath. At 0 °C, ethyl acetoacetate was added dropwise with stirring. The solution was stirred at 0 °C for 30 minutes after effervescence ceased. At 0 °C, (3-bromoprop-1-en-2-yl)benzene (1.05 equiv) was added to the mixture dropwise. The reaction was allowed to warm to room temperature and stirred for 16 hours overnight. The reaction was then quenched with concentrated NH_4Cl solution and diluted with DI water and diethyl ether. The organic layer was separated and aqueous layer extracted three times with diethyl ether in a separatory funnel. The combined organics were washed with brine, dried with anhydrous $MgSO_4$, filtered, and concentrated to afford a crude oil, which was taken on to the next step without further purification.

Decarboxylation of the crude alkylation product was carried out as follows: sodium hydroxide (5.2 equiv) was weighed into a round bottom flask containing a 2:1 water/ethanol mixture (approx. concentration 0.3 mmol/mL) and stirred to dissolution. At room temperature the crude alkylation product was introduced dropwise, then the reaction was heated to 70 °C overnight. The reaction was cooled to room temperature and quenched with concentrated NH_4Cl. The organic phase was separated and the aqueous phase extracted three times with diethyl ether. The combined organics were washed with brine, dried with anhydrous $MgSO_4$, filtered, and concentrated to afford a crude oil, which was taken on to the next step without further purification.

The crude aryl ketone was converted to the diene product by Wittig olefination. Olefination procedure for this substrate was identical to the Wittig olefination procedure in **General procedure A** below (step 2). The final product was purified using silica gel column chromatography (20% EtOAc/hexanes) to furnish 0.78 g of pure product (47% overall) as a colorless oil. Spectral data were in agreement with the reported literature values.[3] Analytical data

for **2c**: **^{1}H NMR** (600 MHz, $CDCl_3$): δ 7.40 (m, 2H), 7.32 (m, 2H), 7.26 (m, 1H), 5.28 (d, *J* = 1.2 Hz, 1H), 5.08 (d, *J* = 1.2 Hz, 1H), 4.73 (d, *J* = 1.2 Hz, 1H), 4.68 (d, *J* = 1.2 Hz, 1H), 2.64 (dt, *J* = 7.8 Hz, *J* = 1.2 Hz, 2H), 2.16 (t, *J* = 7.8 Hz, 2H), 1.74 (s, 3H); **^{13}C NMR** (150 MHz, $CDCl_3$); δ 148.2, 145.4, 141.2, 128.3, 127.3, 126.1, 112.3, 110.0, 36.5, 33.6, 22.5

(6-Methylhepta-1,5-dien-2-yl)benzene (2e)

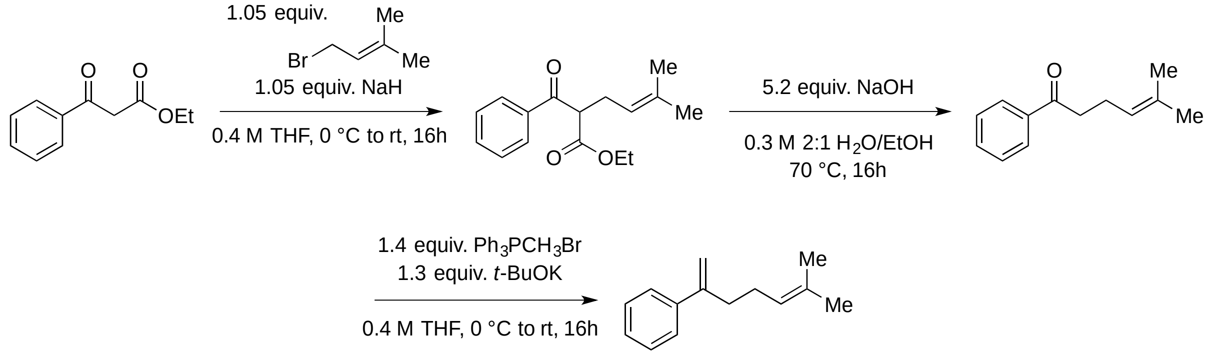

Carried out using the procedure above for synthesis of **2c**. In the initial alkylation, ethyl benzoyl acetate was used in place of ethyl acetoacetate and 3,3-dimethylallyl bromide in place of (3-bromoprop-1-en-2-yl)benzene. The remaining steps were carried out without alteration. The final product was purified using silica gel column chromatography (20% Et_2O/hexanes) to furnish 0.67 g of pure product (50% overall) as a colorless oil. Spectral data were in agreement with the reported literature values.[3] Analytical data for **2e**: **^{1}H NMR** (600 MHz, $CDCl_3$): δ 7.41 (m, 2H), 7.32 (m, 2H), 7.26 (m, 1H), 5.27 (s, 1H), 5.14 (m, 1H), 5.06 (s, 1H), 2.51 (t, *J* = 7.2 Hz, 2H), 2.13 (q, *J* = 7.2 Hz, 2H), 1.67 (s, 3H), 1.54 (s, 3H); **^{13}C NMR** (150 MHz, $CDCl_3$); δ 148.3, 141.3, 131.9, 128.2, 127.2, 126.1, 123.8, 112.2, 35.4, 26.9, 25.7, 17.7

General procedure A: Preparation of substrates with geminal dimethyl tether

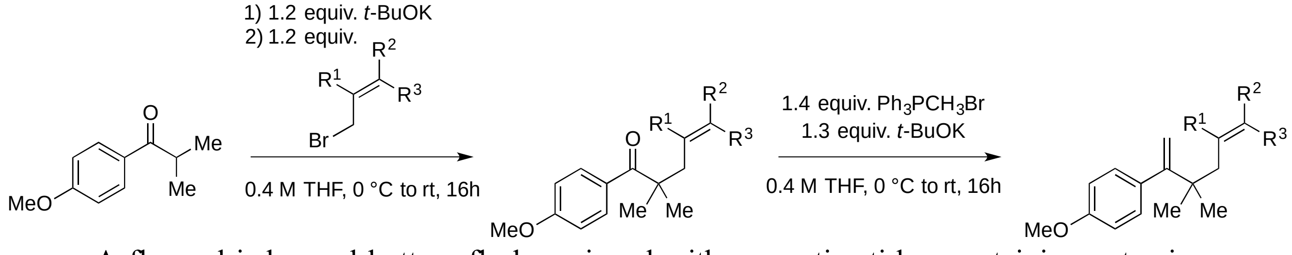

A flame-dried round bottom flask equipped with magnetic stirbar containing potassium *tert*-butoxide (1.2 equiv) was purged with nitrogen. Dry THF was added via syringe (approximate concentration 0.4 mmol/mL) and the solution was cooled to 0 °C in an ice bath with stirring. At 0 °C, 1-(4-methoxyphenyl)-2-methylpropan-1-one (prepared according to literature procedure[4]) was added to the stirring solution via syringe dropwise and stirred for 1 hour. At 0 °C, the appropriate allylic bromide was added dropwise via syringe and stirred at room temperature for 16 hours. The reaction was then quenched with concentrated NH_4Cl solution and diluted with DI water and diethyl ether. The organic layer was separated and aqueous layer extracted three times with diethyl ether in a separatory funnel. The combined organics were washed with brine, dried with anhydrous $MgSO_4$, filtered, and concentrated to afford a crude oil, which was taken on to the next step without further purification (acceptable purity confirmed by TLC and ^{1}H NMR).

The crude allylation product was converted to the desired diene by Wittig olefination. Potassium *tert*-butoxide (1.3 equiv) and methyltriphenylphosphonium bromide (1.4 equiv) were dispensed into a flame-dried round bottom flask equipped with a magnetic stirbar. The flask was purged with nitrogen and cooled to 0 °C with stirring. At 0 °C, dry THF was added via syringe (approximate concentration 0.4 mmol/mL) and the solution was stirred at 0 °C for 1 hour. At 0 °C, the crude allylation product was added via syringe (in THF) dropwise. The reaction was warmed to room temperature and stirred overnight. Upon completion, the reaction was quenched with concentrated NH_4Cl solution and then diluted with DI water and diethyl ether. The organic layer was separated and aqueous layer extracted three times with diethyl ether in a separatory funnel. The combined organics were washed with brine, dried with anhydrous $MgSO_4$, filtered, and concentrated to afford the crude product. The product was purified by silica gel column chromatography.

1-Methoxy-4-(3,3,6-trimethylhepta-1,5-dien-2-yl)benzene (2a)

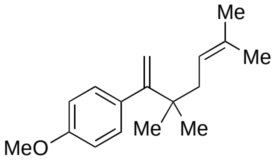

Diene prepared according to **general procedure A** using 3,3-dimethylallyl bromide as the electrophile in the first step. The final product was purified by silica gel column chromatography (20% diethyl ether/hexanes) to furnish 2.16 g of pure product (88%) as a colorless oil. Analytical data for **2a**: **^{1}H NMR** (600 MHz, $CDCl_3$): δ 7.05 (d, *J* = 9.0 Hz, 2H), 6.80 (d, *J* = 9.0 Hz, 2H), 5.18 (m, 1H), 5.11 (s, 1H), 4.83 (s, 1H), 3.79 (s, 3H), 2.03 (d, *J* = 7.2 Hz, 2H), 1.73 (s, 3H), 1.56 (s, 3H), 1.06 (s, 6H); **^{13}C NMR** (150 MHz, $CDCl_3$); δ 158.0, 157.6, 135.8, 132.6, 129.9, 121.5, 113.3, 112.7, 55.1, 39.7, 39.0, 27.4, 26.0, 18.1

(*E*)-1-(3,3-Dimethyl-6-phenylhexa-1,5-dien-2-yl)-4-methoxybenzene (2m)

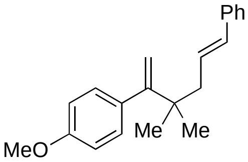

Diene prepared according to **general procedure A** using cinnamyl bromide as the electrophile in the first step. The final product was purified by silica gel column chromatography (20% diethyl ether/hexanes) to furnish 2.73 g of pure product (93%) as a yellow oil. Analytical data for **2m**: **^{1}H NMR** (600 MHz, $CDCl_3$): δ 7.36 (d, *J* = 7.8 Hz, 2H), 7.31 (t, *J* = 7.2 Hz, 2H), 7.21 (t, *J* = 7.8 Hz, 1H), 7.12 (d, *J* = 8.4 Hz, 2H), 6.85 (d, *J* = 8.4 Hz, 2H), 6.37 (d, *J* = 15.6 Hz, 1H), 6.23 (m, 1H), 5.18 (s, 1H), 4.90 (s, 1H), 3.82 (s, 3H), 2.29 (d, *J* = 6.6 Hz), 1.15 (s, 6H); **^{13}C NMR** (150 MHz, $CDCl_3$); δ 158.2, 157.3, 137.8, 135.6, 132.0, 130.0, 128.5, 127.8, 126.9, 126.0, 113.7, 112.8, 55.2, 44.4, 39.7, 27.5

1-(3,3-Dimethyl-4-(2,6,6-trimethylcyclohex-1-en-1-yl)but-1-en-2-yl)-4-methoxybenzene (2n)

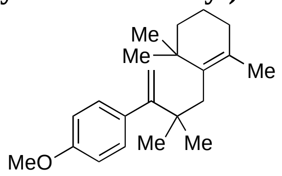

Diene prepared according to **general procedure A** using 2-(bromomethyl)-1,3,3-trimethylcyclohex-1-ene (prepared according to literature procedure[5]) as the electrophile in the first step. The final product was purified by silica gel column chromatography (20% Et_2O/hexanes) to furnish 1.10 g of pure product (50%) as a colorless oil. Analytical data for **2n**: **^{1}H NMR** (600 MHz, $CDCl_3$): δ 7.18 (d, *J* = 9.0 Hz, 2H), 6.82 (d, *J* = 9.0 Hz, 2H), 5.14 (d, *J* = 1.2 Hz, 1H), 4.81 (d, *J* = 1.2 Hz, 1H), 3.80 (s, 3H), 2.32 (s, 2H), 1.95 (t, *J* = 6.6 Hz, 2H), 1.56 (m, 2H), 1.55 (s, 3H), 1.40 (m, 2H), 1.14 (s, 3H), 0.94 (s, 6H); **^{13}C NMR** (150 MHz, $CDCl_3$); δ 158.4, 158.1, 143.6, 135.8, 130.0, 114.0, 113.2, 112.7, 55.2, 48.4, 39.3, 28.0, 25.3

(*E*)-1-(3,3-Dimethylhepta-1,5-dien-2-yl)-4-methoxybenzene (2o)

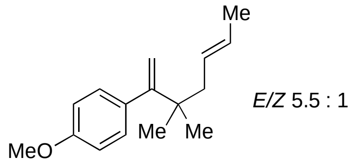

Diene prepared according to **general procedure A** using crotyl bromide as the electrophile in the first step. The final product was purified by silica gel column chromatography (20% diethyl ether/hexanes) to furnish 1.86 g of pure product (81%) as a colorless oil (5.5:1 mixture of isomers). Analytical data for **2o**: **^{1}H NMR** for *E* isomer (600 MHz, $CDCl_3$): δ 7.05 (d, *J* = 8.4 Hz, 2H), 6.80 (d, *J* = 8.4 Hz, 2H), 5.41 (m, 1H), 5.10 (d, *J* = 1.2 Hz, 1H), 4.82 (d, *J* = 1.2 Hz, 1H), 3.79 (s, 3H), 2.03 (m, 2H), 1.66 (m, 3H), 1.04 (s, 6H); **^{13}C NMR** for *E* isomer (150 MHz, $CDCl_3$); δ 158.1, 157.6, 135.8, 130.0, 128.0, 127.2, 113.3, 112.7, 55.2, 43.9, 39.3, 27.3, 18.1

1-Methoxy-4-(3,3,5-trimethylhexa-1,5-dien-2-yl)benzene (2p)

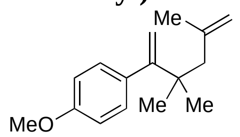

Diene prepared according to **general procedure A** using methallyl bromide as the electrophile in the first step. The final product was purified by silica gel column chromatography (20% EtOAc/hexanes) to furnish 1.55 g of pure product (67%) as a colorless oil. Analytical data for **2p**: **^{1}H NMR** (600 MHz, $CDCl_3$): δ 7.10 (d, *J* = 8.4 Hz, 2H), 6.81 (d, *J* = 8.4 Hz, 2H), 5.17 (s, 1H), 4.85 (s, 1H), 4.83 (s, 1H), 4.71 (s, 1H), 3.79 (s, 3H), 2.14 (s, 2H), 1.75 (s, 3H), 1.11 (s, 6H); **^{13}C NMR** (150 MHz, $CDCl_3$); δ 158.4, 158.1, 143.6, 135.8, 130.0, 114.0, 113.2, 112.7, 55.2, 48.4, 39.2, 28.0, 25.3

1-(3,3-Dimethylhexa-1,5-dien-2-yl)-4-methoxybenzene (2q)

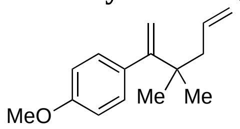

Diene prepared according to **general procedure A** using allyl bromide as the electrophile in the first step. The final product was purified by silica gel column chromatography (20% EtOAc/hexanes) to furnish 1.94 g of pure product (90%) as a colorless oil. Analytical data for **2q**: **^{1}H NMR** (600 MHz, $CDCl_3$): δ 7.06 (d, J = 7.8 Hz, 2H), 6.81 (d, J = 7.8 Hz, 2H), 5.81 (m, 1H), 5.12 (s, 1H), 5.02 (m, 2H), 4.84 (s, 1H), 3.79 (s, 3H), 2.11 (d, J = 7.2 Hz, 2H), 1.07 (s, 6H); **^{13}C NMR** (150 MHz, $CDCl_3$); δ 158.1, 157.3, 135.8, 135.6, 130.0, 116.7, 113.5, 112.7, 55.2, 45.2, 39.1, 27.3

General procedure B: Preparation of substrates with methylene tether

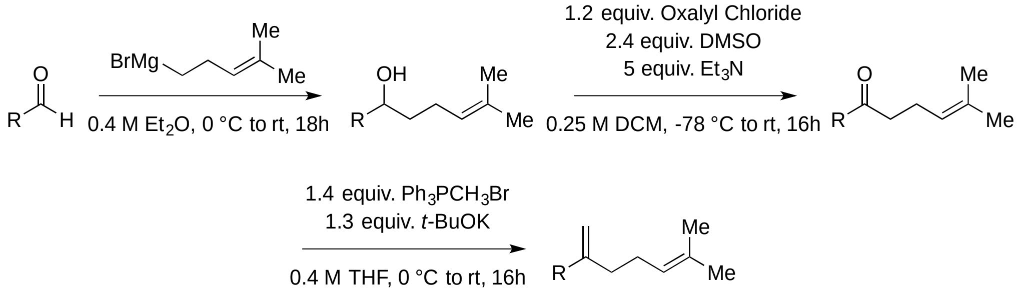

Freshly activated and dried magnesium turnings (1.38 equiv relative to aldehyde) were weighed into a flame-dried round bottom flask equipped with a stir bar. The flask was purged with nitrogen, cooled to 0 °C, and diethyl ether dispensed (to approximate concentration 0.6 mmol/mL). Seventy microliters 1,2-dibromoethane were added to activate the magnesium and stirred for 15 minutes. At 0 °C, 5-bromo-2-methyl-2-pentene (1.25 equiv relative to aldehyde) was added dropwise to the stirring magnesium turnings and stirred for 2 hours (consumption of magnesium observed). After formation of the Grignard reagent, the necessary aldehyde (freshly distilled to ensure purity), in diethyl ether, was added to the reaction mixture dropwise at 0 °C and stirred for 18 hours. The reaction was quenched with concentrated NH_4Cl solution dropwise and diluted with DI water and diethyl ether. The organic layer was separated and aqueous layer extracted three times with diethyl ether in a separatory funnel. The combined organics were washed with brine, dried with anhydrous $MgSO_4$, filtered, and concentrated to afford a crude oil, which was taken on to the next step without further purification (acceptable purity confirmed by TLC and ^{1}H NMR).

The crude alkenol product was then converted to the aryl ketone via Swern oxidation. A flame-dried round bottom flask containing a stir bar was charged with dry DCM (to approximate concentration of 0.25 mmol/mL) and cooled to −78 °C. At −78 °C, the flask was charged with oxalyl chloride (1.2 equiv) and stirred. After 10 minutes, DMSO (2.4 equiv) was added to the flask dropwise then stirred for 25 minutes at −78 °C. The alkenol substrate was next added to the reaction mixture dropwise and stirred for additional 25 minutes at −78 °C. Finally, triethylamine (5 equiv) was added to the reaction dropwise. The reaction was warmed to room temperature and stirred overnight. The reaction was quenched with concentrated NH_4Cl, the organic layer was separated, and the aqueous layer extracted three times with DCM. The combined organics were

washed with brine, dried with anhydrous $MgSO_4$, filtered, and concentrated to afford the crude aryl ketone, which was further purified by silica gel column chromatography.

The purified aryl ketone was converted to the desired unconjugated diene by Wittig olefination. Wittig olefination procedure for general procedure B is identical to Wittig olefination procedure in **general procedure A** (step 2).

(7-Methyl-3-methyleneoct-6-en-1-yl)benzene (2d)

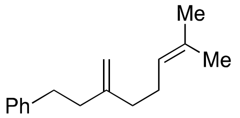

Diene prepared according to **general procedure B** using hydrocinnamaldehyde in the Grignard addition step. The alkyl ketone product from oxidation was purified by silica gel column chromatography (20% EtOAc/hexanes). The final product was purified using silica gel column chromatography (20% EtOAc/hexanes) to furnish 2.06 g of the desired diene (68%) as a colorless oil. Analytical data for **2d**: **^{1}H NMR** (600 MHz, $CDCl_3$): δ 7.27 (m, 2H), 7.18 (m, 3H), 5.12 (m, 1H), 4.76 (s, 2H), 2.74 (t, *J* = 7.8 Hz, 2H), 2.32 (t, *J* = 8.4 Hz, 2H), 2.12 (m, 2H), 2.07 (m, 2H), 1.69 (s, 3H), 1.61 (s, 3H); **^{13}C NMR** (150 MHz, $CDCl_3$); δ 149.1, 142.3, 131.7, 128.3, 128.3, 125.7, 124.1, 109.2, 38.0, 36.3, 34.7, 26.5, 25.7, 17.7

1-Methoxy-4-(6-methylhepta-1,5-dien-2-yl)benzene (2f)

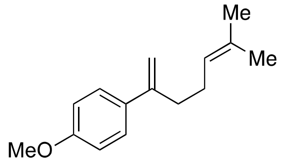

Diene prepared according to **general procedure B** using 4-anisaldehyde in the Grignard addition step. The aryl ketone product from oxidation was purified by silica gel column chromatography (20% EtOAc/hexanes). The final product was purified using silica gel column chromatography (20% Et_2O/hexanes) to furnish 2.46 g of the desired diene (59%) as a colorless oil. Spectral data were in agreement with the reported literature values.[6] Analytical data for **2f**: **^{1}H NMR** (600 MHz, $CDCl_3$): δ 7.35 (d, J = 9.0 Hz, 2H), 6.85 (d, J = 9.0 Hz, 2H), 5.20 (s, 1H), 5.14 (m, 1H), 4.97 (s, 1H), 3.80 (s, 3H), 2.48 (t, J = 7.2 Hz, 2H), 2.13 (q, J = 7.2 Hz, J = 7.8 Hz, 2H), 1.68 (s, 3H), 1.55 (s, 3H); **^{13}C NMR** (150 MHz, $CDCl_3$); δ 158.9, 147.6, 133.7, 131.8, 127.1, 123.9, 113.5, 110.6, 55.2, 35.5, 27.0, 25.7, 17.7

1,2-Dimethoxy-4-(6-methylhepta-1,5-dien-2-yl)benzene (2h)

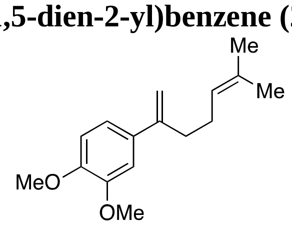

Diene prepared according to **general procedure B** using 3,4-dimethoxybenzaldehyde in the Grignard addition step. The aryl ketone product from oxidation was purified by silica gel column chromatography (20% EtOAc/hexanes). The final product was purified using silica gel column chromatography (20% Et_2O/hexanes) to furnish 1.29 g of the desired diene (42%) as a colorless oil. Analytical data for **2h**: **^{1}H NMR** (600 MHz, $CDCl_3$): δ 6.95 (m, 2H), 6.81 (d, *J* =

8.4 Hz, 1H), 5.20 (d, J = 1.8 Hz, 1H), 5.14 (m, 1H), 4.98 (d, J = 1.8 Hz, 1H), 3.88 (s, 3H), 3.87 (s, 3H), 2.47 (t, J = 7.2 Hz, 2H), 2.13 (q, J = 7.2 Hz, 2H), 1.67 (s, 3H), 1.54 (s, 3H); **^{13}C NMR** (150 MHz, $CDCl_3$); δ 148.6, 148.5, 147.9, 134.2, 131.8, 123.9, 118.4, 110.9, 110.8, 109.4, 55.9, 55.8, 35.5, 27.0, 25.7, 17.7

1-Methoxy-2-(6-methylhepta-1,5-dien-2-yl)benzene (2i)

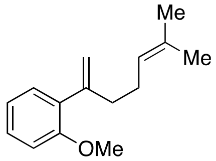

Diene prepared according to **general procedure B** using *o*-anisaldehyde in the Grignard addition step. The aryl ketone product from oxidation was purified by silica gel column chromatography (10% EtOAc/hexanes). The final product was purified using silica gel column chromatography (hexanes) to furnish 1.58 g of the desired diene (47%) as a colorless oil. Spectral data were in agreement with the reported literature values.[7] Analytical data for **2i**: **^{1}H NMR** (600 MHz, $CDCl_3$): δ 7.24 (m, 1H), 7.13 (m, 1H), 6.91 (m, 1H), 6.86 (m, 1H), 5.14 (s, 1H), 5.12 (m, 1H), 5.02 (s, 1H), 3.82 (s, 3H), 2.51 (t, J = 7.8 Hz, 2H), 2.04 (q, J = 7.8 Hz, 2H), 1.67 (s, 3H), 1.53 (s, 3H); **^{13}C NMR** (150 MHz, $CDCl_3$); δ 156.5, 149.0, 132.1, 131.4, 130.2, 128.2, 124.2, 120.4, 114.0, 110.5, 55.4, 36.3, 26.8, 25.7, 17.6

1-Methyl-4-(6-methylhepta-1,5-dien-2-yl)benzene (2j)

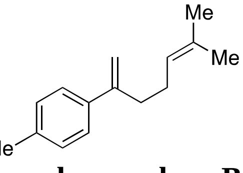

Diene prepared according to **general procedure B** using 4-tolualdehyde in the Grignard addition step. The aryl ketone product from oxidation was purified by silica gel column chromatography (10% Et_2O/hexanes). The final product was purified using silica gel column chromatography (hexanes) to furnish 1.27 g of the desired diene (52%) as a colorless oil. Spectral data were in agreement with the reported literature values.[7] Analytical data for **2j**: **^{1}H NMR** (600 MHz, $CDCl_3$): δ 7.31 (d, J = 7.8 Hz, 2H), 7.13 (d, J = 7.8 Hz, 2H), 5.25 (d, J = 1.2 Hz, 1H), 5.15 (m, 1H), 5.01 (d, J = 1.2 Hz, 1H), 2.49 (dt, J = 7.8 Hz, J = 1.2 Hz, 2H), 2.34 (s, 3H), 2.13 (q, J = 7.8 Hz, 2H), 1.68 (s, 3H), 1.55 (s, 3H); **^{13}C NMR** (150 MHz, $CDCl_3$); δ 148.1, 138.3, 137.0, 131.8, 128.9, 125.9, 123.9, 111.4, 35.4, 27.0, 25.7, 21.1, 17.7

1-Chloro-4-(6-methylhepta-1,5-dien-2-yl)benzene (2k)

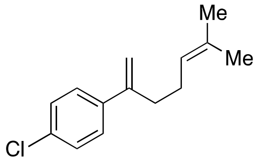

Diene prepared according to **general procedure B** using 4-chlorobenzaldehyde in the Grignard addition step. The aryl ketone product from oxidation was purified by silica gel column chromatography (5% Et_2O/hexanes). The final product was purified using silica gel column chromatography (hexanes) to furnish 1.44 g of the desired diene (40%) as a colorless oil. Analytical data for **2k**: **^{1}H NMR** (600 MHz, $CDCl_3$): δ 7.32 (d, J = 9.0 Hz, 2H), 7.27 (d, J = 9.0

Hz, 2H), 5.25 (d, J = 1.2 Hz, 1H), 5.11 (m, 1H), 5.06 (d, J = 1.2 Hz, 1H), 2.47 (t, J = 7.8 Hz, 2H), 2.10 (q, J = 7.8 Hz, 2H), 1.66 (s, 3H), 1.53 (s, 3H); **^{13}C NMR** (150 MHz, $CDCl_3$); δ 147.0, 139.5, 132.8, 131.8, 128.1, 127.2, 123,4, 112.5, 35.1, 26.6, 25.5, 17.5

2-(6-Methylhepta-1,5-dien-2-yl)furan (2l)

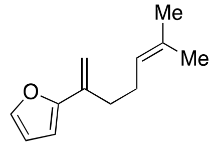

Diene prepared according to **general procedure B** using furfural in the Grignard addition step. The aryl ketone product from oxidation was purified by silica gel column chromatography (10% Et_2O/hexanes). The final product was purified using silica gel column chromatography (hexanes) to furnish 0.61 g of the desired diene (14%) as a yellow oil. Analytical data for **2l**: **^{1}H NMR** (600 MHz, $CDCl_3$): δ 7.34 (d, J = 1.8 Hz, 1H), 6.36 (m, 1H), 6.29 (d, J = 3.6 Hz, 1H), 5.51 (s, 1H), 5.16 (m, 1H), 4.96 (s, 1H), 2.36 (t, J = 7.8 Hz, 2H), 2.23 (q, J = 7.8 Hz, 2H), 1.68 (s, 3H), 1.59 (s, 3H); **^{13}C NMR** (150 MHz, $CDCl_3$); δ 154.8, 141.7, 137.3, 132.1, 123.7, 111.0, 109.2, 105.8, 33.3, 27.3, 25.7, 17.7

2,4-Dimethoxy-1-(6-methylhepta-1,5-dien-2-yl)benzene (2g)

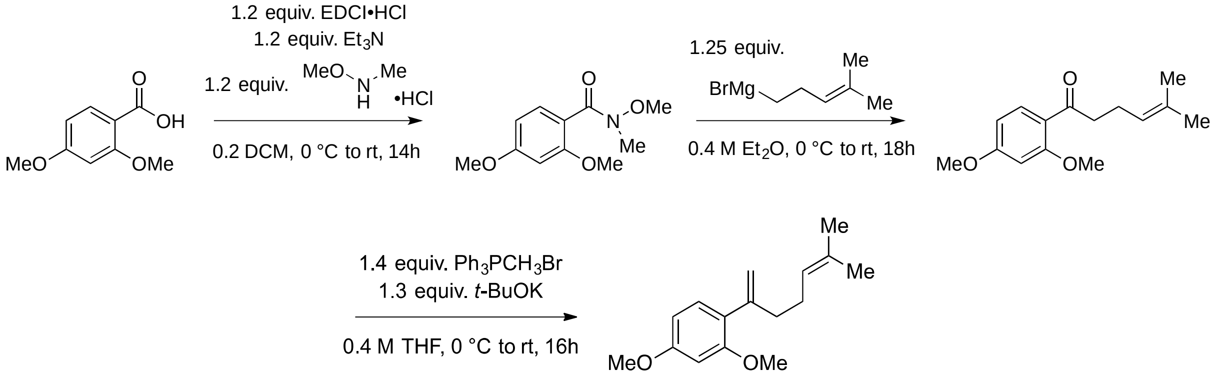

A flame-dried round bottom flask, containing 2,4-dimethoxybenzoic acid (1.0 equiv) and *N,O*-dimethylhydroxylamine hydrochloride (1.2 equiv) dissolved in anhydrous dichloromethane (0.2 M), was cooled to 0 °C with stirring. At 0 °C, triethylamine (1.2 equiv) was added to the solution dropwise via syringe and stirred for 15 minutes. Next, at 0 °C, 1-ethyl-3-(3-dimethylaminopropyl)carbodiimide hydrochloride (EDCI•HCl, 1.2 equiv) was added to the stirring solution in one portion. The reaction was allowed warm to room temperature and stir overnight. The reaction was then quenched with 2 N HCl and diluted with DCM. The organic layer was separated and washed twice with DI water. The combined organics were then washed with brine, dried with anhydrous $MgSO_4$, filtered, and concentrated to afford a crude oil, which was taken on to the Grignard addition without further purification.

Grignard addition into the Weinreb amide and Wittig olefination were carried out according the equivalent procedures in **general procedure B**. The aryl ketone produced from Grignard addition into the Weinreb amide was purified by column chromatography (20% EtOAc/hexanes) to yield a colorless oil (1.76 g, 47%). The Wittig olefination product was purified by column chromatography (20% Et_2O/hexanes) to give 1.42 g (81%) of the desired unconjugated diene as a colorless oil. Analytical data for **2g**: **^{1}H NMR** (600 MHz, $CDCl_3$): δ 7.03 (m, 1H), 6.42 (m, 2H), 5.08 (m, 2H), 4.96 (d, J = 2.4 Hz, 1H), 3.79 (s, 3H), 3.78 (s, 3H),

2.45 (t, *J* = 7.8 Hz, 2H), 2.00 (q, *J* = 7.8 Hz, 2H), 1.64 (s, 3H), 1.51 (s, 3H); **^{13}C NMR** (150 MHz, $CDCl_3$); δ 160.0, 157.5, 148.5, 131.3, 130.5, 124.8, 124.3, 113.9, 103.9, 98.5, 55.4, 55.3, 36.5, 26.9, 25.7, 17.6

(*Z*)-1-(Hepta-1,5-dien-2-yl)-4-methoxybenzene (2r)

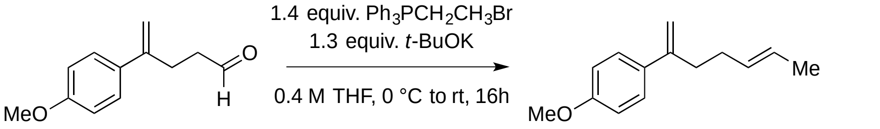

8.5 : 1 as measured by ^{1}H NMR integrations

4-(4-methoxyphenyl)pent-4-enal was prepared according to a procedure published by Hartwig and coworkers.[8] Wittig olefination of the terminal aldehyde carried out using ethyltriphenylphosphonium bromide following the procedure in **general procedure A** above. The desired diene product was purified by column chromatography (20% Et_2O/hexanes) to furnish 1.13 g (67%) of pure product as a colorless oil (8.5:1 mixture of *E*/*Z* isomers). Analytical data for **2r**: **^{1}H NMR** (400 MHz, $CDCl_3$): δ 7.34 (d, *J* = 8.8 Hz, 2H), 6.85 (d, *J* = 8.8 Hz, 2H), 5.41 (m, 2H), 5.21 (d, *J* = 1.6 Hz, 1H), 4.98 (d, *J* = 1.6 Hz, 1H), 3.80 (s, 3H), 2.51 (m, 2H), 2.20 (m, 2H), 1.53 (d, *J* = 5.6 Hz, 3H); **^{13}C NMR** (100 MHz, $CDCl_3$); δ 158.9, 147.3, 133.5, 129.8, 127.1, 124.1, 113.5, 110.7, 55.1, 35.1, 25.7, 12.7

1-(Hexa-1,5-dien-2-yl)-4-methoxybenzene (2s)

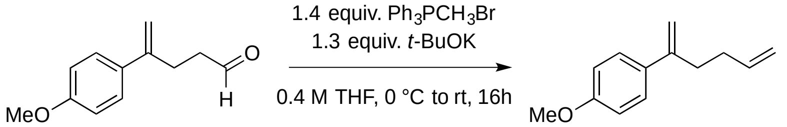

4-(4-methoxyphenyl)pent-4-enal was prepared according to a procedure published by Hartwig and coworkers.[8] Wittig olefination of the terminal aldehyde carried out using methyltriphenylphosphonium bromide following the procedure in **general procedure A** above. The diene product was purified by column chromatography (20% Et_2O/hexanes) to furnish 1.13 g of pure product (75%) as a colorless oil. Spectral data were in agreement with the reported literature values.[9] Analytical data for **2s**: **^{1}H NMR** (600 MHz, $CDCl_3$): δ 7.24 (d, *J* = 7.2 Hz, 2H), 6.85 (d, *J* = 7.2 Hz, 2H), 5.83 (m, 1H), 5.21 (s, 1H), 4.98 (m, 3H), 3.80 (s, 3H), 2.56 (t, *J* = 7.8 Hz, 2H), 2.20 (q, *J* = 7.8 Hz, 2H); **^{13}C NMR** (150 MHz, $CDCl_3$); δ 159.0, 147.1, 138.2, 133.6, 127.2, 114.6, 113.6, 110.9, 55.2, 34.8, 32.5

1-methoxy-4-(3-((3-methylbut-2-en-1-yl)oxy)prop-1-en-2-yl)benzene (2t)

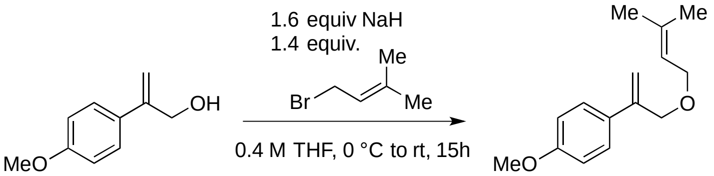

1.6 equiv sodium hydride was dispensed into a flame-dried round bottom flask, which was subsequently purged with N_2. The sodium hydride was suspended in dry THF (0.4 mmol/mL relative to substrate) and the stirred suspension was cooled to 0 °C in an ice bath. At 0 °C, a solution of 2-(4-methoxyphenyl)prop-2-en-1-ol (prepared according to literature procedure[10]) in THF was added dropwise with stirring. The solution was stirred at 0 °C for 30 minutes after effervescence ceased. At 0 °C, prenyl bromide (1.4 equiv) was added to the mixture dropwise.

The reaction was allowed to warm to room temperature and stirred for 15 hours overnight. The reaction was then quenched with concentrated NH_4Cl solution, and diluted with DI water and diethyl ether. The organic layer was separated and aqueous layer extracted three times with diethyl ether in a separatory funnel. The combined organics were washed with brine, dried with anhydrous $MgSO_4$, filtered, and concentrated to afford a crude oil. The crude allylic ether was purified by silica gel column chromatography (20% Et_2O/hexanes) to yield 2.16 g (70%) of the desired product as an off-white oil. Analytical data for **2t**: **^{1}H NMR** (600 MHz, $CDCl_3$): δ 7.41 (d, *J* = 8.4 Hz, 2H), 6.85 (d, *J* = 8.4 Hz, 2H), 5.43 (d, *J* = 1.2 Hz, 1H), 5.35 (m, 1H), 5.22 (d, *J* = 1.2 Hz, 1H), 4.32 (s, 2H), 3.98 (d, *J* = 7.2 Hz, 2H), 3.79 (s, 3H), 1.72 (s, 3H), 1.62 (s, 3H); **^{13}C NMR** (150 MHz, $CDCl_3$); δ 159.2, 143.7, 137.1, 131.4, 127.2, 121.0, 113.6, 112.7, 72.0, 65.3, 55.3, 25.8, 18.0

1-((*E*)-3-(Cinnamyloxy)prop-1-en-1-yl)-4-methoxybenzene (2u)

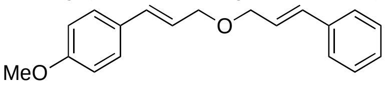

Prepared according to a published procedure; spectral data were in agreement with the literature values.[11]

(*E*)-1-Methoxy-4-(3-((3-methylbut-2-en-1-yl)oxy)prop-1-en-1-yl)benzene (2v)

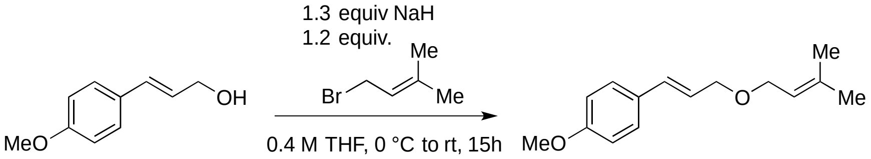

Prepared according to published procedure for **2u** above, however using 3,3-dimethylallyl bromide in place of cinnamyl bromide. The desired product was purified by column chromatography (10% Et_2O/hexanes) to furnish 1.81 g of pure product (78%) as a yellow oil. Analytical data for **2v**: **^{1}H NMR** (600 MHz, $CDCl_3$): δ 7.30 (d, *J* = 8.4 Hz, 2H), 6.83 (d, *J* = 8.4 Hz, 2H), 6.53 (d, *J* = 16.2 Hz, 1H), 6.16 (m, 1H), 5.38 (m, 1H), 4.09 (dd, *J* = 6.6 Hz, *J* = 1.8 Hz, 2H), 3.99 (d, *J* = 6.6 Hz, 2H), 3.78 (s, 3H), 1.74 (s, 3H), 1.67 (s, 3H); **^{13}C NMR** (150 MHz, $CDCl_3$); δ 159.2, 137.0, 132.0, 129.5, 127.6, 124.0, 121.1, 113.9, 70.8, 66.4, 55.2, 25.8, 18.0

((1*E*,1'*E*)-Oxybis(prop-1-ene-3,1-diyl))dibenzene (2w)

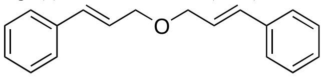

Prepared according to a published procedure; spectral data were in agreement with the literature values.[11]

VI. General procedures for PET endoperoxidation

Isolated products of cyclization–endoperoxidation cascade reactions

Procedure for synthesis of bicyclic endoperoxide 3a from diene 2a

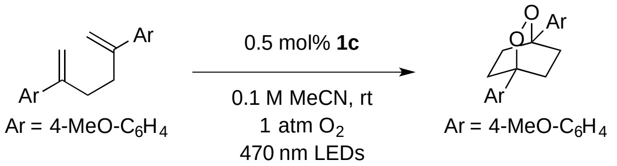

A flame dried 1-dram vial equipped with a magnetic stir bar and 0.25 mg and 2,4,6-tris(4-methoxyphenyl)pyrylium tetrafluoroborate catalyst, then sealed with a septum cap. 0.5 mL dry acetonitrile was then added via syringe under an atmosphere of nitrogen. Finally, 30 mg diene **3a** was added to the stirring reaction mixture in 0.5 mL acetonitrile. A balloon of oxygen was fitted to the vial and bubbled through the stirring solution for 2 minutes. After bubbling was suspended, an atmosphere of oxygen was maintained over the solution and the reaction was irradiated for two hours at ambient temperature using 470 LEDs. Upon completion, the desired product was isolated using silica gel column chromatography (33% Et_2O/hexanes). Spectral data were in agreement with the literature values.[12]

General procedure for endoperoxidation of unconjugated dienes under oxygen atmosphere.

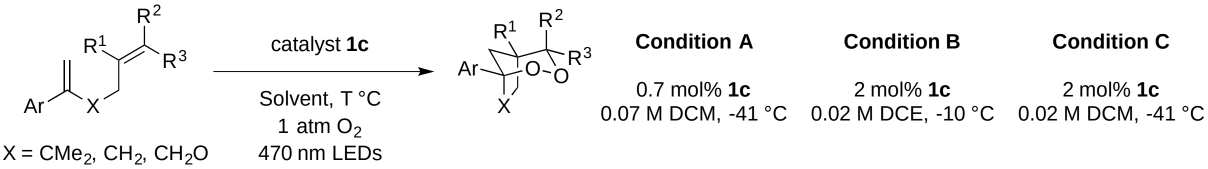

A flame dried flask (2-dram vial, scintillation vial, or round-bottom flask) was equipped with a magnetic stir bar and 2,4,6-tris(4-methoxyphenyl)pyrylium tetrafluoroborate catalyst (**1c**, 4-MeO-TPT, see conditions above for catalyst loading), then sealed with a septum. The solvent (DCM for conditions A and C, DCE for condition B, see conditions above for concentration) was then added to the flask via syringe under an atmosphere of nitrogen. Substrate was then added to the flask via microsyringe and stirred to ensure a homogeneous reaction mixture. A balloon of oxygen was fitted to the reaction flask and oxygen was bubbled through the stirring solution for 3 minutes to ensure oxygen saturation in the solvent. After bubbling was suspended the reaction was fitted with a balloon of oxygen to ensure an oxygen atmosphere and submerged in a temperature-controlled bath (−41 °C for conditions A and C, −10 °C for condition B). The reaction was irradiated with 470 nm LEDs until completion (as monitored by TLC). Upon completion, the reaction mixture was concentrated under reduced pressure and the products isolated by silica gel column chromatography.

1-(4-Methoxyphenyl)-4,4,7,7-tetramethyl-2,3-dioxabicyclo[3.2.1]octane (3a)

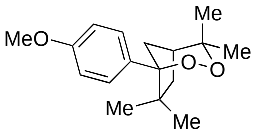

The average yield for the title compound was 66% (2 trials) at the 0.72 mmol scale, using **condition A**, and an irradiation time of 3.5 hours. The title compound was purified by column

chromatography on silica gel (10% EtOAc/hexanes) to yield a white solid. Analytical data for **3a**: **^{1}H NMR** (400 MHz, $CDCl_3$): δ 7.28 (d, *J* = 8.8 Hz, 2H), 6.84 (d, *J* = 8.8 Hz, 2H), 3.78 (s, 3H), 2.35 (dd, *J* = 12.0 Hz, *J* = 5.6 Hz, 1H), 2.24 (dd, *J* = 12.0 Hz, *J* = 2.8 Hz, 1H), 2.03 (t, *J* = 6.0 Hz, 1H), 1.98 (dd, *J* = 13.2 Hz, *J* = 2.8 Hz, 1H), 1.66 (dd, *J* = 13.2 Hz, *J* = 6.4 Hz), 1.41 (s, 3H), 1.32 (s, 3H), 1.13 (s, 3H), 0.58 (s, 3H); **^{13}C NMR** (150 MHz, $CDCl_3$) δ 159.2, 130.3, 127.5, 113.2, 93.4, 80.3, 55.2, 42.5, 42.4, 41.6, 33.7, 31.6, 24.9, 23.4, 21.6; **MS** (+ESI): *m/z* calculated for $C_{17}H_{24}O_3$ $[M+H]^+$ 277.18, found 277.11.

1-(4-Methoxyphenyl)-4,4-dimethyl-2,3-dioxabicyclo[3.2.1]octane (3f)

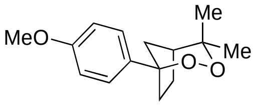

The average yield for the title compound was 32% (2 trials) at the 1.0 mmol scale, using **condition B**, and an irradiation time of 3.5 hours. The title compound was purified by column chromatography on silica gel (20% EtOAc/hexanes) to yield a white solid. Analytical data for **3f**: **^{1}H NMR** (400 MHz, $CDCl_3$): δ 7.35 (d, *J* = 8.8 Hz, 2H), 6.85 (d, *J* = 8.8 Hz, 2H), 3.78 (s, 3H), 2.49 (m, 2H), 2.16 (t, *J* = 5.4 Hz, 1H), 2.09 (m, 1H), 1.84 (dt, *J* = 12.4 Hz, *J* = 3.6 Hz, 1H), 1.74 (m, 2H), 1.45 (s, 3H), 1.16 (s, 3H); **^{13}C NMR** (150 MHz, $CDCl_3$) δ 159.4, 132.2, 127.9, 113.6, 90.0, 80.6, 55.2, 43.7, 35.6, 34.8, 26.3, 24.8, 23.4; **MS** (+ESI): *m/z* calculated for $C_{15}H_{20}O_3$ 249.15, found 249.15.

1-(2,4-Dimethoxyphenyl)-4,4-dimethyl-2,3-dioxabicyclo[3.2.1]octane (3g)

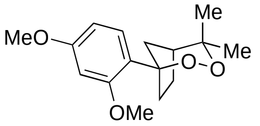

The average yield for the title compound was 9% (2 trials) at the 0.5 mmol scale, using **condition B**, and an irradiation time of 3 hours. The title compound was purified by column chromatography on silica gel (10% to 20% Et_2O/pentane) to yield a colorless oil. Analytical data for **3g**: **^{1}H NMR** (600 MHz, $CDCl_3$): δ 7.25 (s, 1H), 6.42 (m, 2H), 3.79 (s, 3H), 3.77 (s, 3H), 2.78 (m, 1H), 2.58 (dt, *J* = 11.4 Hz, *J* = 3.0 Hz, 1H), 2.09 (t, *J* = 5.4 Hz, 1H), 2.05 (m, 1H), 1.88 (m, 1H), 1.80 (dd, *J* = 11.4 Hz, *J* = 5.4 Hz, 1H), 1.66 m, 1H), 1.43 (s, 3H), 1.15 (s, 3H); **^{13}C NMR** (150 MHz, $CDCl_3$) δ 161.0, 160.0, 129.1, 120.4, 103.6, 99.4, 89.6, 80.6, 55.5, 55.3, 43.4, 36.2, 33.4, 26.3, 24.9, 23.5; **MS** (+ESI): *m/z* calculated for $C_{16}H_{22}O_4$ $[M+H]^+$ 279.15, found 279.15.

1-(2-methoxyphenyl)-4,4-dimethyl-2,3-dioxabicyclo[3.2.1]octane (3i)

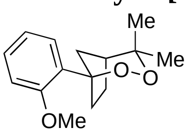

The average yield for the title compound was 16% (2 trials) at the 0.5 mmol scale, using **condition B**, and an irradiation time of 5 hours. The title compound was purified by column chromatography on silica gel (20% EtOAc/hexanes) to yield a white solid. Analytical data for **3i**: **^{1}H NMR** (600 MHz, $CDCl_3$): δ 7.34 (dd, *J* = 7.8 Hz, *J* = 1.8 Hz, 1H), 7.26 (m, 1H), 6.89 (m, 2H), 3.81 (s, 3H), 2.77 (m, 1H), 2.57 (dt, *J* = 12.0 Hz, *J* = 2.4 Hz, 1H), 2.10 (t, *J* = 5.4 Hz, 1H), 2.06 (m, 1H), 1.94 (m, 1H), 1.89 (dd, *J* = 11.4 Hz, *J* = 5.4 Hz, 1H), 1.69 (m, 1H), 1.44 (s, 3H),

1.16 (s, 3H); **^{13}C NMR** (150 MHz, $CDCl_3$) δ 158.7, 129.6, 128.2, 127.7, 120.2, 111.8, 89.9, 80.8, 55.6, 42.4, 36.1, 33.3, 26.3, 24.8, 23.5; **MS** (+ESI): *m/z* calculated for $C_{15}H_{20}O_3$ $[M+H]^+$ 249.15, found 249.09.

1-(4-Methoxyphenyl)-7,7-dimethyl-4-phenyl-2,3-dioxabicyclo[3.2.1]octane (3m)

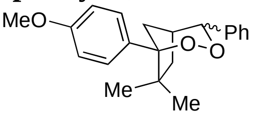

The average yield for the title compound was 68% (2 trials, 1:1 mixture of diastereomers) at the 0.5 mmol scale, using **condition C**, and an irradiation time of 3 hours. The title compound was purified by column chromatography on silica gel (10% Et_2O/pentane) to yield a white solid. Analytical data for **3m**: **^{1}H NMR** for diastereomer 1 (400 MHz, $CDCl_3$): δ 7.48 (d, J = 8.8 Hz, 2H), 7.31 (t, J = 7.2 Hz, 2H), 7.23 (m, 3H), 6.81 (d, J = 8.8 Hz, 2H), 5.0 (d, J = 3.6 Hz, 1H), 3.76 (s, 3H), 2.80 (m, 1H), 2.28 (dd, J = 12.0 Hz, J = 6.0 Hz, 1H), 2.07 (m, 2H), 1.83 (dd, J = 12.0 Hz, J = 2.0 Hz, 1H), 1.47 (s, 3H), 0.62 (s, 3H); **^{13}C NMR** for diastereomer 1 (150 MHz, $CDCl_3$) δ□159.3, 140.5, 130.4, 128.2, 127.5, 126.7, 126.1, 113.2, 94.9, 86.2, 55.2, 45.8, 43.4, 37.3, 32.0, 31.4, 21.6; **^{1}H NMR** for diastereomer 2 (400 MHz, $CDCl_3$): δ 7.35 (m, 4H), 7.27 (m, 3H), 6.87 (d, J = 8.8 Hz, 2H), 5.26 (s, 1H), 3.80 (s, 3H), 2.82 (dd, J = 11.6 Hz, J = 5.9 Hz, 1H), 2.59 (t, J = 6.3 Hz, 1H), 2.12 (dd, J = 11.6 Hz, J = 2.3 Hz, 1H), 1.87 (dd, J = 13.2 Hz, J = 2.3 Hz, 1H), 1.58 (dd, J = 13.2 Hz, J = 6.7 Hz, 1H); **^{13}C NMR** for diastereomer 2 (150 MHz, $CDCl_3$) δ□159.4, 138.8, 129.7, 128.2, 127.6, 127.5, 126.1, 113.3, 94.3, 85.5, 55.2, 43.6, 40.3, 39.4, 38.3, 31.6, 20.8; **MS** (+ESI): *m/z* calculated for $C_{21}H_{24}O_3$ $[M+H]^+$ 325.18, found 325.18

3-(4-Methoxyphenyl)-4,4,6,6,9a-pentamethylhexahydro-3*H*,6*H*-3,5a-methanobenzo[*c*][1,2]dioxepine (3n)

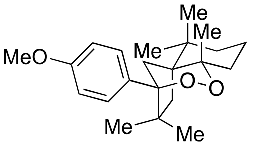

and

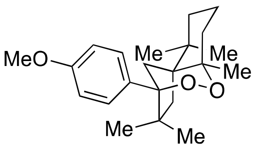

The average yield for the title compound was 64% (2 trials) at the 0.5 mmol scale, using **condition C**, and an irradiation time of 4 hours. The polycyclic product was isolated as 6.5:1 mixture of inseparable diastereomers (shown above, left thought to be major diastereomer). The title compound was purified by column chromatography on silica gel (10% Et_2O/pentane) to yield a colorless oil. Analytical data for **3n**: **^{1}H NMR** for major diastereomer (600 MHz, $CDCl_3$): δ 7.28 (d, J = 8.8 Hz, 2H), 6.83 (d, J = 8.8 Hz, 2H), 3.77 (s, 3H), 2.46 (d, J = 12.0 Hz, 1H), 2.31 (dt, J = 13.2 Hz, 5.4 Hz, 1H), 2.17 (dd, J = 12.0 Hz, 2.4 Hz, 1H), 1.84 (dd, J = 13.2 Hz, 2.5 Hz, 1H), 1.72-1.57 (m, 3H), 1.55 (d, J = 13.2 Hz, 1H), 1.48 (dt, J = 13.8 Hz, 4.8 Hz, 1H), 1.41 (dt, J = 13.2 Hz, 3.4 Hz, 1H), 1.31 (s, 3H), 1.25 (s, 3H), 1.01 (s, 3H), 0.96 (s, 3H), 0.57 (s, 3H); **^{13}C NMR** for major diastereomer (150 MHz, $CDCl_3$) δ□159.1, 130.9, 127.3, 113.2, 113.1, 92.8, 85.0, 55.2, 50.4, 43.8, 42.6, 37.0, 36.6, 35.5, 31.7, 30.7, 27.7, 25.3, 23.5, 21.7, 20.4; **MS** (+ESI): *m/z* calculated for $C_{22}H_{32}O_3$ $[M+H]^+$ 345.24, found 345.24

1-(4-Methoxyphenyl)-3-methylpent-4-en-1-one (3o)

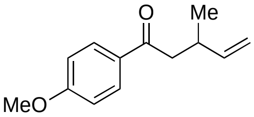

The yield for the title compound was 36% (1 trial) at the 0.5 mmol scale, using **condition C**, and an irradiation time of 23 hours (−41 °C to rt). The title compound was purified by column chromatography on silica gel (20% Et_2O/pentane) to yield a colorless oil. Analytical data for **3o**: **^{1}H NMR** (600 MHz, $CDCl_3$): δ 7.91 (d, *J* = 8.9 Hz, 2H), 6.91 (d, *J* = 8.9 Hz, 2H), 5.82 (m, 1H), 5.00 (d, *J* = 17.2 Hz, 1H), 4.92 (d, *J* = 10.4 Hz, 1H), 3.84 (s, 3H), 2.95 (dd, *J* = 14.9 Hz, *J* = 5.8 Hz, 1H), 2.86 (m, 1H), 2.81 (dd, *J* = 14.9 Hz, *J* = 7.2 Hz, 1H), 1.06 (d, *J* = 6.5 Hz, 1H); **^{13}C NMR** (150 MHz, $CDCl_3$) δ 197.9, 163.3, 143.2, 130.4, 130.3, 113.6, 112.9, 55.4, 44.8, 33.8, 19.8; **MS** (+ESI): *m/z* calculated for $C_{13}H_{17}O_2$ $[M+H]^+$ 205.12, found 205.09

1-(4-Methoxyphenyl)-4-methylpent-4-en-1-one (3p)

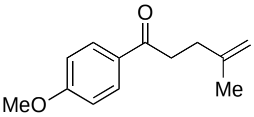

The yield for the title compound was 37% (1 trial) at the 0.5 mmol scale, using **condition C**, and an irradiation time of 23 hours (-41 °C to rt). The title compound was purified by column chromatography on silica gel (20% Et_2O/pentane) to yield a colorless oil. Spectral data were in agreement with the literature values.[13] Analytical data for **3p**: **^{1}H NMR** (600 MHz, $CDCl_3$): δ 7.93 (d, *J* = 8.9 Hz, 2H), 6.91 (d, *J* = 8.9 Hz, 2H), 4.74 (s, 1H), 4.70 (s, 1H), 3.84 (s, 3H), 3.04 (m, 2H), 2.41 (t, *J* = 7.8 Hz, 2H), 1.76 (s, 3H); **^{13}C NMR** (150 MHz, $CDCl_3$) δ□198.3, 163.3, 144.8, 130.3, 130.0, 113.7, 110.0, 55.4, 36.4, 32.1, 22.7

1-(4-Methoxyphenyl)pent-4-en-1-one (3q)

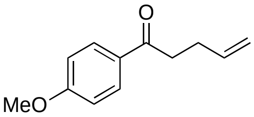

The yield for the title compound was 27% (1 trial) at the 0.5 mmol scale, using **condition C**, and an irradiation time of 23 hours (−41 °C to rt). The title compound was purified by column chromatography on silica gel (20% Et_2O/pentane) to yield a colorless oil. Spectral data were in agreement with the literature values.[13] Analytical data for **3q**: **^{1}H NMR** (600 MHz, $CDCl_3$): δ 7.93 (d, *J* = 8.9 Hz, 2H), 6.91 (d, *J* = 8.9 Hz, 2H), 5.88 (m, 1H), 5.06 (dq, *J* = 17.1, *J* = 1.8 Hz, 1H), 4.98 (dq, *J* = 10.2, *J* = 1.8 Hz, 1H), 3.85 (s, 3H), 3.00 (t, *J* = 7.2 Hz, 2H), 2.47 (m, 2H); **^{13}C NMR** (150 MHz, $CDCl_3$) δ 198.0, 163.4, 137.5, 130.3, 130.0, 115.1, 113.7, 55.4, 37.4, 28.3

1-(4-Methoxyphenyl)-4,4-dimethyl-2,3,7-trioxabicyclo[3.3.1]nonane (3t)

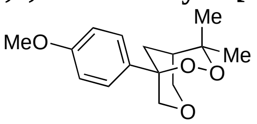

The average yield for the title compound was 16% (2 trials) at the 0.5 mmol scale, using modified **condition C** (0.04 M DCE, other conditions unaltered), and an irradiation time of 4 hours. The title compound was purified by column chromatography on silica gel (20% Et_2O/pentane) to yield a white solid. Analytical data for **3t**: **^{1}H NMR** (400 MHz, $CDCl_3$): δ 6.96

(d, J = 8.7 Hz, 2H), 6.82 (d, J = 8.6 Hz, 2H), 4.19 (m, 2H), 4.01 (dd, J = 9.6 Hz, J = 2.4 Hz, 1H), 3.77 (s, 3H), 3.72 (d, J = 9.6 Hz, 1H), 2.53 (m, 1H), 1.97 (dt, J = 6.7 Hz, J = 2.0 Hz, 1H), 1.87 (d, J = 9.2 Hz, 1H), 1.26 (s, 3H), 0.93 (s, 3H); **^{13}C NMR** (100 MHz, $CDCl_3$) δ□158.1, 134.5, 127.2, 113.6, 74.6, 68.3, 55.2, 50.1, 40.9, 39.3, 30.4, 23.6, 19.7; **MS** (GC-MS): *m/z* calculated for $C_{15}H_{20}O_4$ 264.14, found 264.1

1-(4-Methoxyphenyl)-4-phenyltetrahydro-1*H*,4*H*-furo[3,4-*d*][1,2]dioxine (3u)

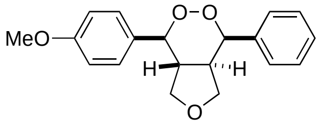

The average yield for the title compound was 79% (2 trials, 5.7:1 mixture of diastereomers) at the 0.5 mmol scale, using **condition C**, and an irradiation time of 3 hours. The title compound was purified by column chromatography on silica gel (33% to 50% EtOAc/hexanes) to yield a colorless oil. Spectral data were in agreement with the literature values.[11] Analytical data for **3u**: **^{1}H NMR** major diastereomer (600 MHz, $CDCl_3$): δ 7.49 (d, J = 7.0 Hz, 2H), 7.36 (t, J = 7.5 Hz, 2H), 7.30 (m, 3H), 6.90 (d, J = 8.7 Hz, 2H), 5.55 (d, J = 5.8 Hz, 1H), 5.03 (d, J = 9.9 Hz, 1H), 4.16 (t, J = 7.3 Hz, 1H), 3.80 (s, 3H), 3.77 (m, 1H), 3.42 (dd, J = 11.4 Hz, J = 8.4 Hz, 1H), 3.24 (dd, J = 11.4 Hz, J = 7.8 Hz, 1H), 2.83 (m, 1H), 2.60 (m, 1H); **^{1}H NMR** minor diastereomer (600 MHz, $CDCl_3$): δ 7.37 (s, 5H), 7.33 (d, J = 8.7 Hz, 2H), 6.90 (d, J = 8.7 Hz, 2H), 5.14 (d, J = 9.4 Hz, 1H), 5.11 (d, J = 9.4 Hz, 1H), 3.90 (m, 2H), 3.80 (s, 3H), 3.53 (m, 2H), 2.63 (m, 2H)

VII. Hydroperoxide via $^1O_2$

$^1O_2$ Control Experiment (Table 1, entry 10)

(*E*)-1-(6-Hydroperoxy-3,3,6-trimethylhepta-1,4-dien-2-yl)-4-methoxybenzene

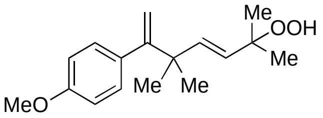

The yield for the title compound was 65% (1 trial) at the 0.36 mmol scale, using modified **condition A**, and an irradiation time of 3.5 hours. Rose Bengal was used in place of catalyst **1c** and dry MeOH as solvent in place of DCM. The reaction was carried out at ambient temperature. The title compound was purified by column chromatography on silica gel (20% Et_2O/hexanes) to yield a colorless oil. It should be noted that the shown tertiary allylic hydroperoxide was the major product in a transformation possibly producing multiple oxidation products (complete consumption of starting material was observed by ^{1}H NMR). No endoperoxidation/cyclization products were observed. Analytical data for shown hydroperoxide: **^{1}H NMR** (600 MHz, $CDCl_3$): δ 7.16 (s, 1H) 7.07 (d, J = 8.7 Hz, 2H), 6.77 (d, J = 8.7 Hz, 2H), 5.78 (d, J = 16.2 Hz, 1H), 5.42 (d, J = 16.2 Hz, 1H), 5.17 (d, J = 1.5 Hz, 1H), 4.90 (d, J = 1.5 Hz, 1H), 3.77 (s, 3H), 1.32 (s, 6H), 1.19 (s, 6H); **^{13}C NMR** (150 MHz, $CDCl_3$) δ□158.3, 156.6, 140.4, 135.2, 130.0, 129.8, 113.1, 112.7, 82.3, 55.2, 41.6, 27.2, 24.3; **MS** (+ESI): *m/z* calculated for $C_{17}H_{24}O_3$ $[M+H]^+$ 277.18, found 277.11

VIII. ^{1}H and ^{13}C NMR Spectra

1-Methoxy-4-(3,3,6-trimethylhepta-1,5-dien-2-yl)benzene (2a)

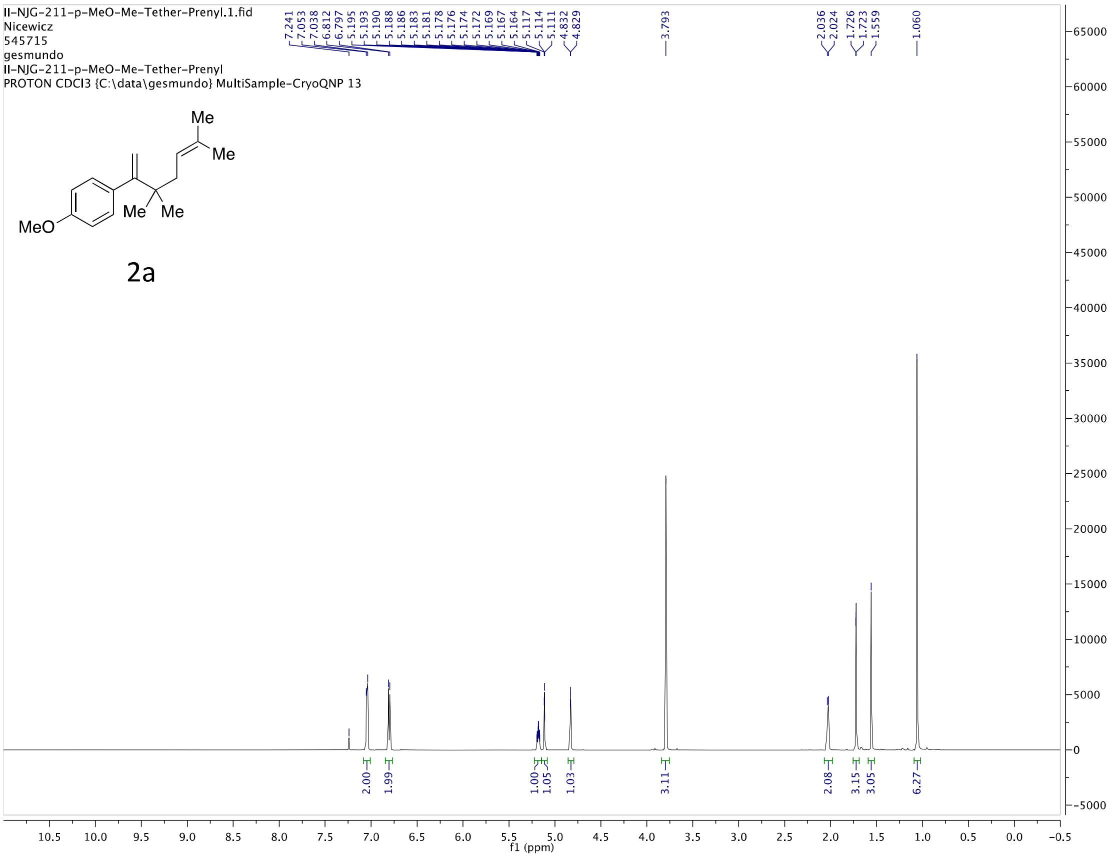

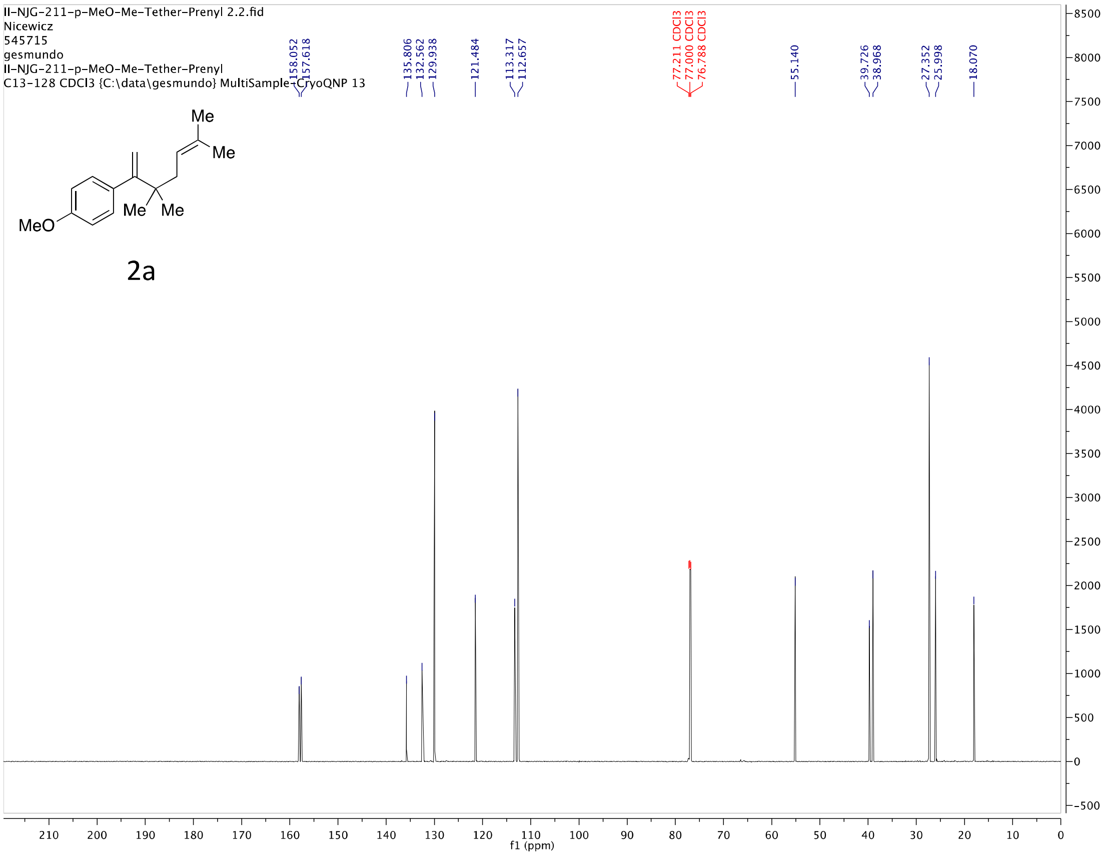
II-NJG-211-p-MeO-Me-Tether-Prenyl 2.2.fid
Nicewicz
545715
gesmundo
II-NJG-211-p-MeO-Me-Tether-Prenyl
C13-128 CDCl3 {C:\data\gesmundo} MultiSample-CryoQNP 13
158.052
157.618
135.806
132.562
129.938
121.484
113.317
112.657
77.211 CDCl3
77.000 CDCl3
76.788 CDCl3
55.140
39.726
38.968
27.352
25.998
18.070
MeO
Me
Me
Me
Me
2a
f1 (ppm)

(5-Methylhexa-1,5-dien-2-yl)benzene (2c)

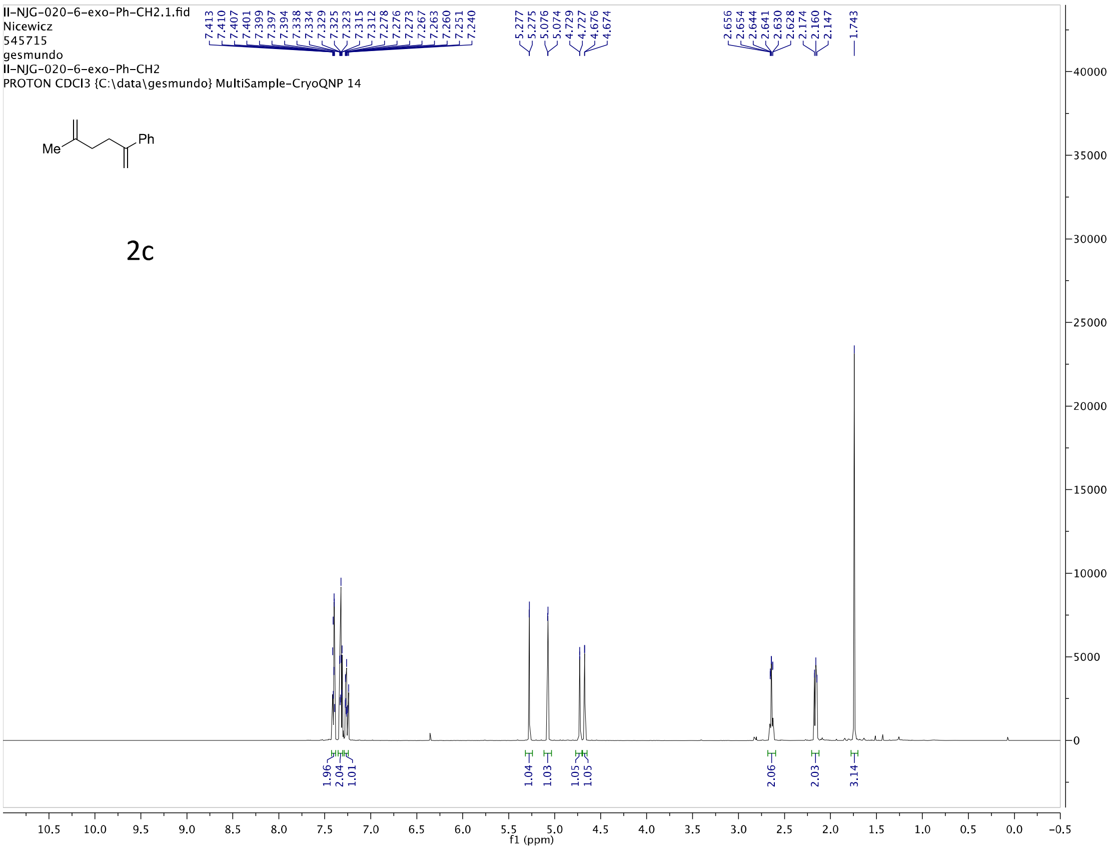

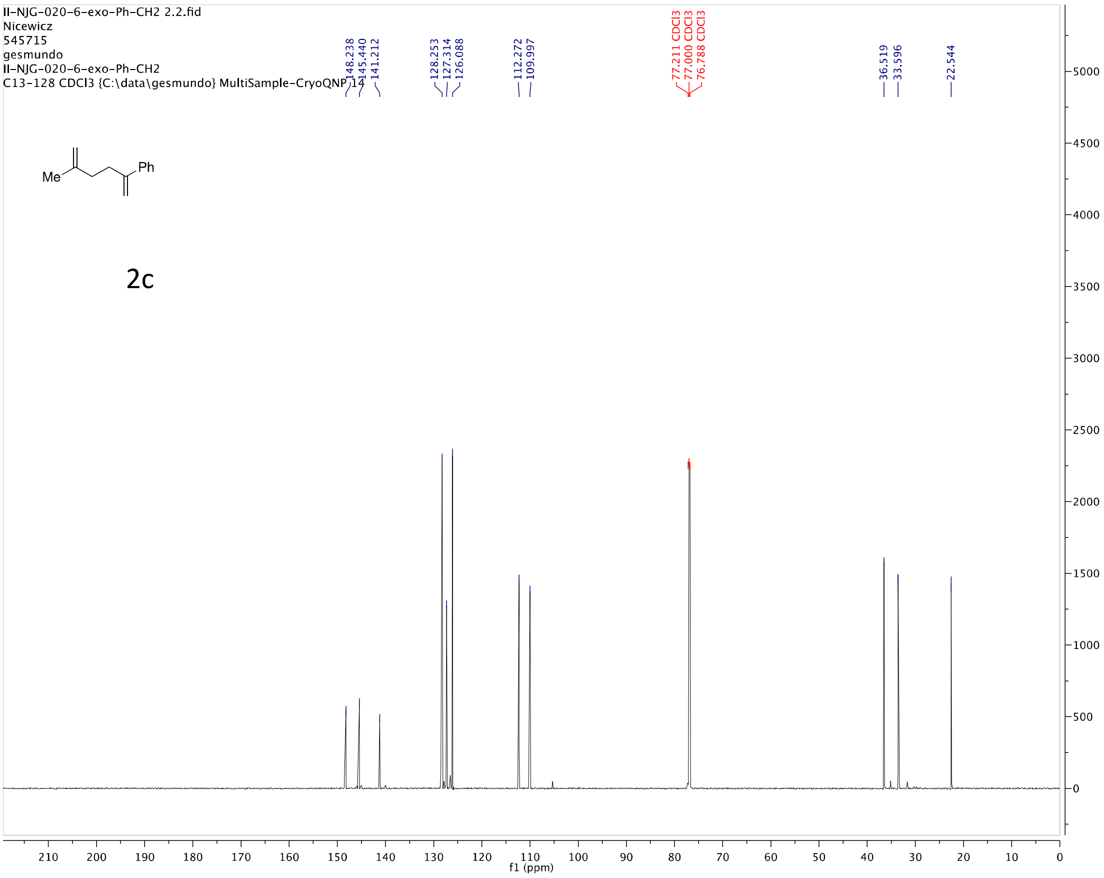
II-NJG-020-6-exo-Ph-CH2 2.2.fid
Nicewicz
545715
gesmundo
II-NJG-020-6-exo-Ph-CH2
C13-128 CDCl3 {C:\data\gesmundo} MultiSample-CryoQNP 14
148.238
145.440
141.212
128.253
127.314
126.088
112.272
109.997
77.211 CDCl3
77.000 CDCl3
76.788 CDCl3
36.519
33.596
22.544
Me
Ph
f1 (ppm)

2c

(7-Methyl-3-methyleneoct-6-en-1-yl)benzene (2d)

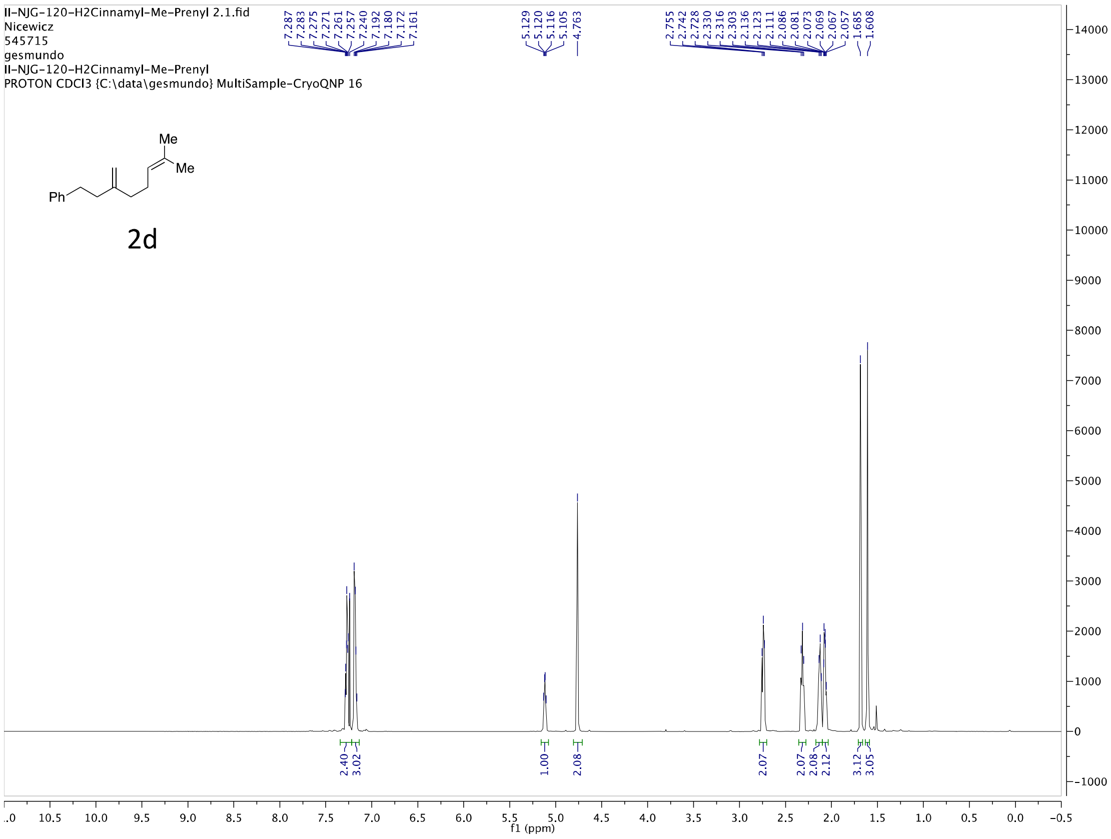

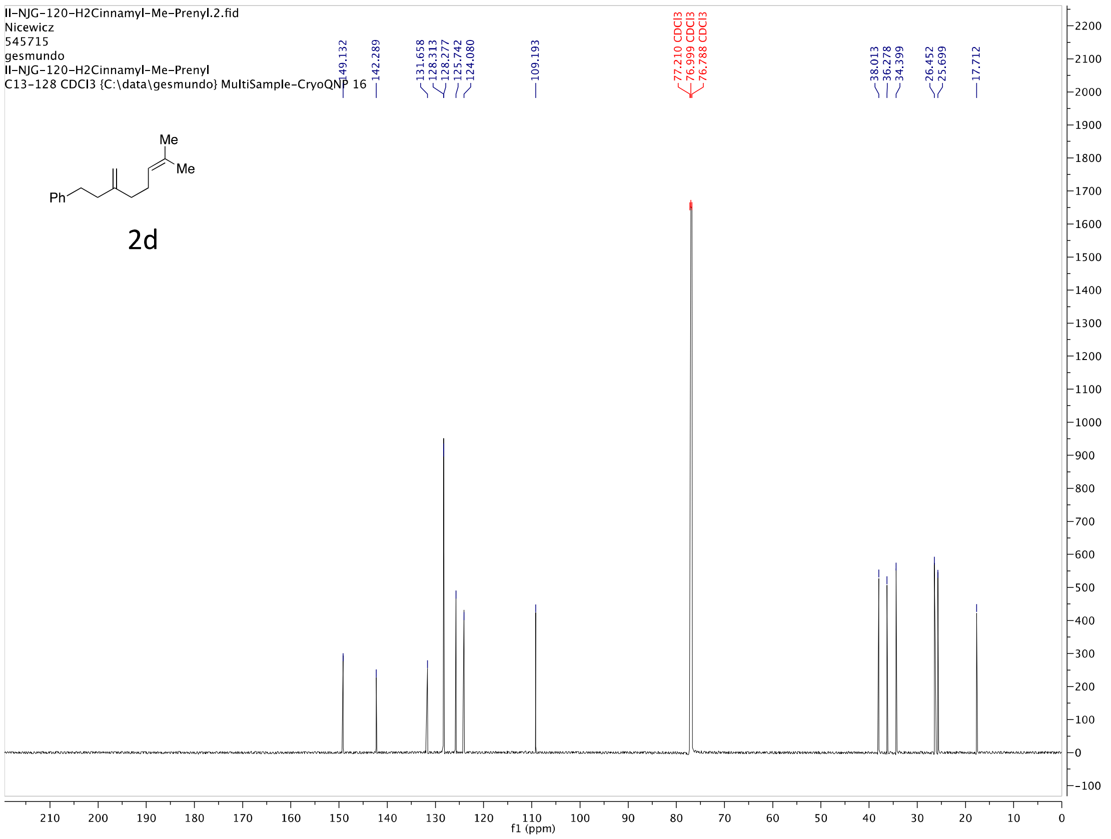
II-NJG-120-H2Cinnamyl-Me-Prenyl.2.fid
Nicewicz
545715
gesmundo
II-NJG-120-H2Cinnamyl-Me-Prenyl
C13-128 CDCl3 {C:\data\gesmundo} MultiSample-CryoQNP 16
149.132
142.289
131.658
128.313
128.277
125.742
124.080
109.193
77.210 CDCl3
76.999 CDCl3
76.788 CDCl3
38.013
36.278
34.399
26.452
25.699
17.712
Me
Me
Ph
2d
f1 (ppm)

(6-Methylhepta-1,5-dien-2-yl)benzene (2e)

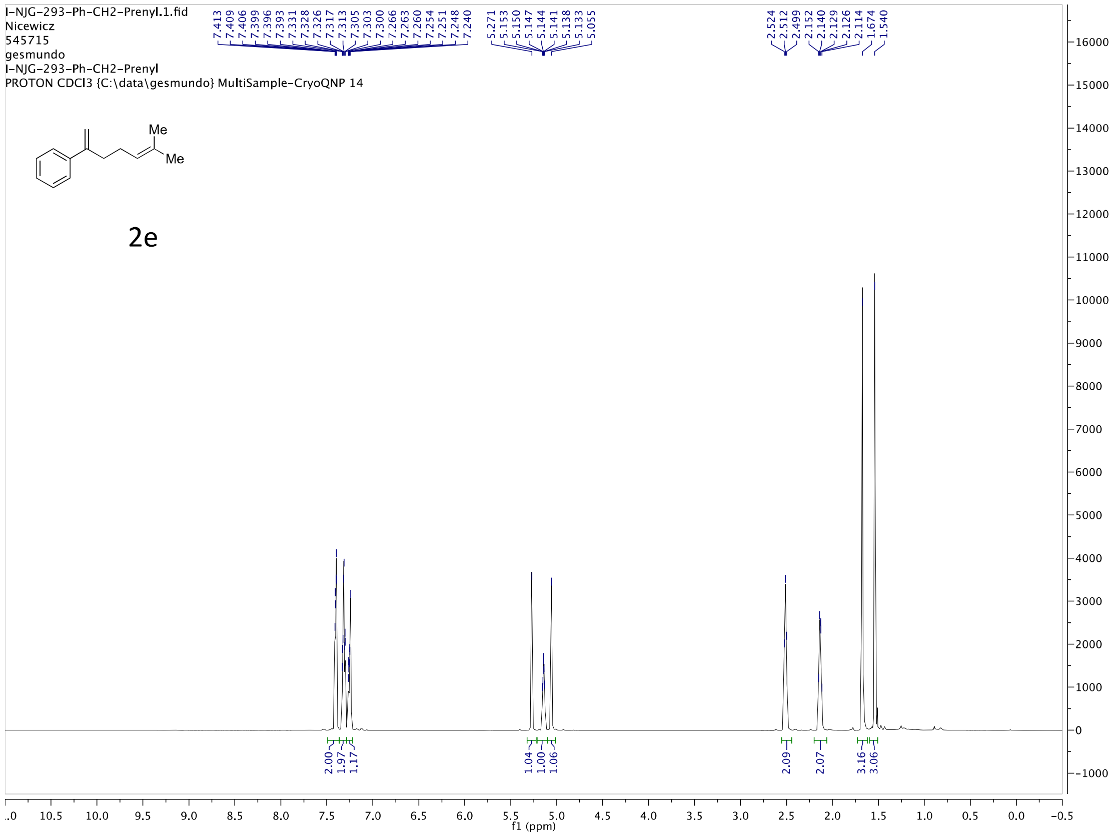

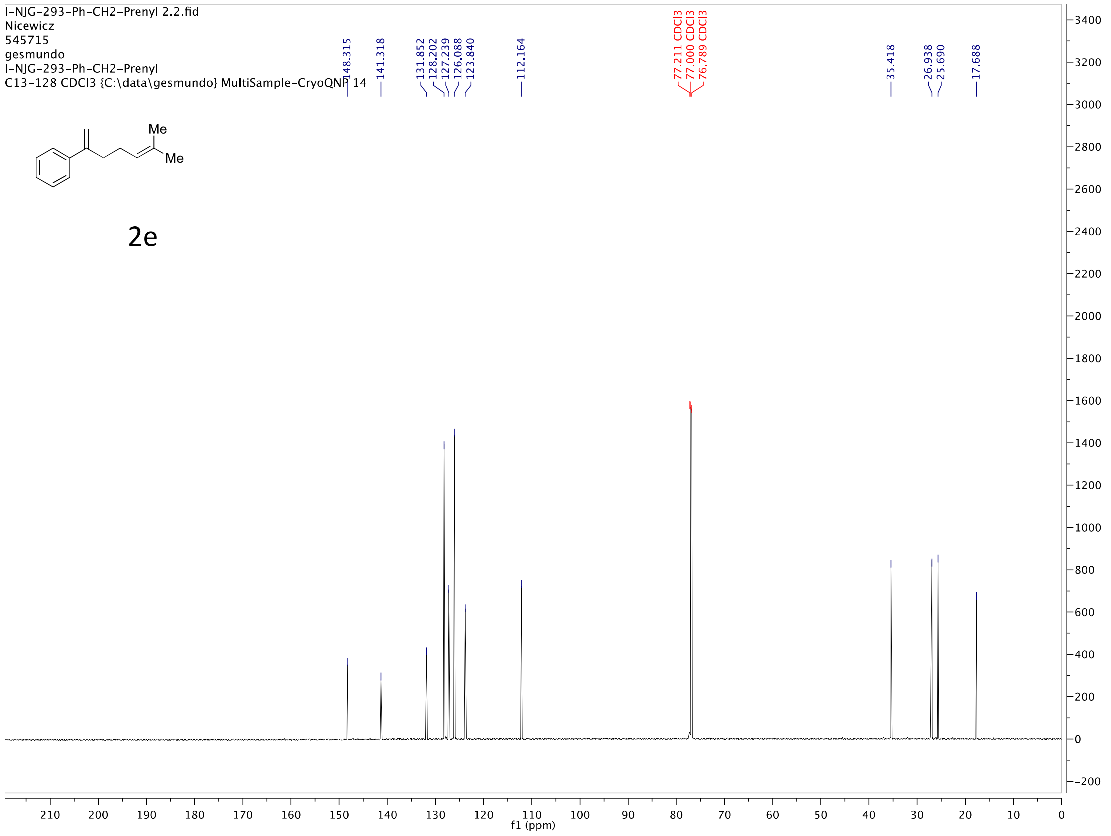
I-NJG-293-Ph-CH2-Prenyl 2.2.fid
Nicewicz
545715
gesmundo
I-NJG-293-Ph-CH2-Prenyl
C13-128 CDCl3 {C:\data\gesmundo} MultiSample-CryoQNP 14
Me
Me
2e
148.315
141.318
131.852
128.202
127.239
126.088
123.840
112.164
77.211 CDCl3
77.000 CDCl3
76.789 CDCl3
35.418
26.938
25.690
17.688
f1 (ppm)

1-Methoxy-4-(6-methylhepta-1,5-dien-2-yl)benzene (2f)

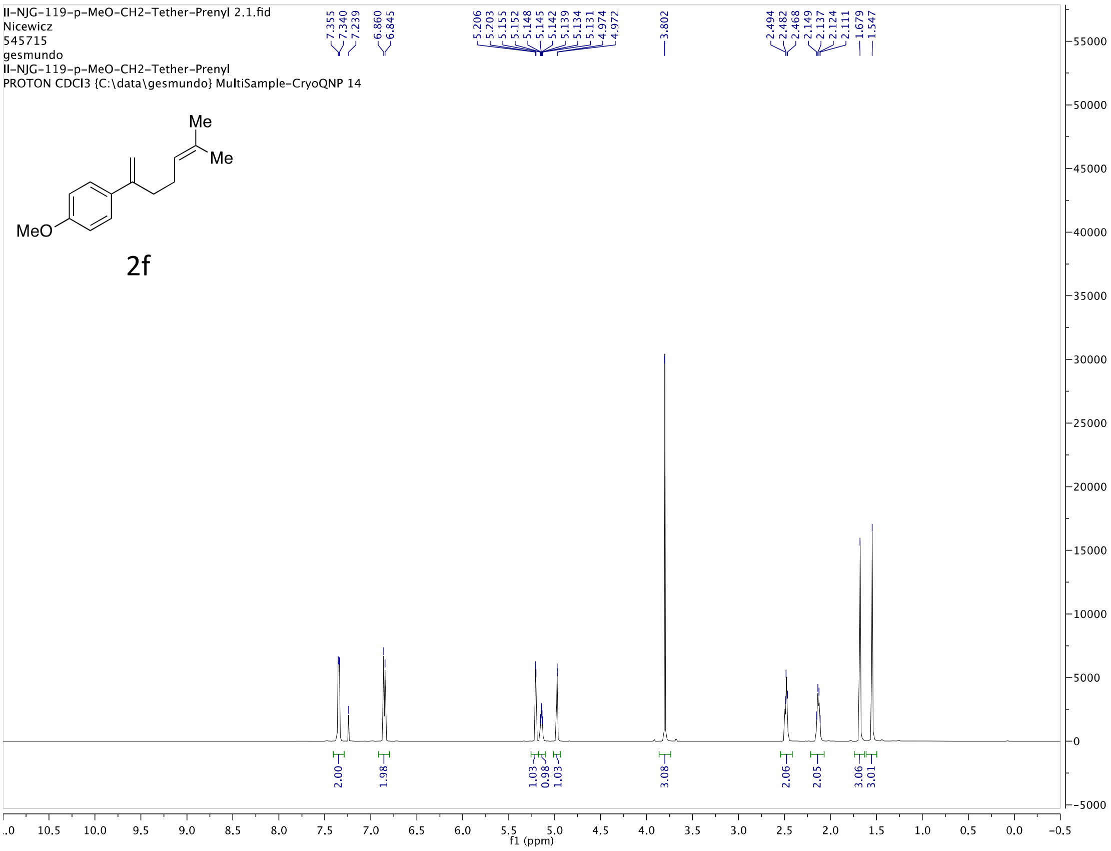

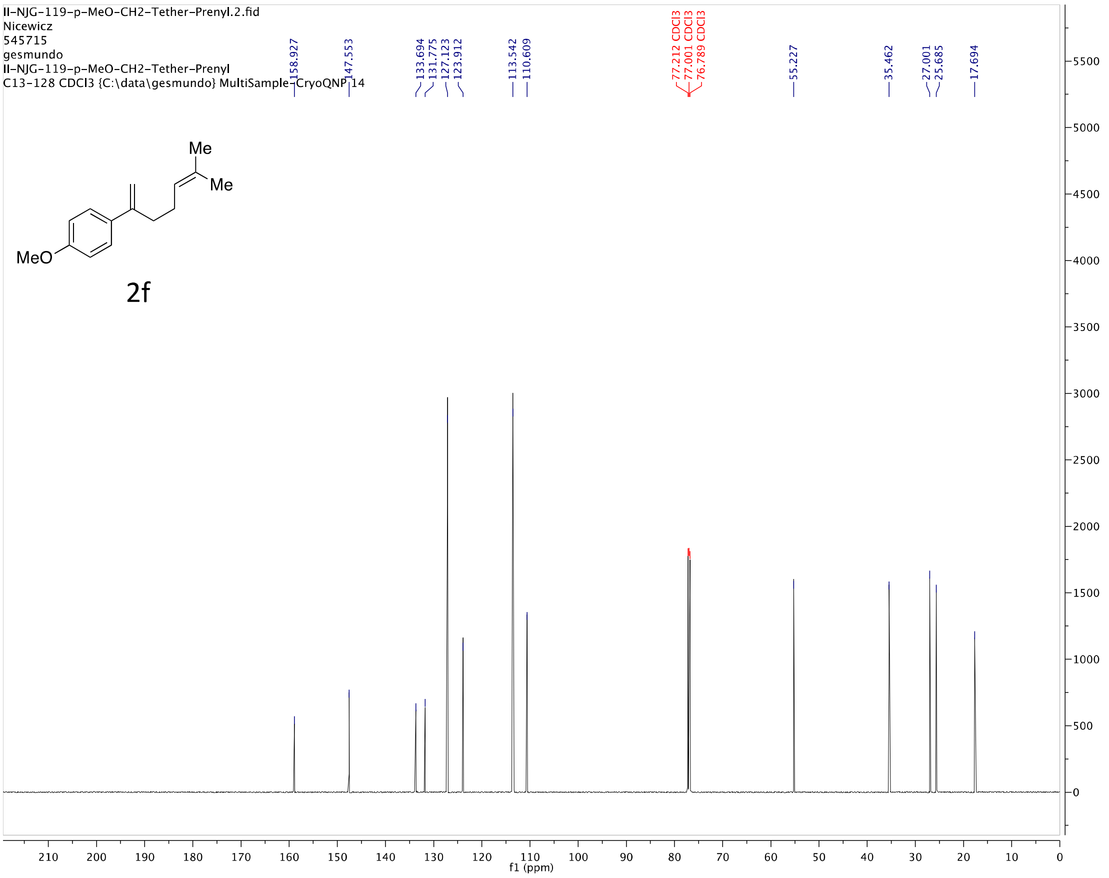
II-NJG-119-p-MeO-CH2-Tether-Prenyl.2.fid
Nicewicz
545715
gesmundo
II-NJG-119-p-MeO-CH2-Tether-Prenyl
C13-128 CDCl3 {C:\data\gesmundo} MultiSample-CryoQNP 14
158.927
147.553
133.694
131.775
127.123
123.912
113.542
110.609
77.212 CDCl3
77.001 CDCl3
76.789 CDCl3
55.227
35.462
27.001
25.685
17.694
Me
Me
MeO
2f
f1 (ppm)

2,4-Dimethoxy-1-(6-methylhepta-1,5-dien-2-yl)benzene (2g)

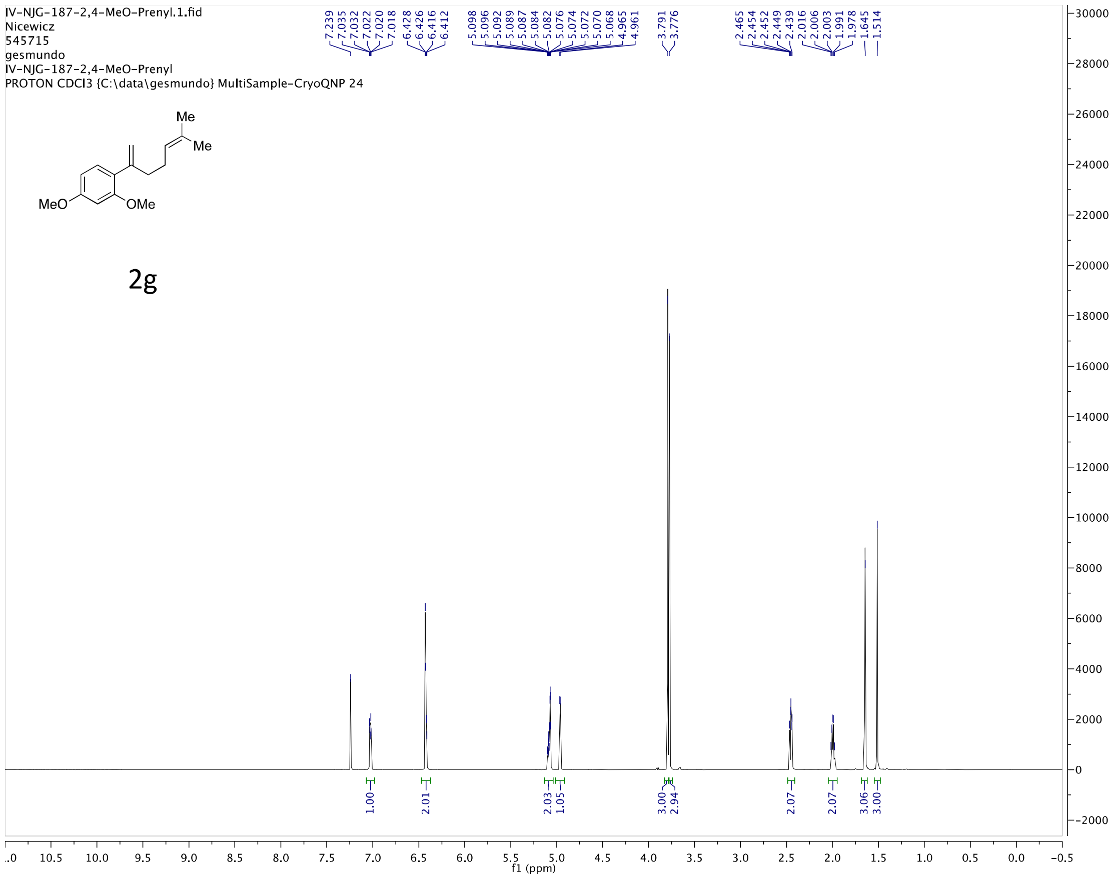

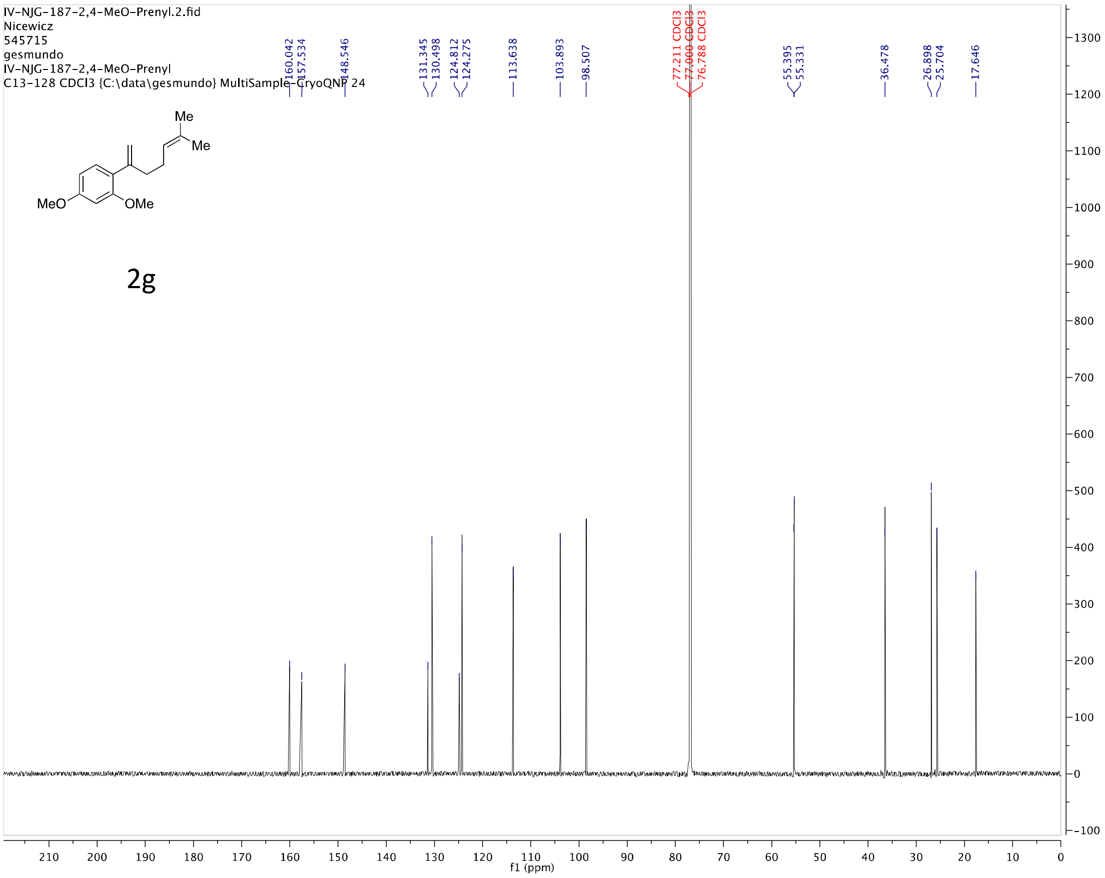
IV-NJG-187-2,4-MeO-Prenyl.2.fid
Nicewicz
545715
gesmundo
IV-NJG-187-2,4-MeO-Prenyl
C13-128 CDCl3 {C:\data\gesmundo} MultiSample-CryoQNP 24
160.042
157.534
148.546
131.345
130.498
124.812
124.275
113.638
103.893
98.507
77.211 CDCl3
77.000 CDCl3
76.788 CDCl3
55.395
55.331
36.478
26.898
25.704
17.646
Me
Me
MeO
OMe
2g
f1 (ppm)

1,2-Dimethoxy-4-(6-methylhepta-1,5-dien-2-yl)benzene (2h)

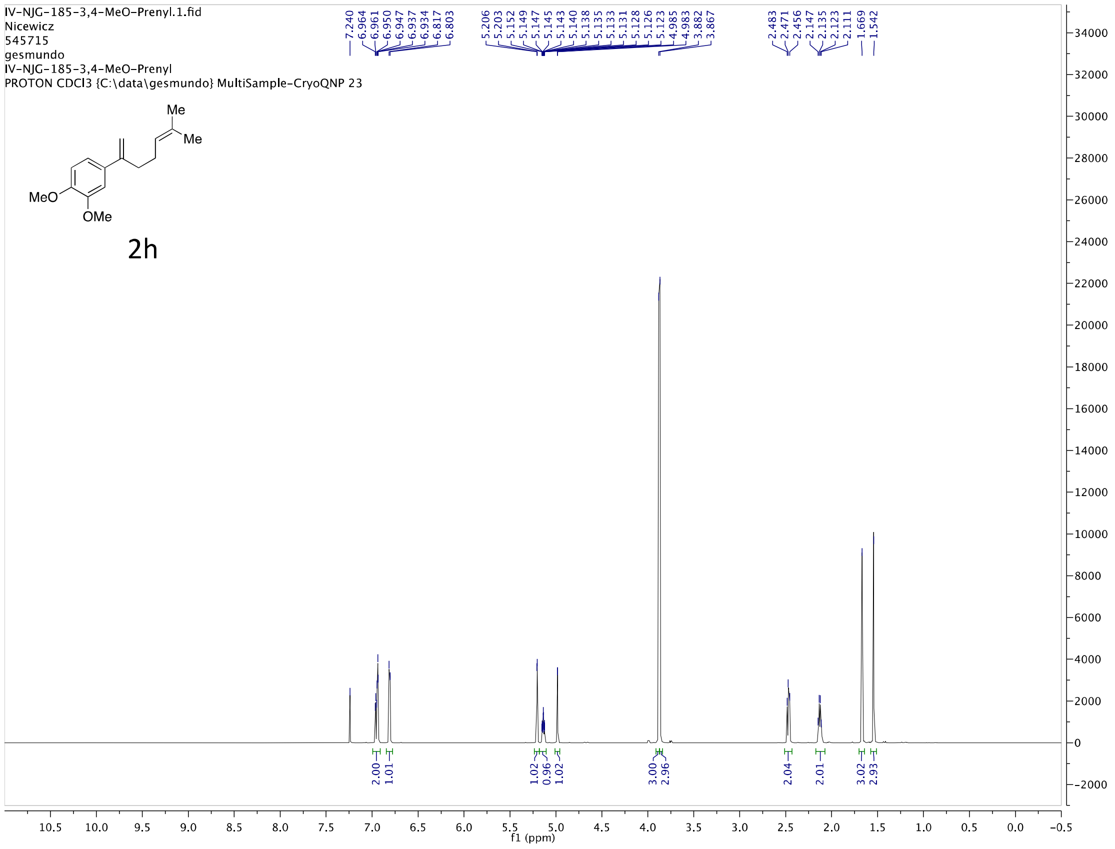

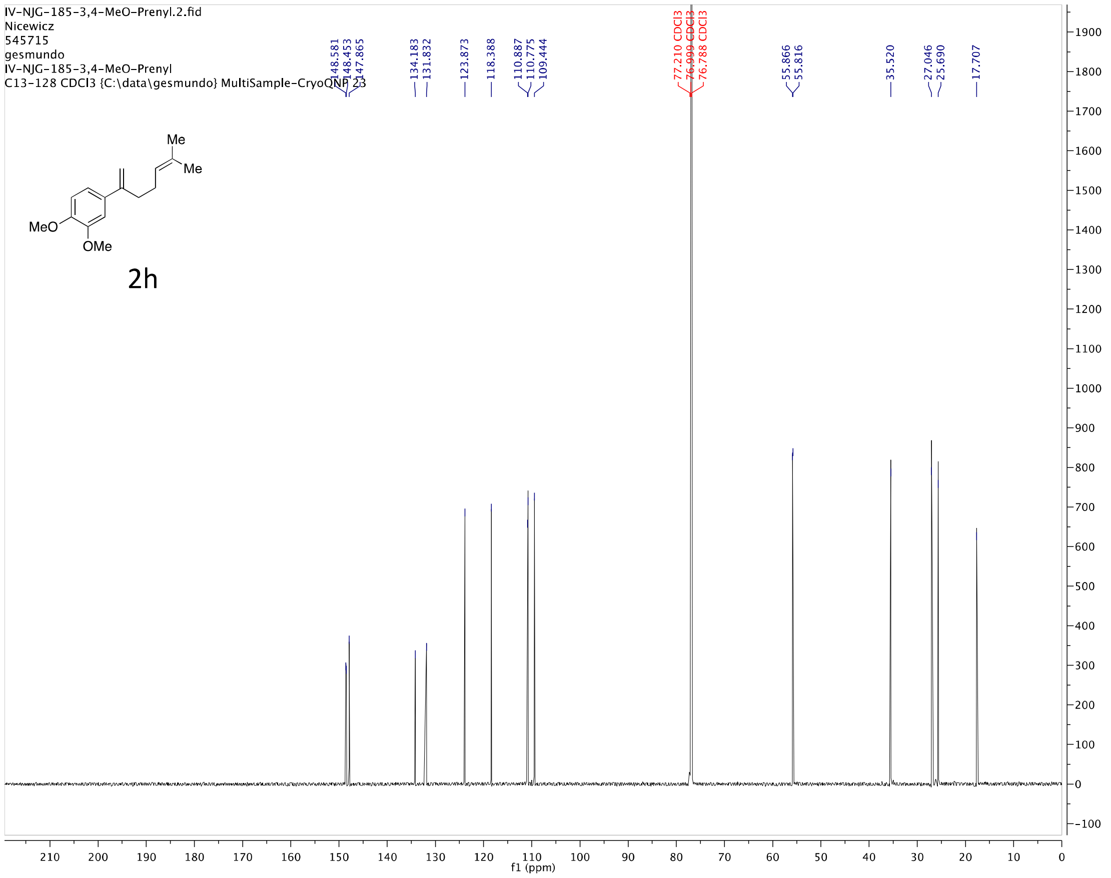
IV-NJG-185-3,4-MeO-Prenyl.2.fid
Nicewicz
545715
gesmundo
IV-NJG-185-3,4-MeO-Prenyl
C13-128 CDCl3 {C:\data\gesmundo} MultiSample-CryoQNP 23
148.581
148.453
147.865
134.183
131.832
123.873
118.388
110.887
110.775
109.444
77.210 CDCl3
76.999 CDCl3
76.788 CDCl3
55.866
55.816
35.520
27.046
25.690
17.707
Me
Me
MeO
OMe
2h
f1 (ppm)

1-Methoxy-2-(6-methylhepta-1,5-dien-2-yl)benzene (2i)

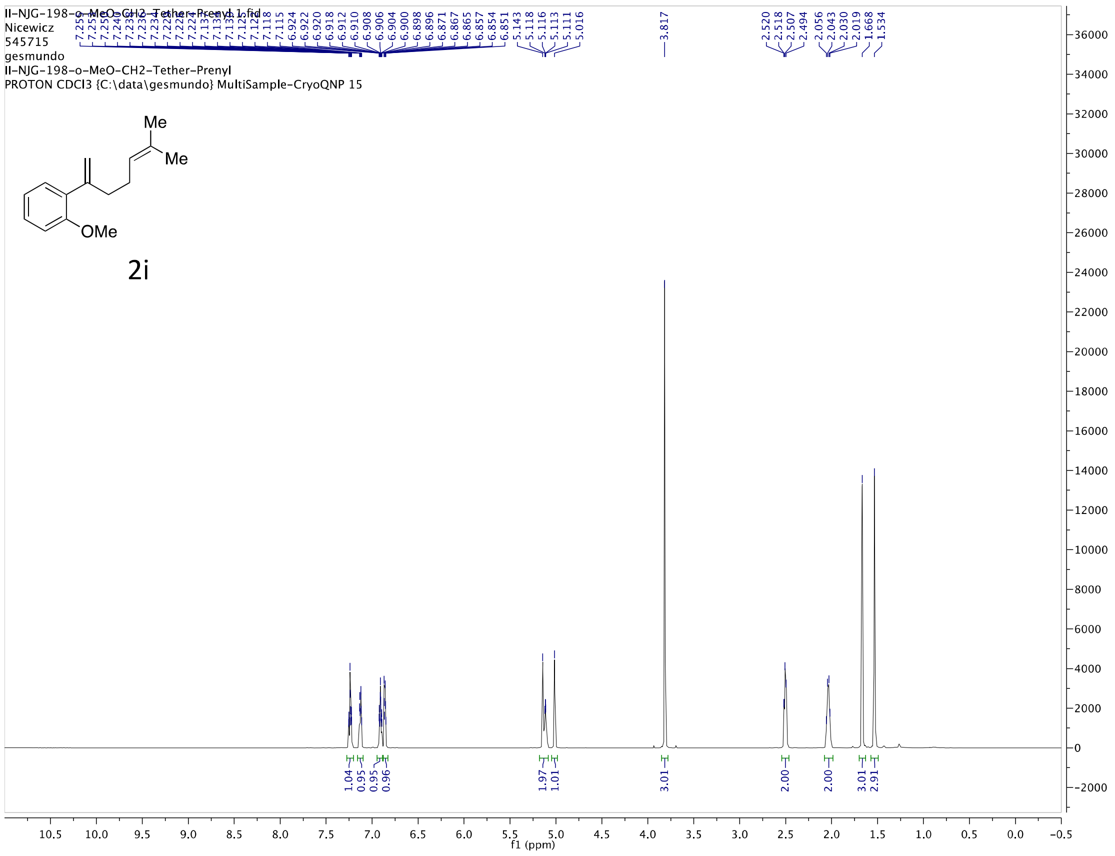

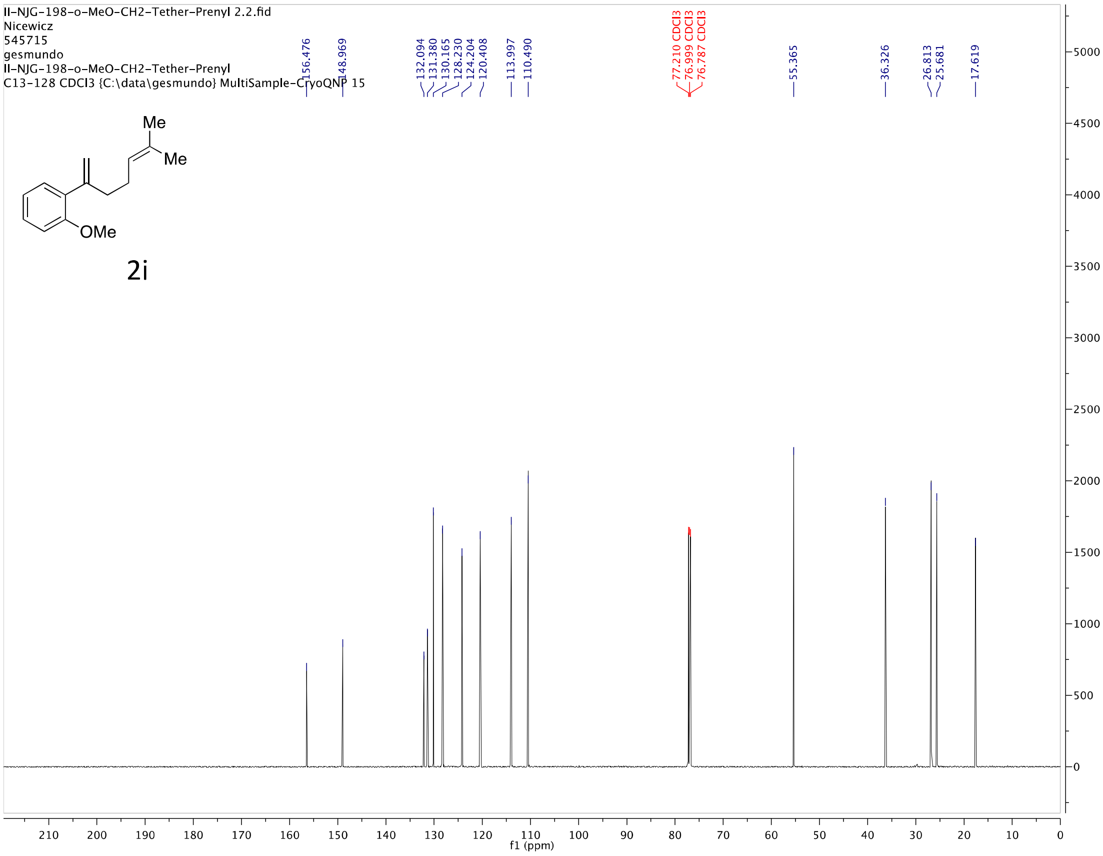
II-NJG-198-o-MeO-CH2-Tether-Prenyl 2.2.fid
Nicewicz
545715
gesmundo
II-NJG-198-o-MeO-CH2-Tether-Prenyl
C13-128 CDCl3 {C:\data\gesmundo} MultiSample-CryoQNP 15
156.476
148.969
132.094
131.380
130.165
128.230
124.204
120.408
113.997
110.490
77.210 CDCl3
76.999 CDCl3
76.787 CDCl3
55.365
36.326
26.813
25.681
17.619
Me
Me
OMe
2i
f1 (ppm)

1-Methyl-4-(6-methylhepta-1,5-dien-2-yl)benzene (2j)

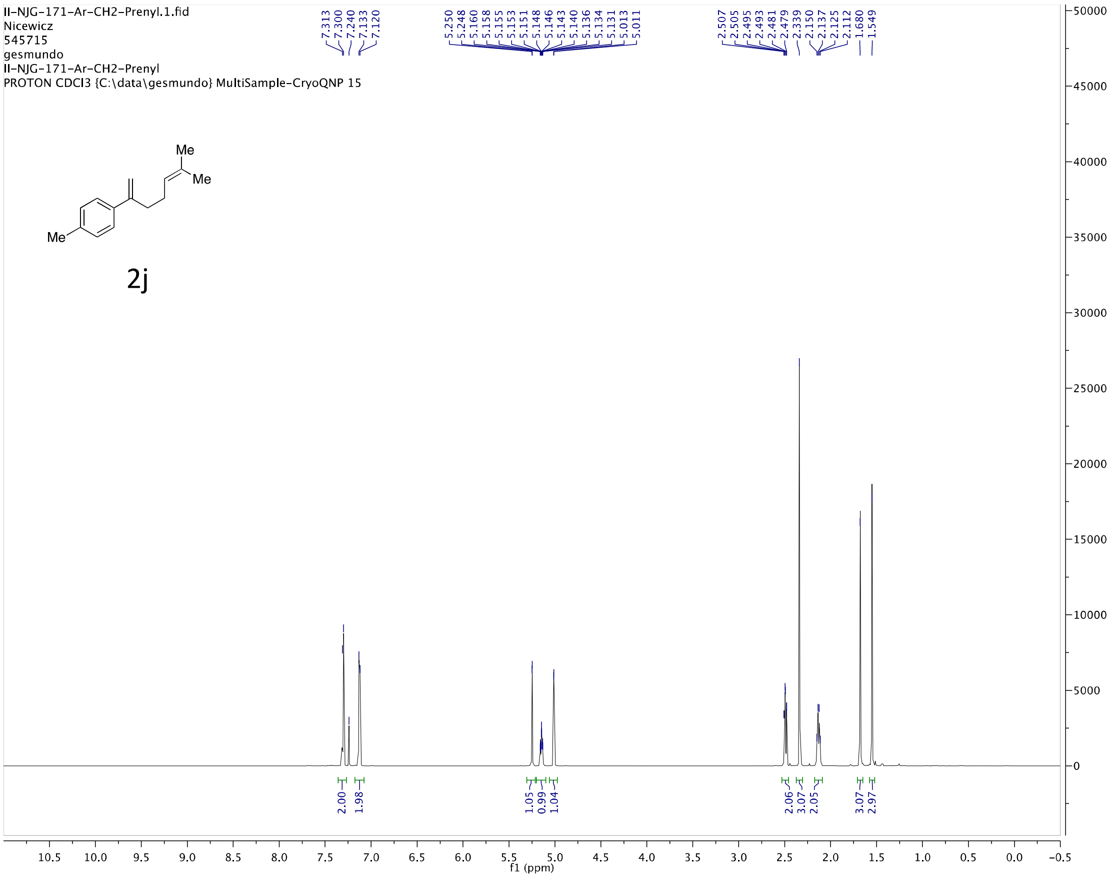

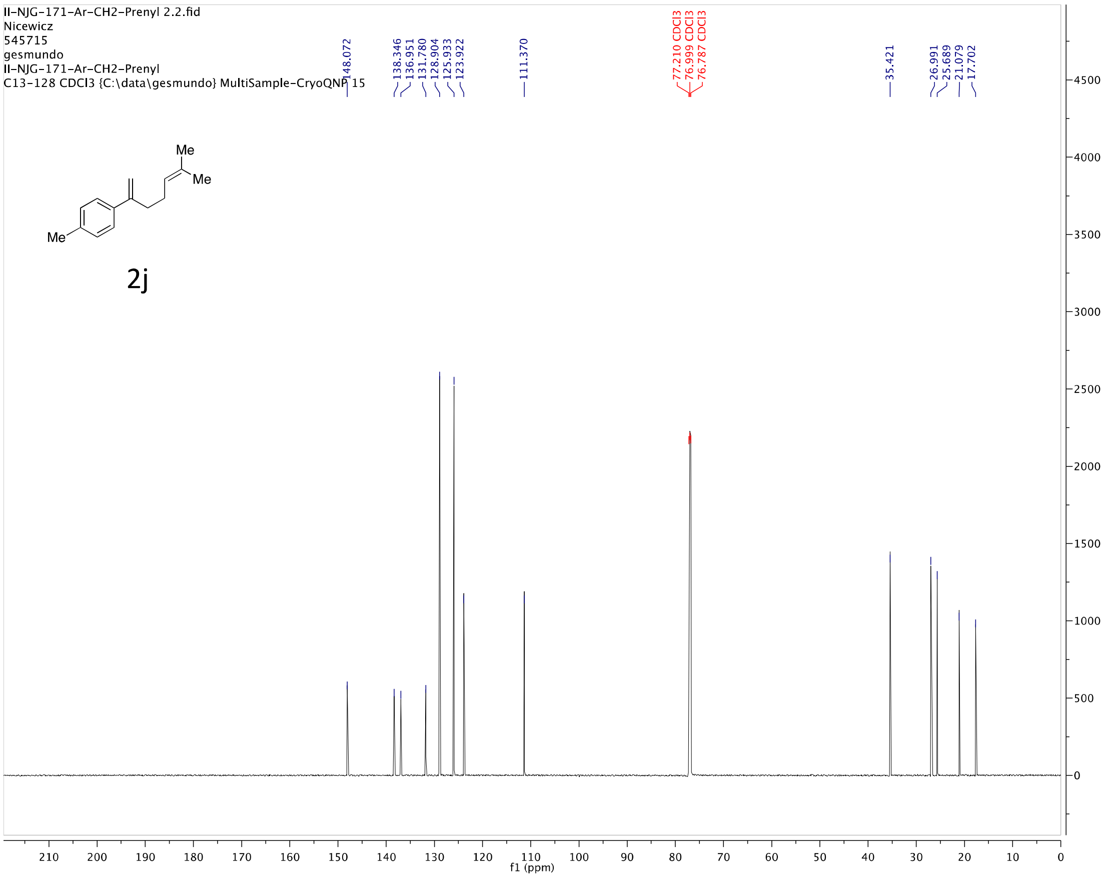
II-NJG-171-Ar-CH2-Prenyl 2.2.fid
Nicewicz
545715
gesmundo
II-NJG-171-Ar-CH2-Prenyl
C13-128 CDCl3 {C:\data\gesmundo} MultiSample-CryoQNP 15
148.072
138.346
136.951
131.780
128.904
125.933
123.922
111.370
77.210 CDCl3
76.999 CDCl3
76.787 CDCl3
35.421
26.991
25.689
21.079
17.702
Me
Me
Me
2j
f1 (ppm)

1-Chloro-4-(6-methylhepta-1,5-dien-2-yl)benzene (2k)

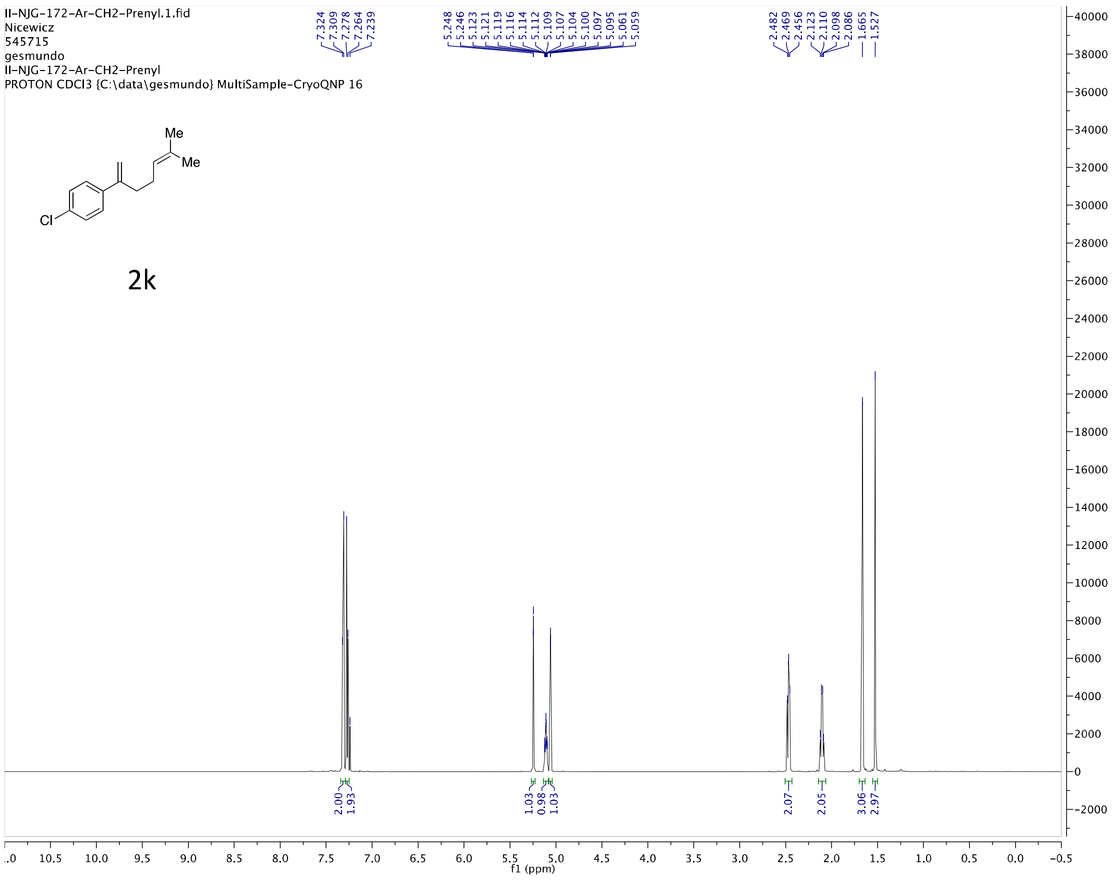

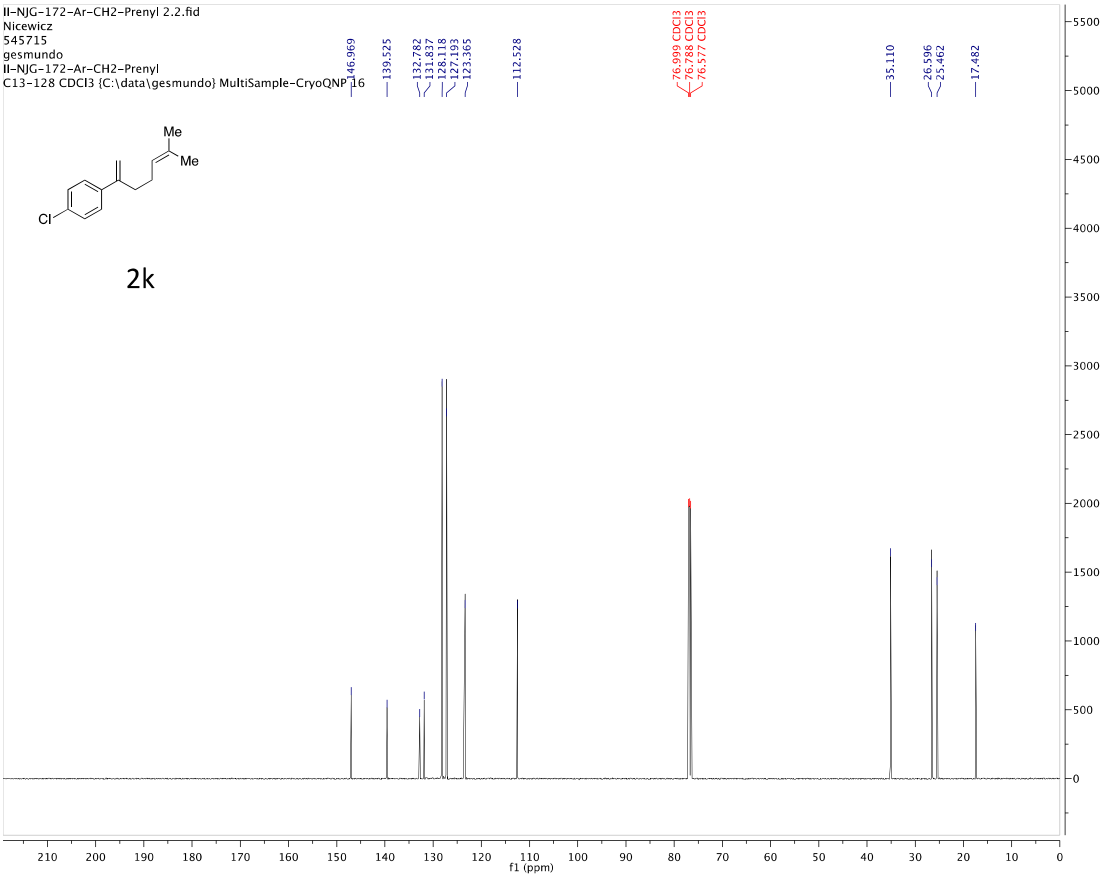
II-NJG-172-Ar-CH2-Prenyl 2.2.fid
Nicewicz
545715
gesmundo
II-NJG-172-Ar-CH2-Prenyl
C13-128 CDCl3 {C:\data\gesmundo} MultiSample-CryoQNP 16
146.969
139.525
132.782
131.837
128.118
127.193
123.365
112.528
76.999 CDCl3
76.788 CDCl3
76.577 CDCl3
35.110
26.596
25.462
17.482
Me
Me
Cl
2k
f1 (ppm)

2-(6-Methylhepta-1,5-dien-2-yl)furan (2l)

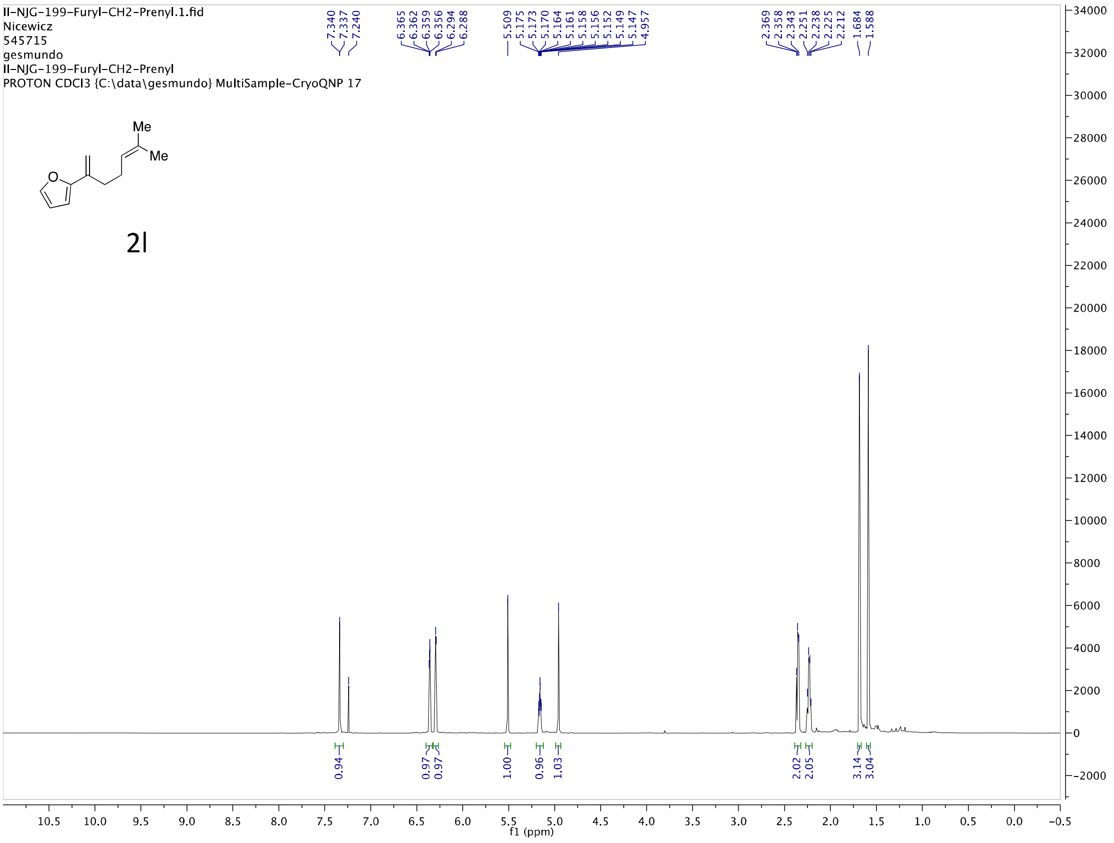

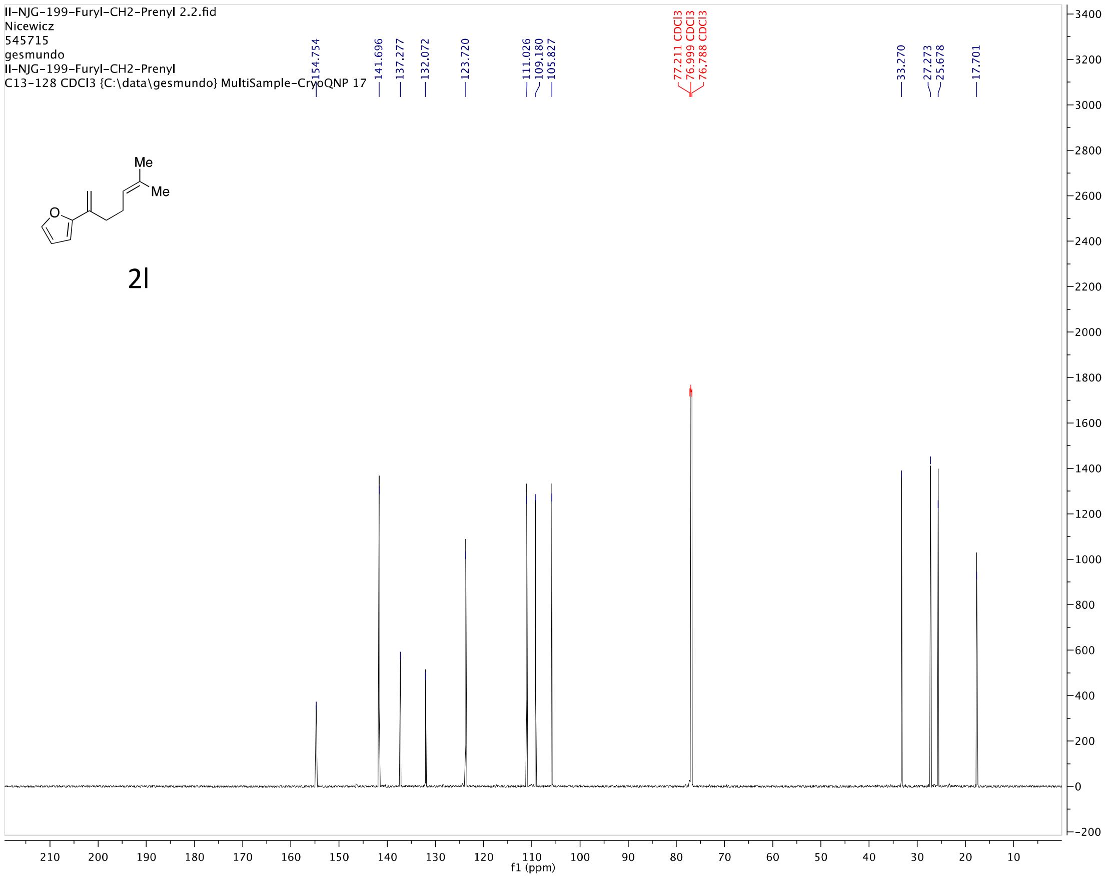
II-NJG-199-Furyl-CH2-Prenyl 2.2.fid
Nicewicz
545715
gesmundo
II-NJG-199-Furyl-CH2-Prenyl
C13-128 CDCl3 {C:\data\gesmundo} MultiSample-CryoQNP 17
154.754
141.696
137.277
132.072
123.720
111.026
109.180
105.827
77.211 CDCl3
76.999 CDCl3
76.788 CDCl3
33.270
27.273
25.678
17.701
Me
Me
O
2l
f1 (ppm)

(*E*)-1-(3,3-Dimethyl-6-phenylhexa-1,5-dien-2-yl)-4-methoxybenzene (2m)

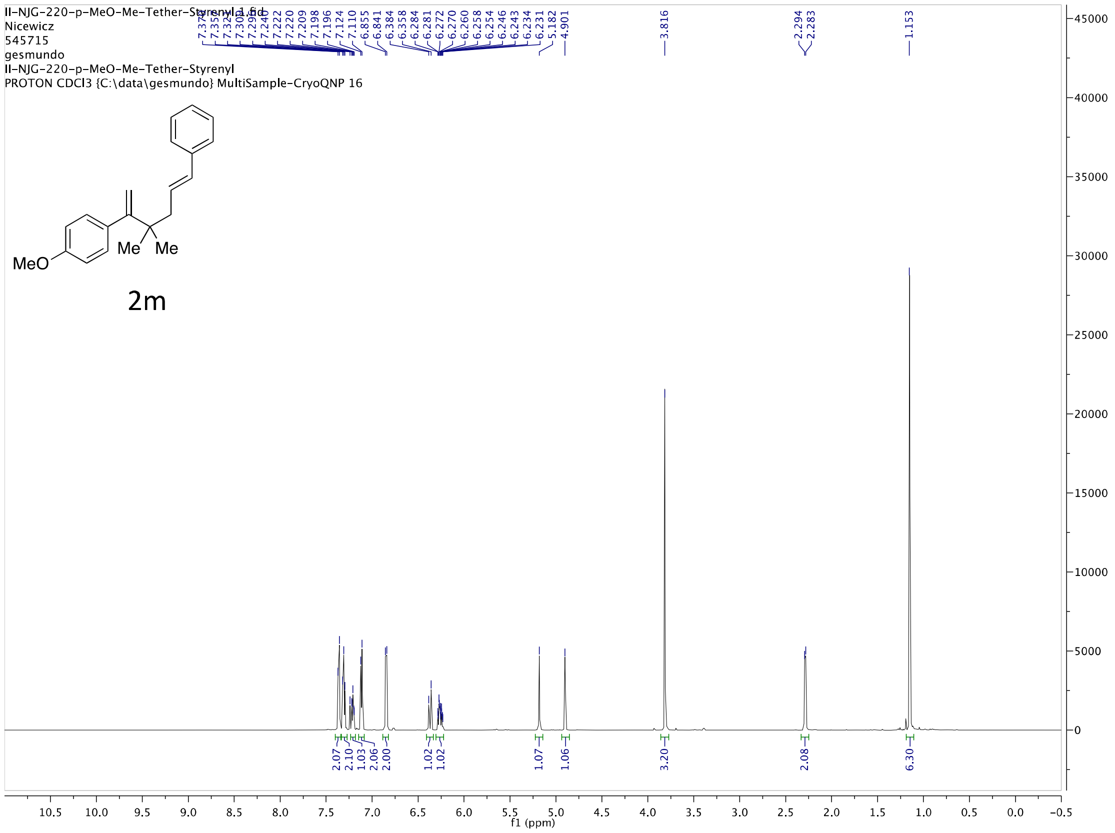

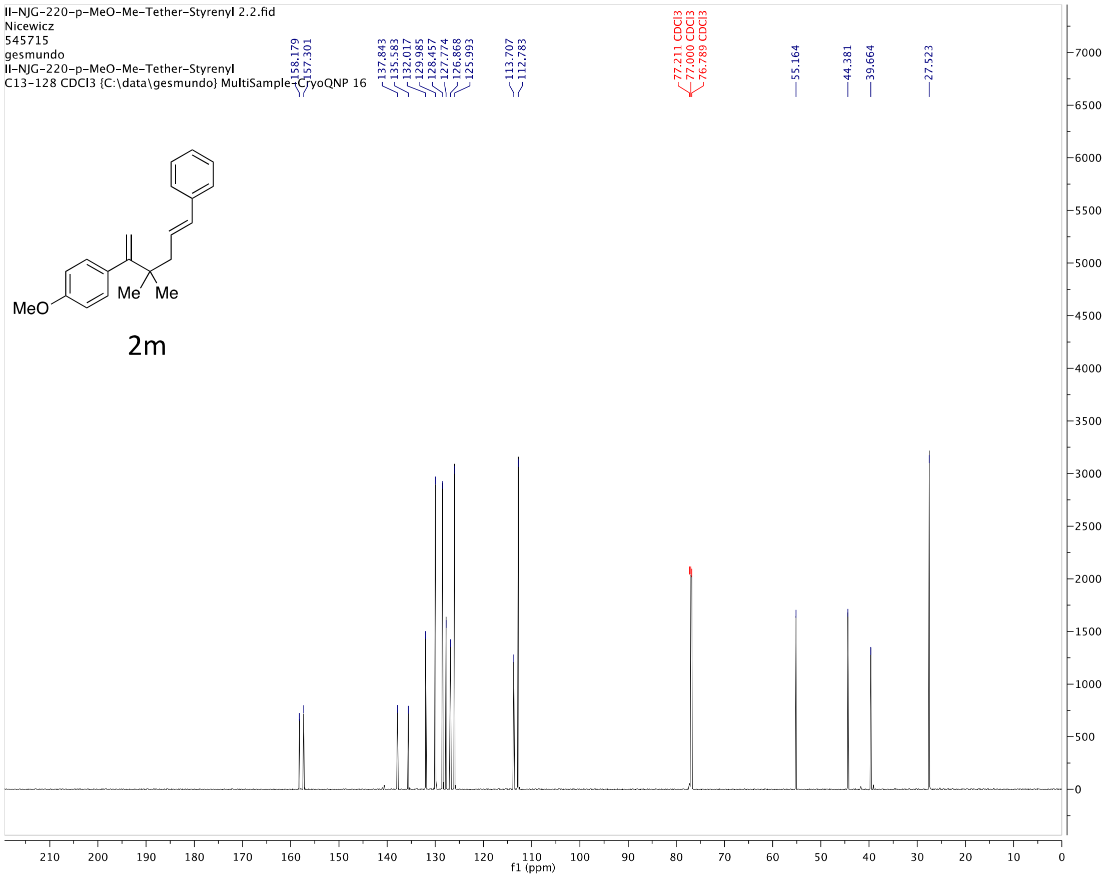

II-NJG-220-p-MeO-Me-Tether-Styrenyl 2.2.fid
Nicewicz
545715
gesmundo
II-NJG-220-p-MeO-Me-Tether-Styrenyl
C13-128 CDCl3 {C:\data\gesmundo} MultiSample-CryoQNP 16
158.179
157.301
137.843
135.583
132.017
129.985
128.457
127.774
126.868
125.993
113.707
112.783
77.211 CDCl3
77.000 CDCl3
76.789 CDCl3
55.164
44.381
39.664
27.523
MeO
Me
Me
2m
f1 (ppm)

1-(3,3-Dimethyl-4-(2,6,6-trimethylcyclohex-1-en-1-yl)but-1-en-2-yl)-4-methoxybenzene (2n)

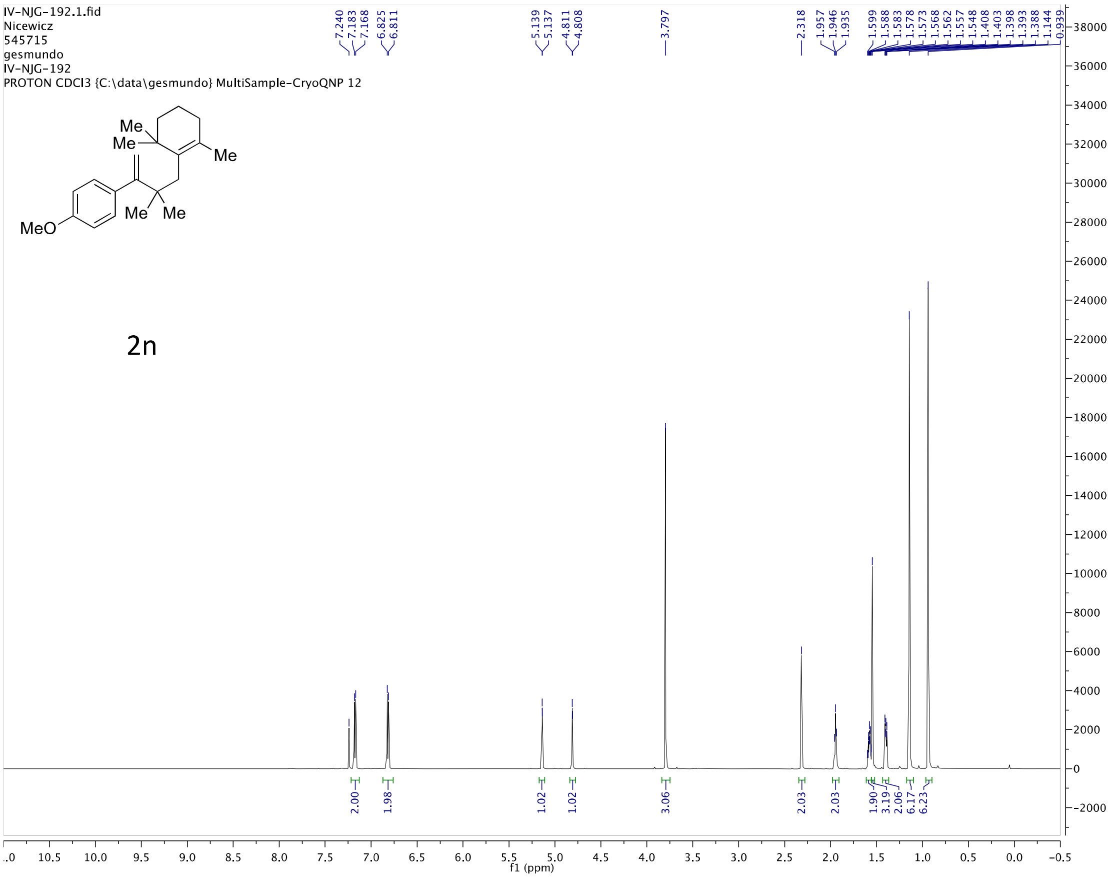

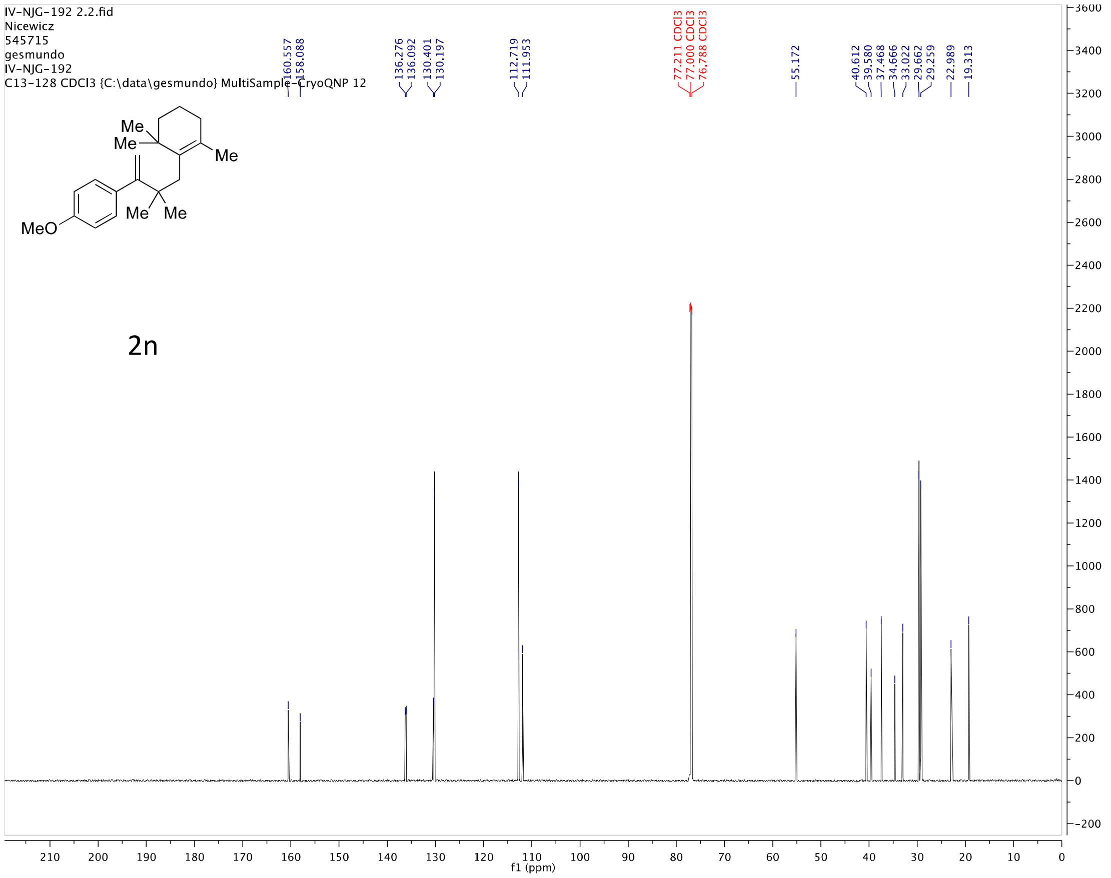
IV-NJG-192 2.2.fid
Nicewicz
545715
gesmundo
IV-NJG-192
C13-128 CDCl3 {C:\data\gesmundo} MultiSample-CryoQNP 12
160.557
158.088
136.276
136.092
130.401
130.197
112.719
111.953
77.211 CDCl3
77.000 CDCl3
76.788 CDCl3
55.172
40.612
39.580
37.468
34.666
33.022
29.662
29.259
22.989
19.313
Me
Me
Me
Me
Me
MeO
2n
f1 (ppm)

(*E*)-1-(3,3-Dimethylhepta-1,5-dien-2-yl)-4-methoxybenzene (2o)

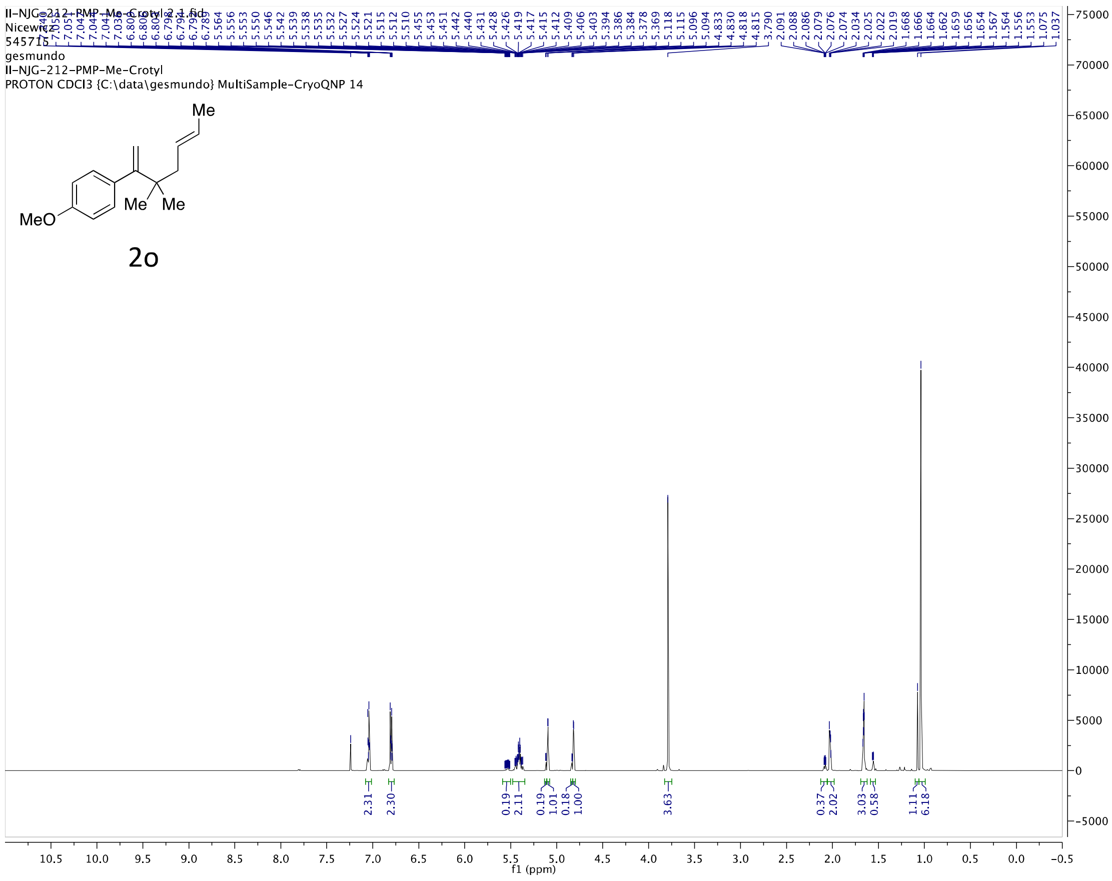

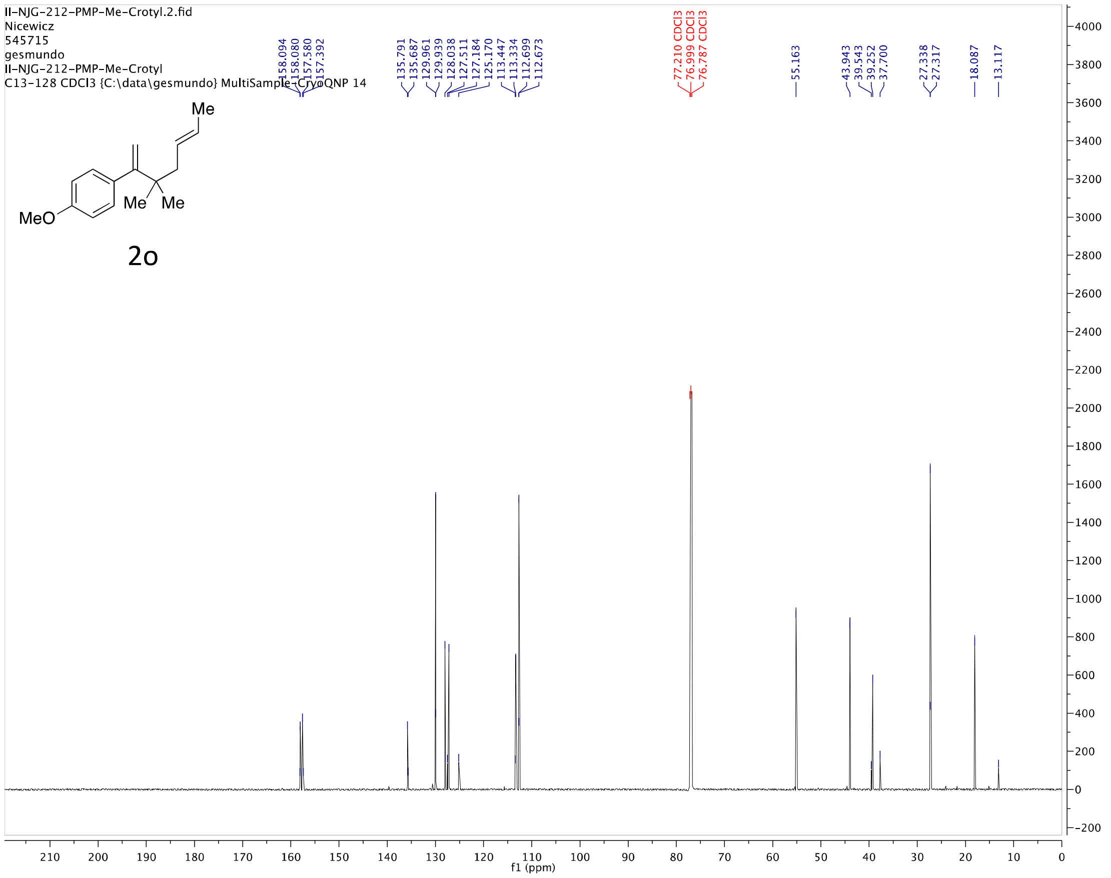
II-NJG-212-PMP-Me-Crotyl.2.fid
Nicewicz
545715
gesmundo
II-NJG-212-PMP-Me-Crotyl
C13-128 CDCl3 {C:\data\gesmundo} MultiSample-CryoQNP 14
158.094
158.080
157.580
157.392
135.791
135.687
129.961
129.939
128.038
127.511
127.184
125.170
113.447
113.334
112.699
112.673
77.210 CDCl3
76.999 CDCl3
76.787 CDCl3
55.163
43.943
39.543
39.252
37.700
27.338
27.317
18.087
13.117
Me
MeO
Me
Me
2o
f1 (ppm)

1-Methoxy-4-(3,3,5-trimethylhexa-1,5-dien-2-yl)benzene (2p)

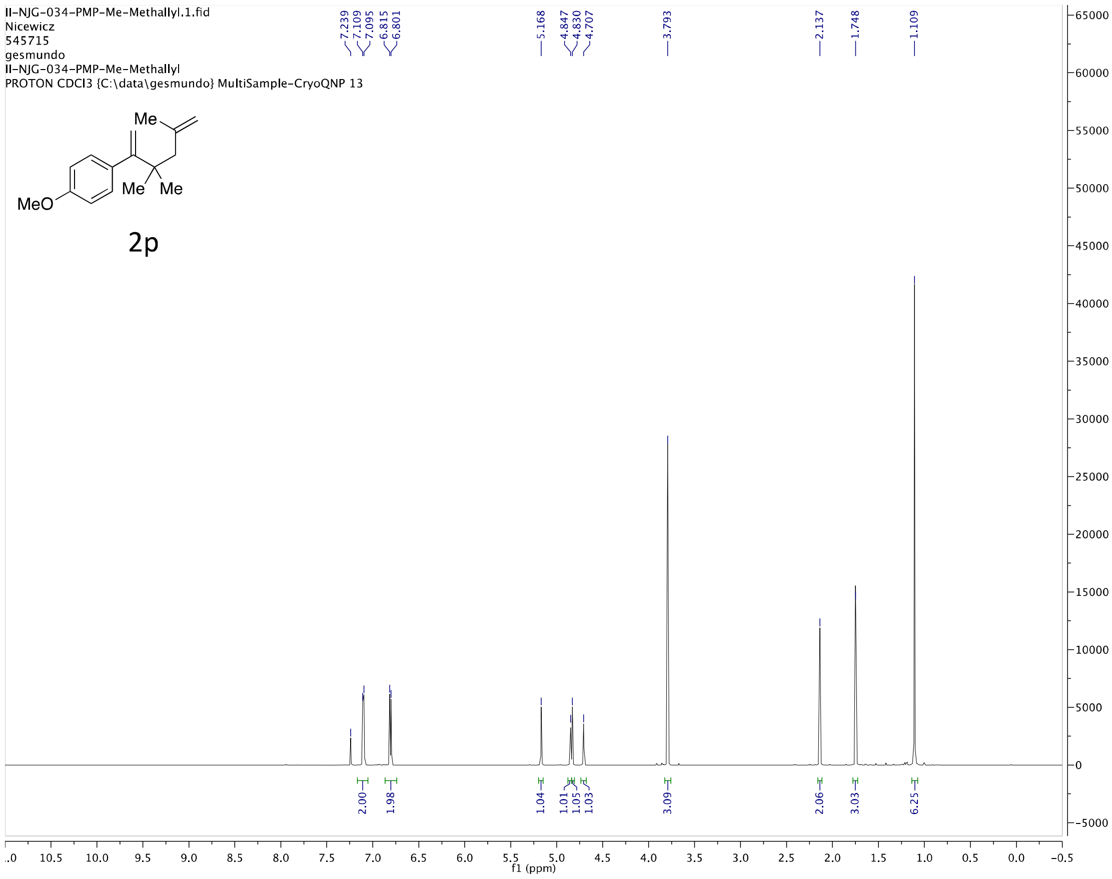

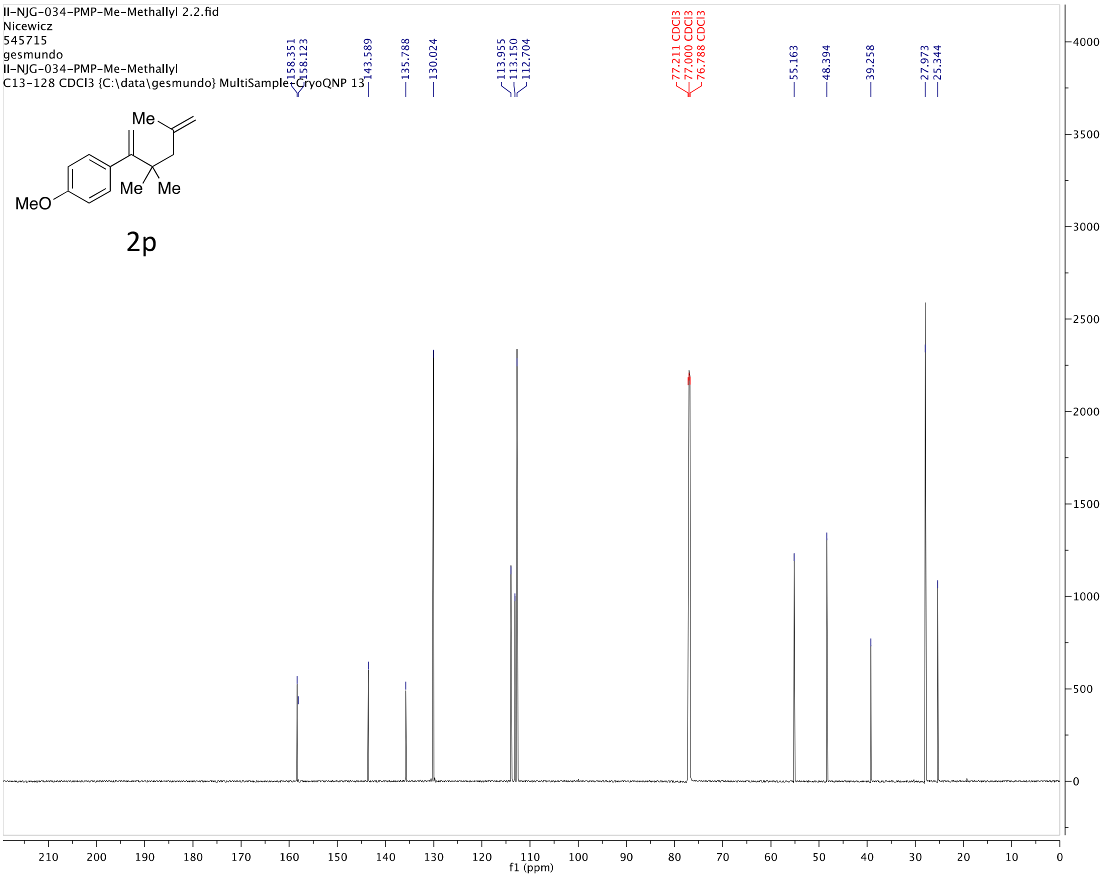
II-NJG-034-PMP-Me-Methallyl 2.2.fid
Nicewicz
545715
gesmundo
II-NJG-034-PMP-Me-Methallyl
C13-128 CDCl3 {C:\data\gesmundo} MultiSample-CryoQNP 13
158.351
158.123
143.589
135.788
130.024
113.955
113.150
112.704
77.211 CDCl3
77.000 CDCl3
76.788 CDCl3
55.163
48.394
39.258
27.973
25.344
Me
MeO
Me
Me
2p
f1 (ppm)

1-(3,3-Dimethylhexa-1,5-dien-2-yl)-4-methoxybenzene (2q)

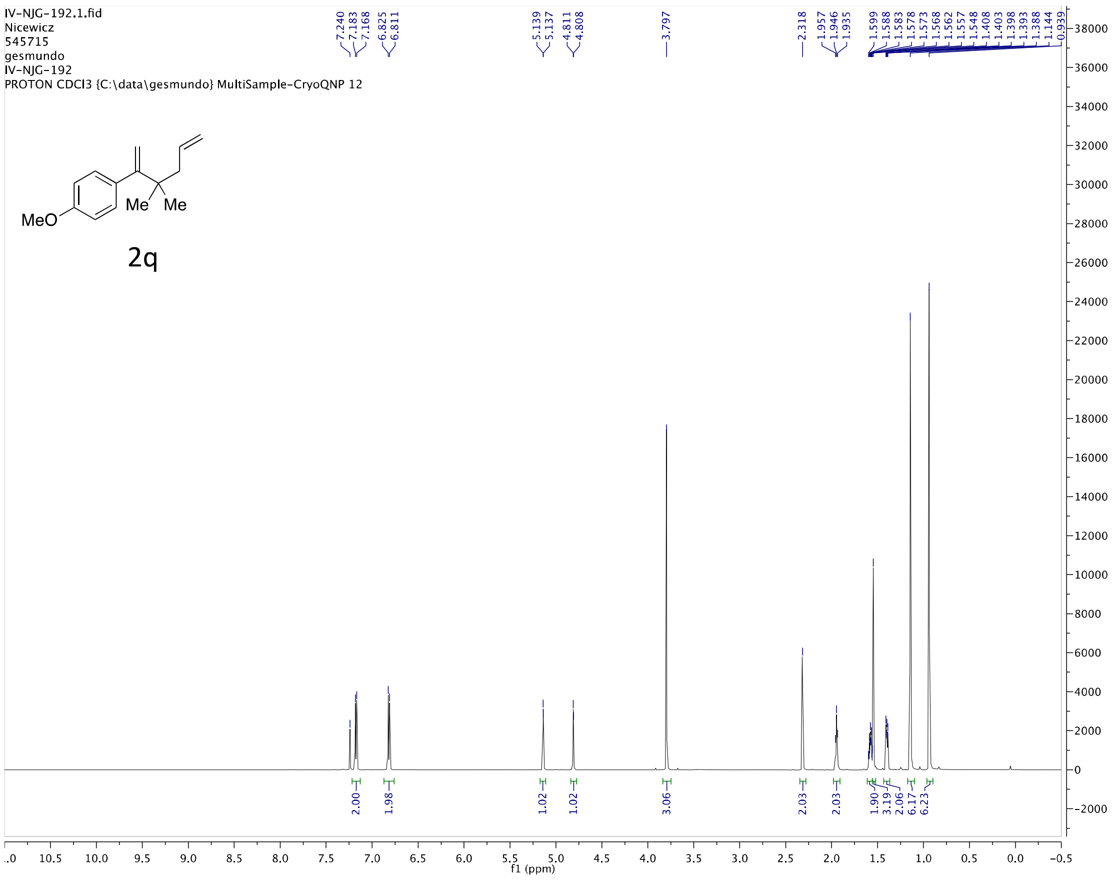

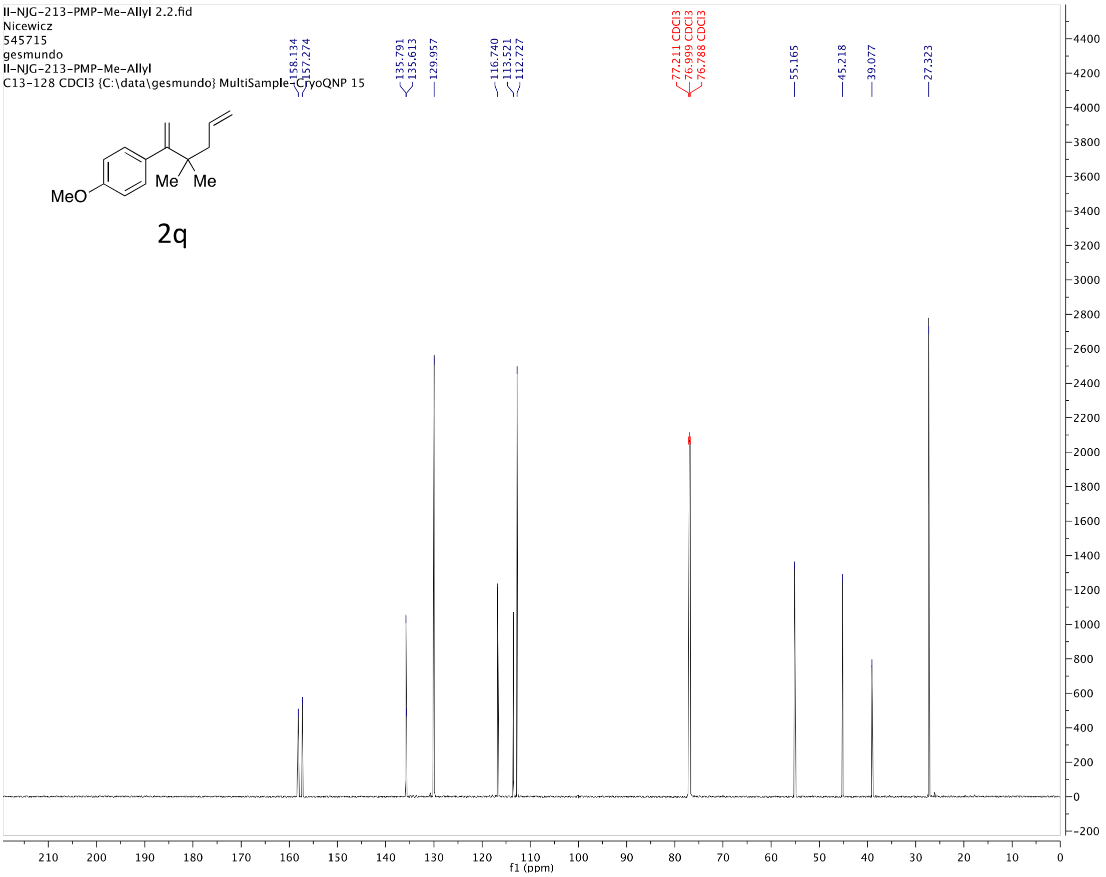
II-NJG-213-PMP-Me-Allyl 2.2.fid
Nicewicz
545715
gesmundo
II-NJG-213-PMP-Me-Allyl
C13-128 CDCl3 {C:\data\gesmundo} MultiSample-CryoQNP 15
158.134
157.274
135.791
135.613
129.957
116.740
113.521
112.727
77.211 CDCl3
76.999 CDCl3
76.788 CDCl3
55.165
45.218
39.077
27.323
MeO
Me
Me
2q
f1 (ppm)

(*Z*)-1-(Hepta-1,5-dien-2-yl)-4-methoxybenzene (2r)

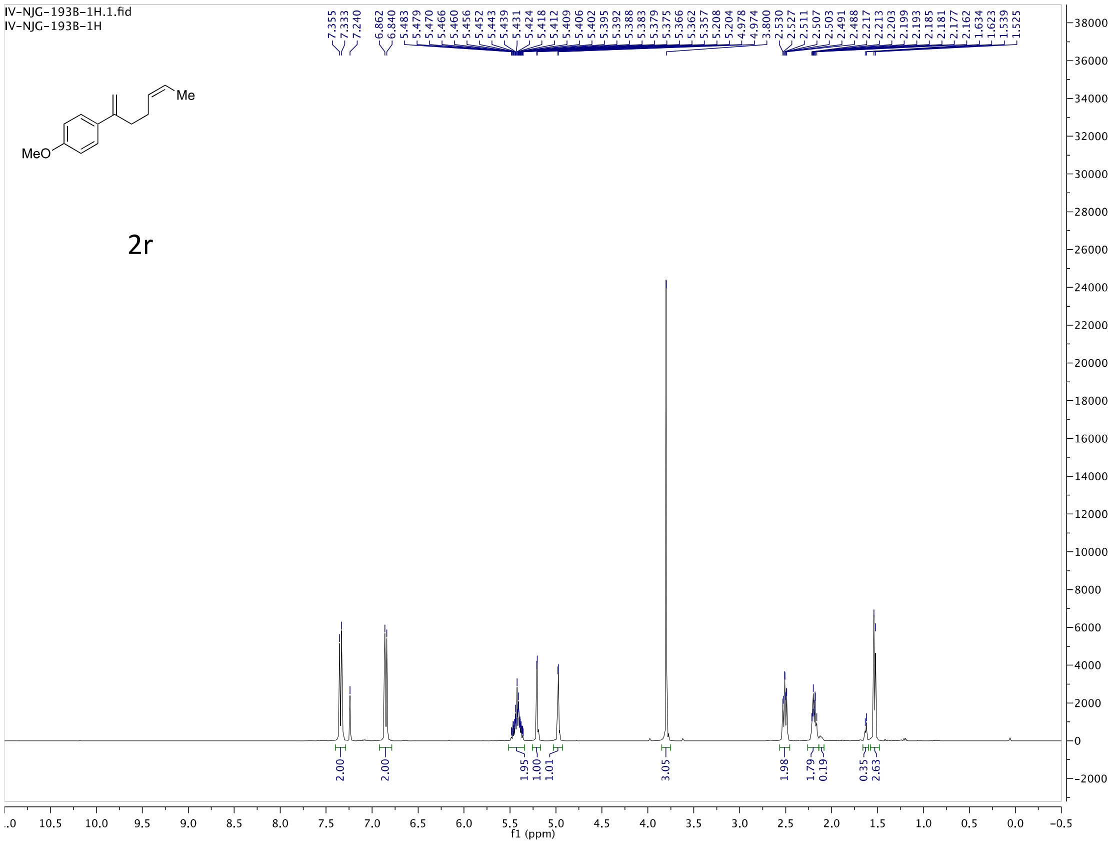

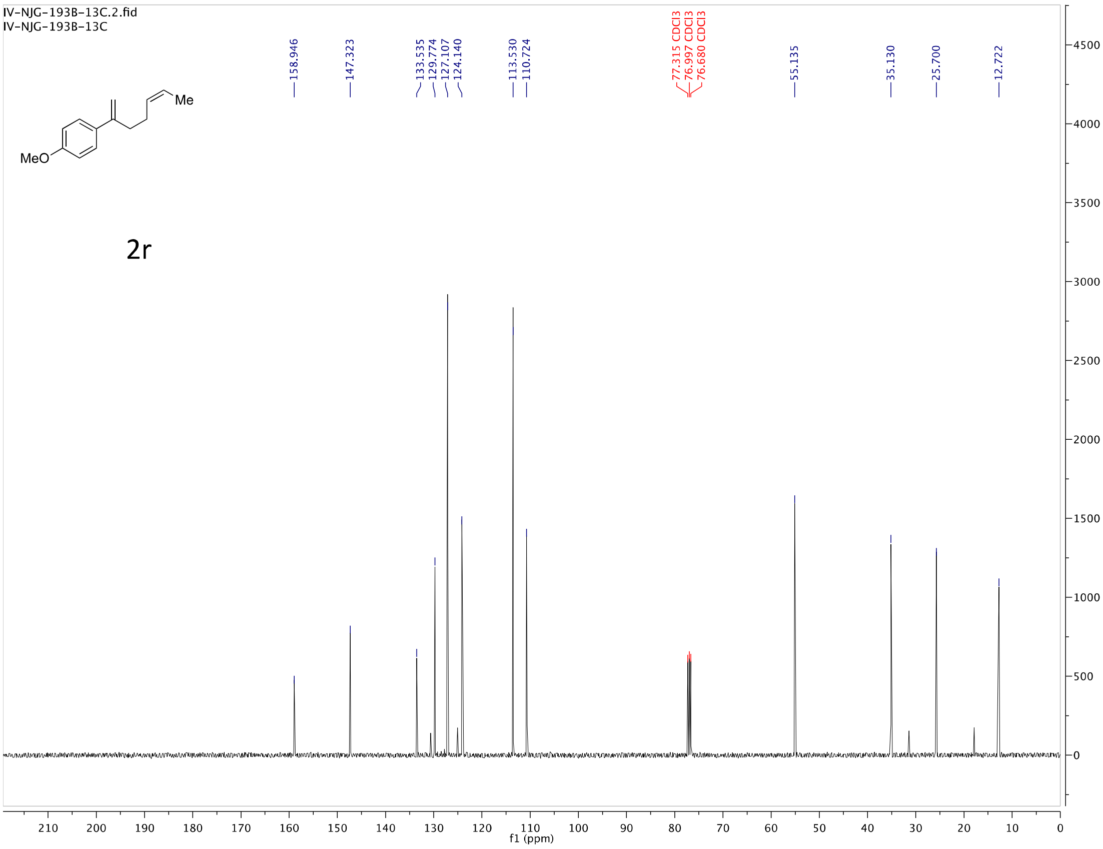
IV-NJG-193B-13C.2.fid
IV-NJG-193B-13C
158.946
147.323
133.535
129.774
127.107
124.140
113.530
110.724
77.315 CDCl3
76.997 CDCl3
76.680 CDCl3
55.135
35.130
25.700
12.722
Me
MeO
f1 (ppm)

2r

1-(Hexa-1,5-dien-2-yl)-4-methoxybenzene (2s)

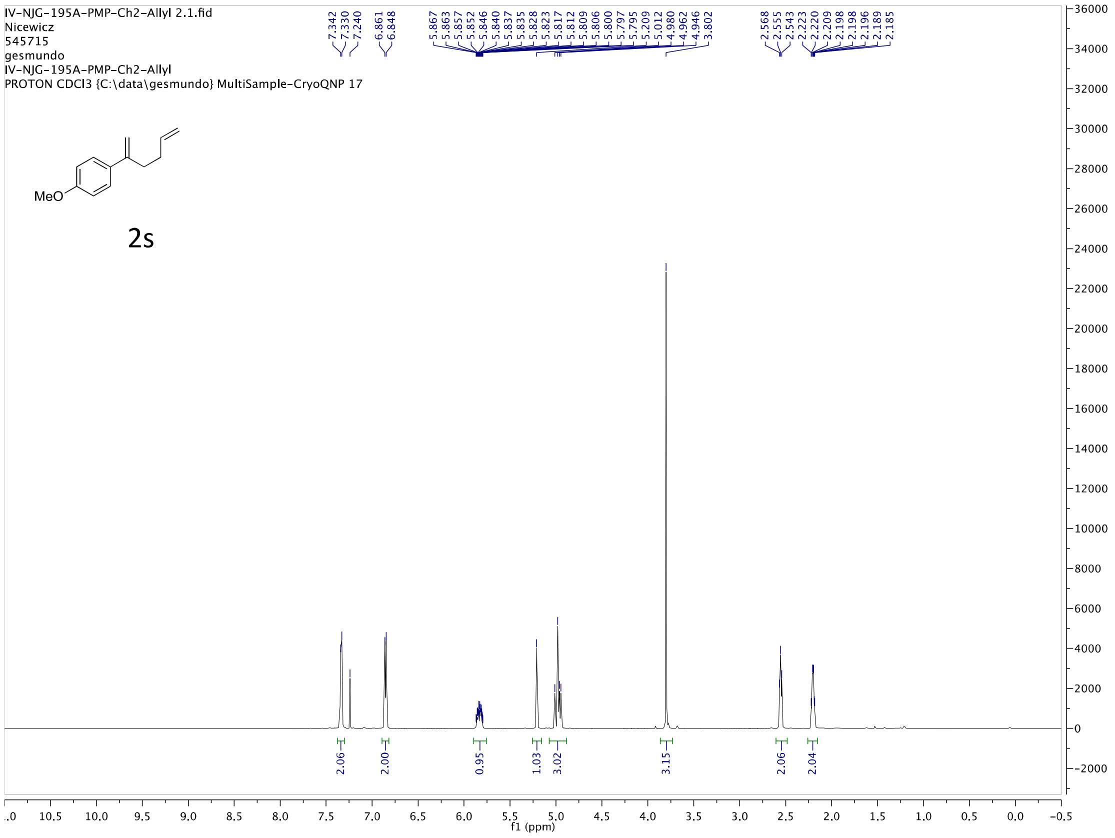

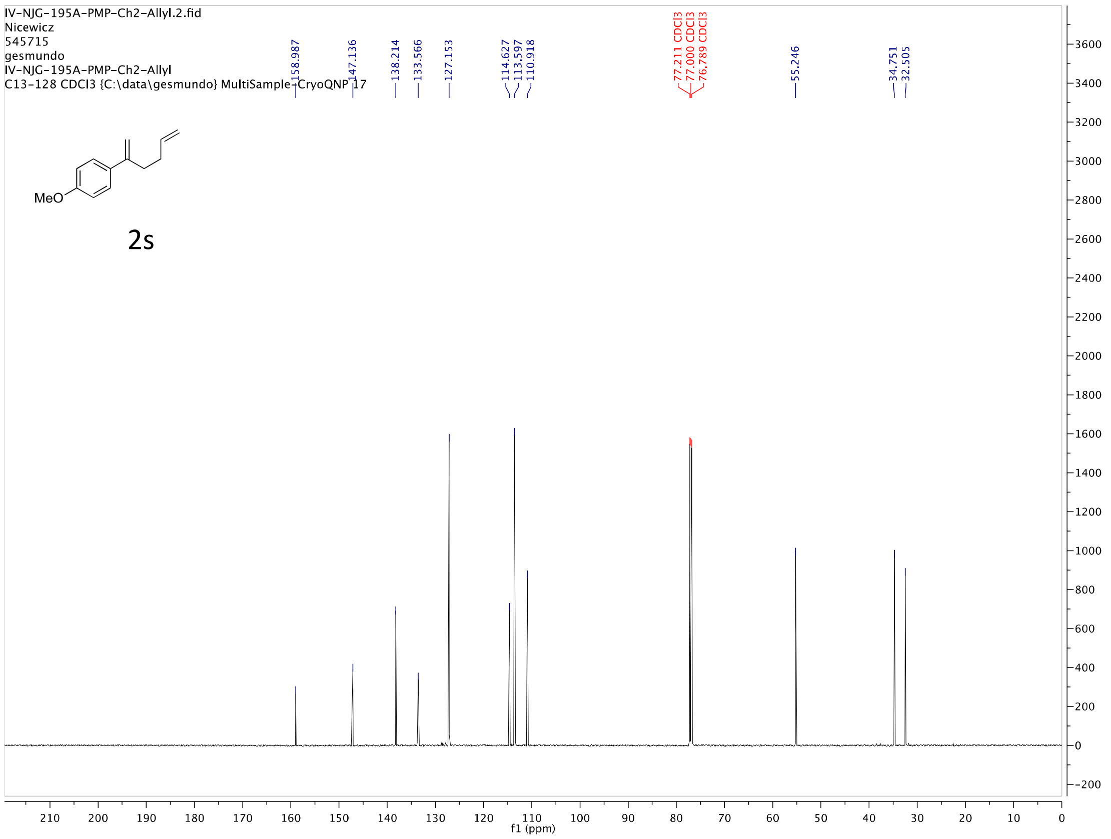
IV-NJG-195A-PMP-Ch2-Allyl.2.fid
Nicewicz
545715
gesmundo
IV-NJG-195A-PMP-Ch2-Allyl
C13-128 CDCl3 {C:\data\gesmundo} MultiSample-CryoQNP 17
158.987
147.136
138.214
133.566
127.153
114.627
113.597
110.918
77.211 CDCl3
77.000 CDCl3
76.789 CDCl3
55.246
34.751
32.505
MeO
2s
f1 (ppm)

1-Methoxy-4-(3-((3-methylbut-2-en-1-yl)oxy)prop-1-en-2-yl)benzene (2t)

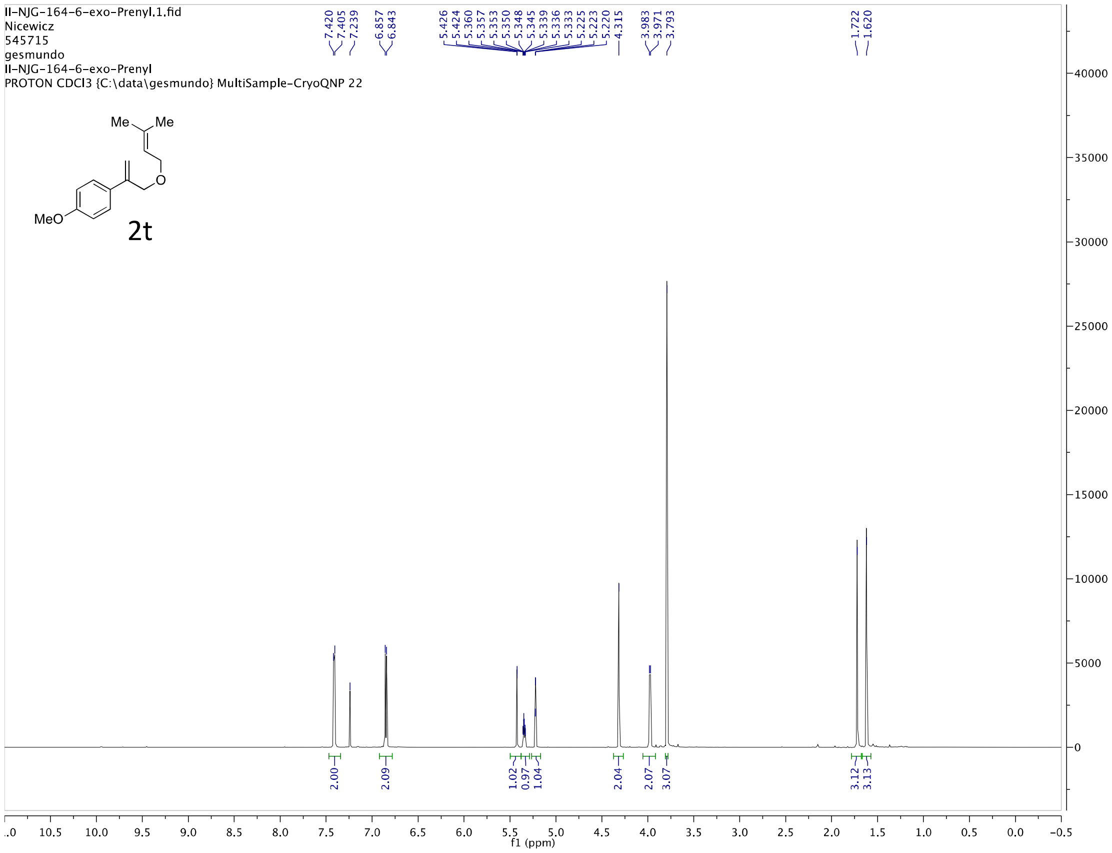

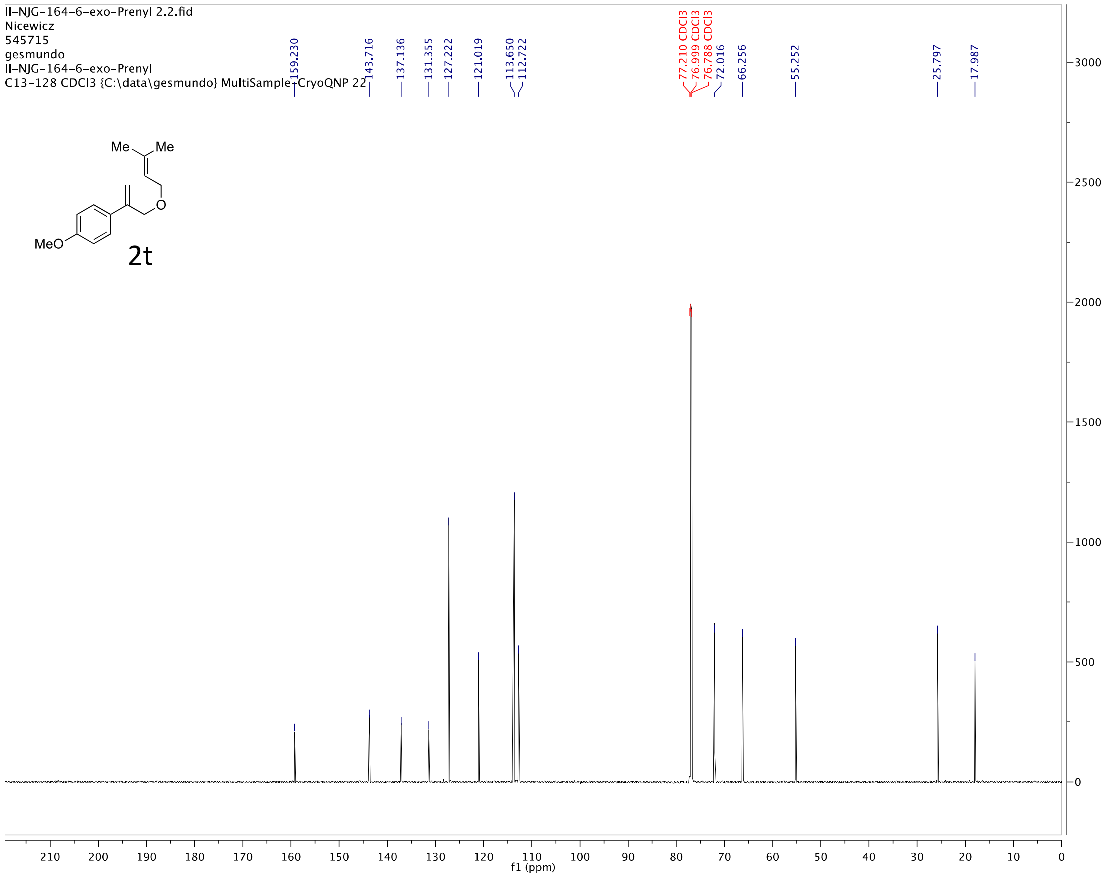
II-NJG-164-6-exo-Prenyl 2.2.fid
Nicewicz
545715
gesmundo
II-NJG-164-6-exo-Prenyl
C13-128 CDCl3 {C:\data\gesmundo} MultiSample-CryoQNP 22
159.230
143.716
137.136
131.355
127.222
121.019
113.650
112.722
77.210 CDCl3
76.999 CDCl3
76.788 CDCl3
72.016
66.256
55.252
25.797
17.987
Me
Me
O
MeO
2t
f1 (ppm)

(*E*)-1-Methoxy-4-(3-((3-methylbut-2-en-1-yl)oxy)prop-1-en-1-yl)benzene (2v)

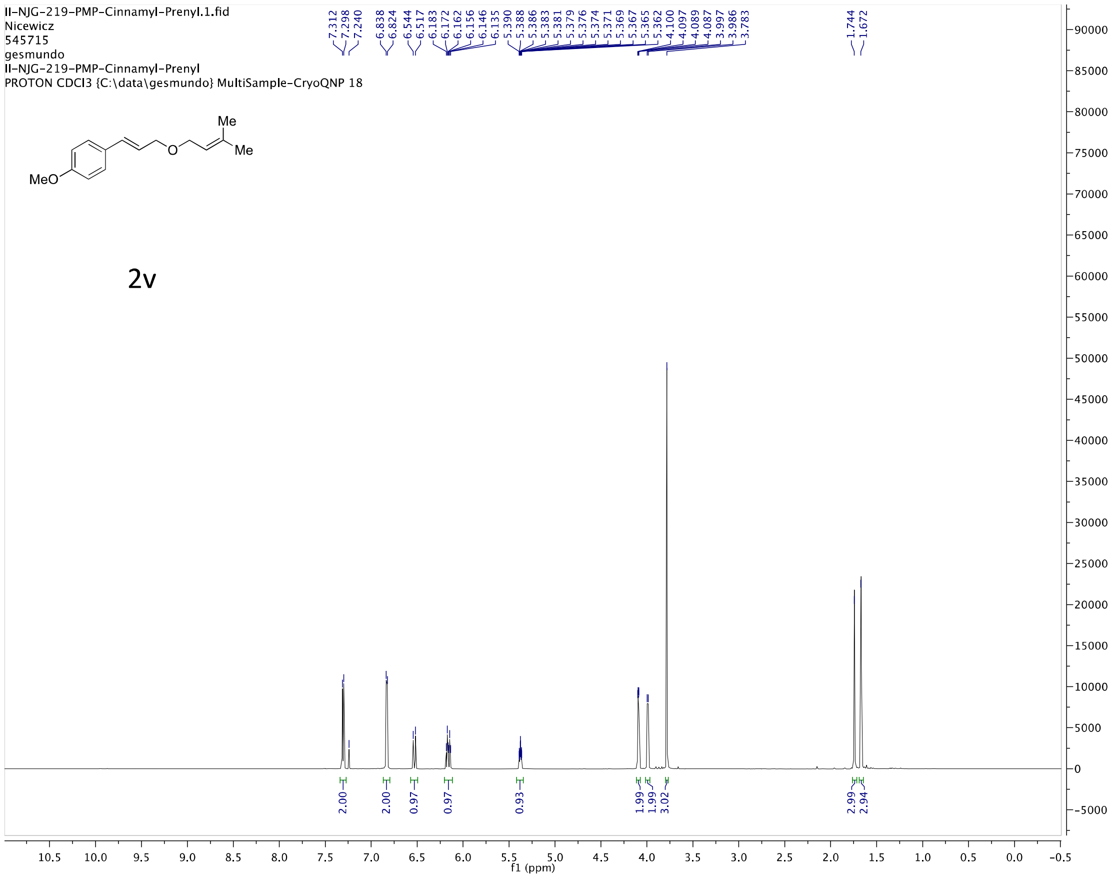

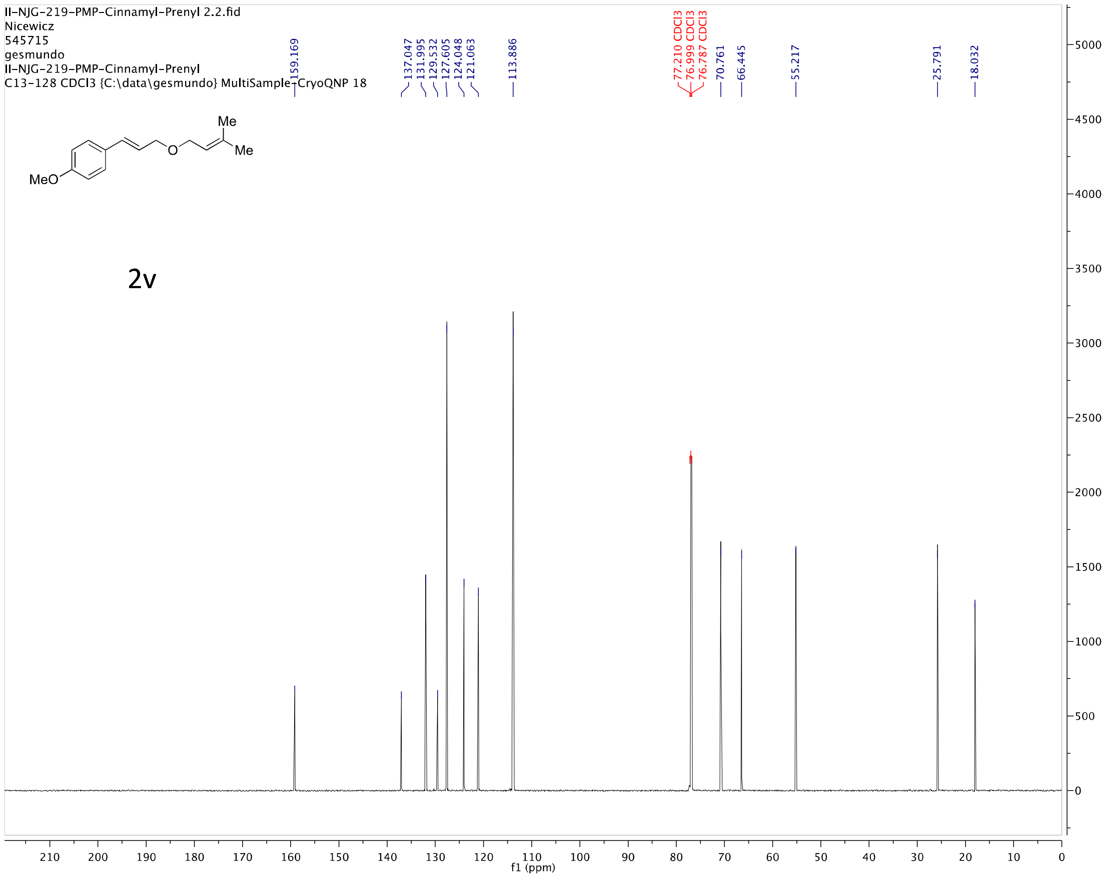
II-NJG-219-PMP-Cinnamyl-Prenyl 2.2.fid
Nicewicz
545715
gesmundo
II-NJG-219-PMP-Cinnamyl-Prenyl
C13-128 CDCl3 {C:\data\gesmundo} MultiSample-CryoQNP 18
159.169
137.047
131.995
129.532
127.605
124.048
121.063
113.886
77.210 CDCl3
76.999 CDCl3
76.787 CDCl3
70.761
66.445
55.217
25.791
18.032
Me
Me
MeO
O
2v
f1 (ppm)

1-(4-Methoxyphenyl)-4,4,7,7-tetramethyl-2,3-dioxabicyclo[3.2.1]octane (3a)

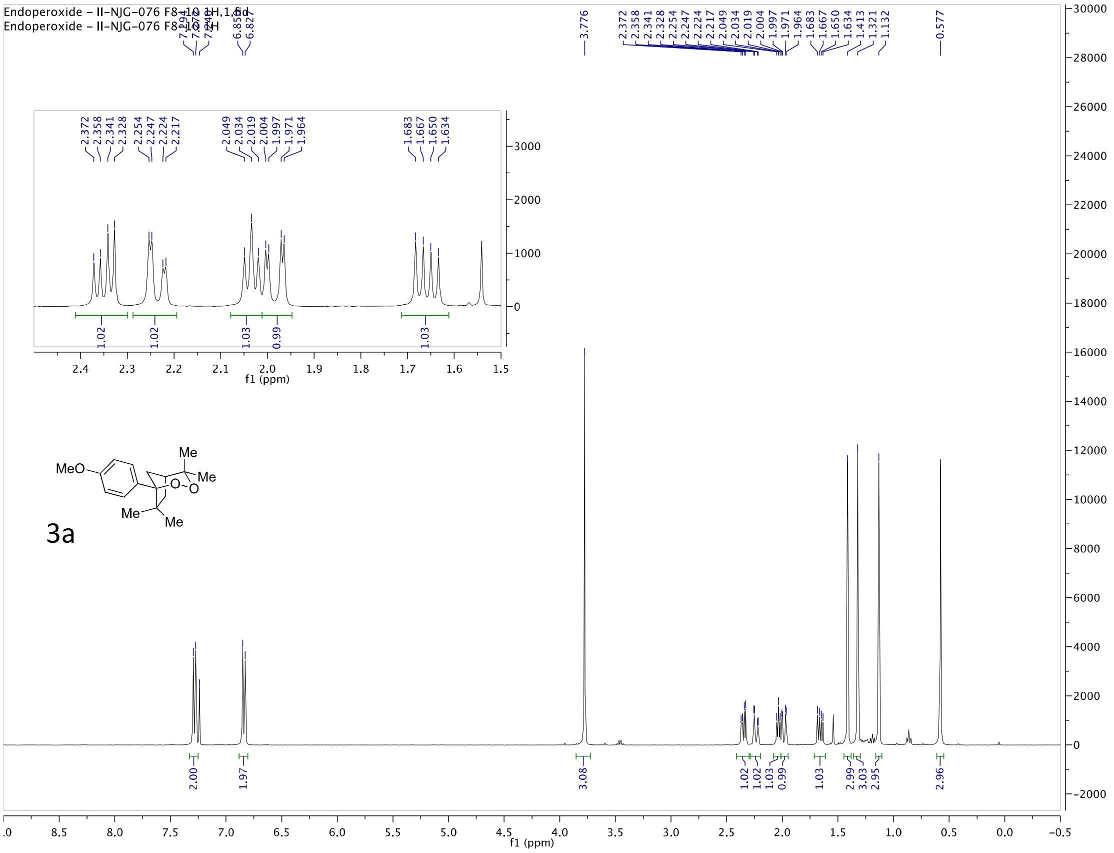

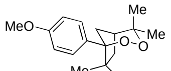
MeO
Me
Me
O
O
Me
Me

3a

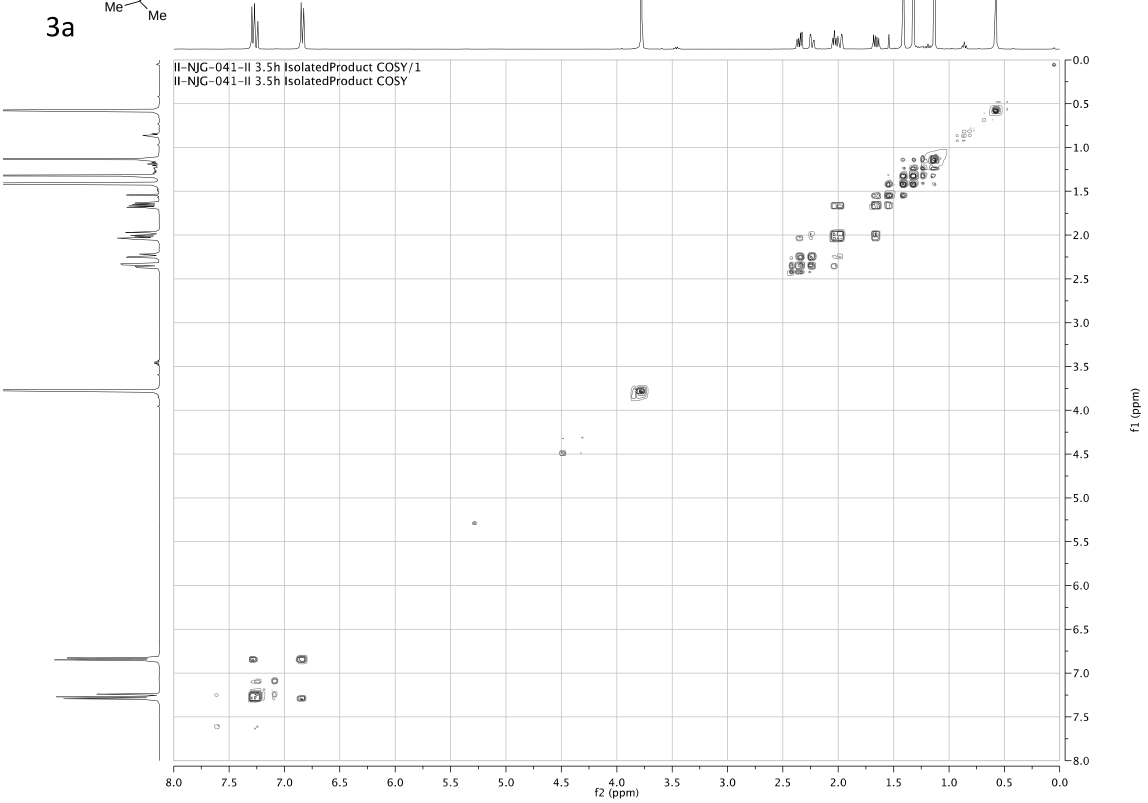
II-NJG-041-II 3.5h IsolatedProduct COSY/1
II-NJG-041-II 3.5h IsolatedProduct COSY
f2 (ppm)
f1 (ppm)

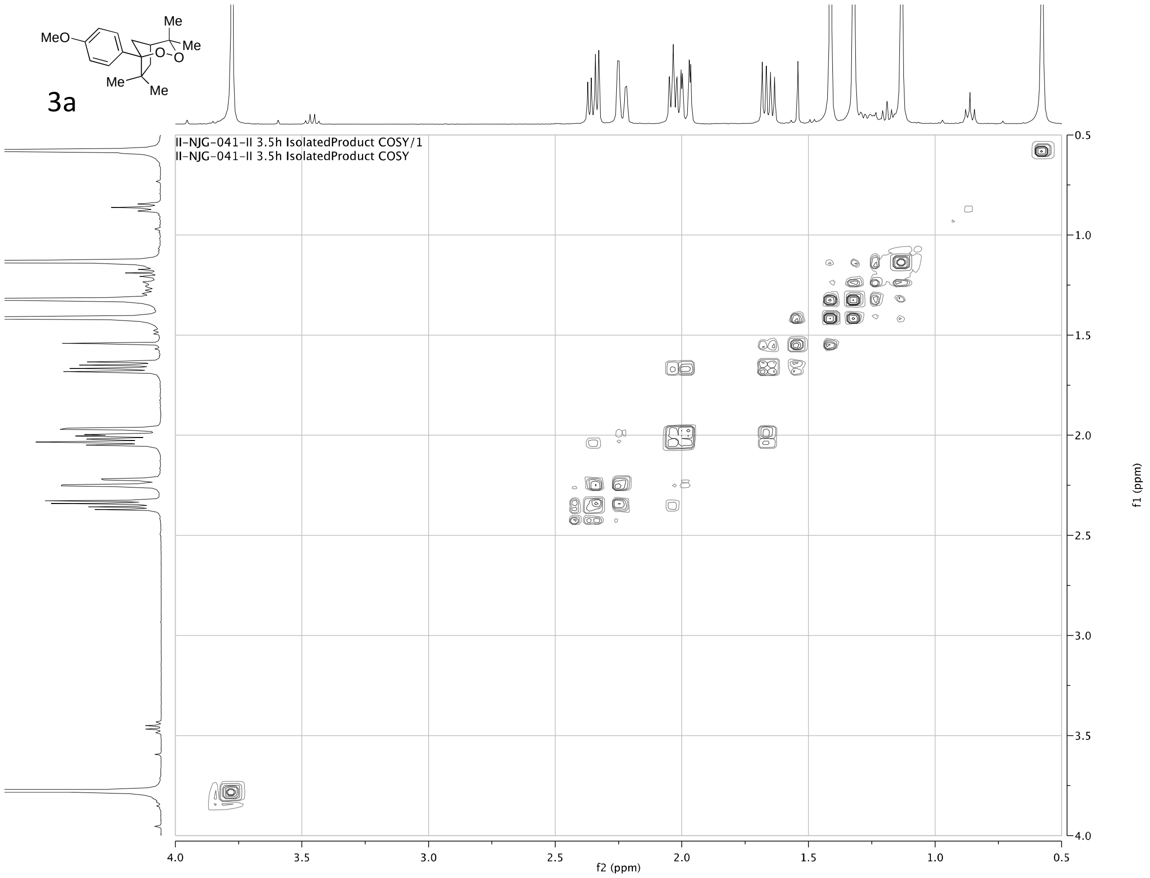

MeO
Me
Me
O
O
Me
Me
3a
II-NJG-041-II 3.5h IsolatedProduct COSY/1
II-NJG-041-II 3.5h IsolatedProduct COSY
f2 (ppm)
f1 (ppm)

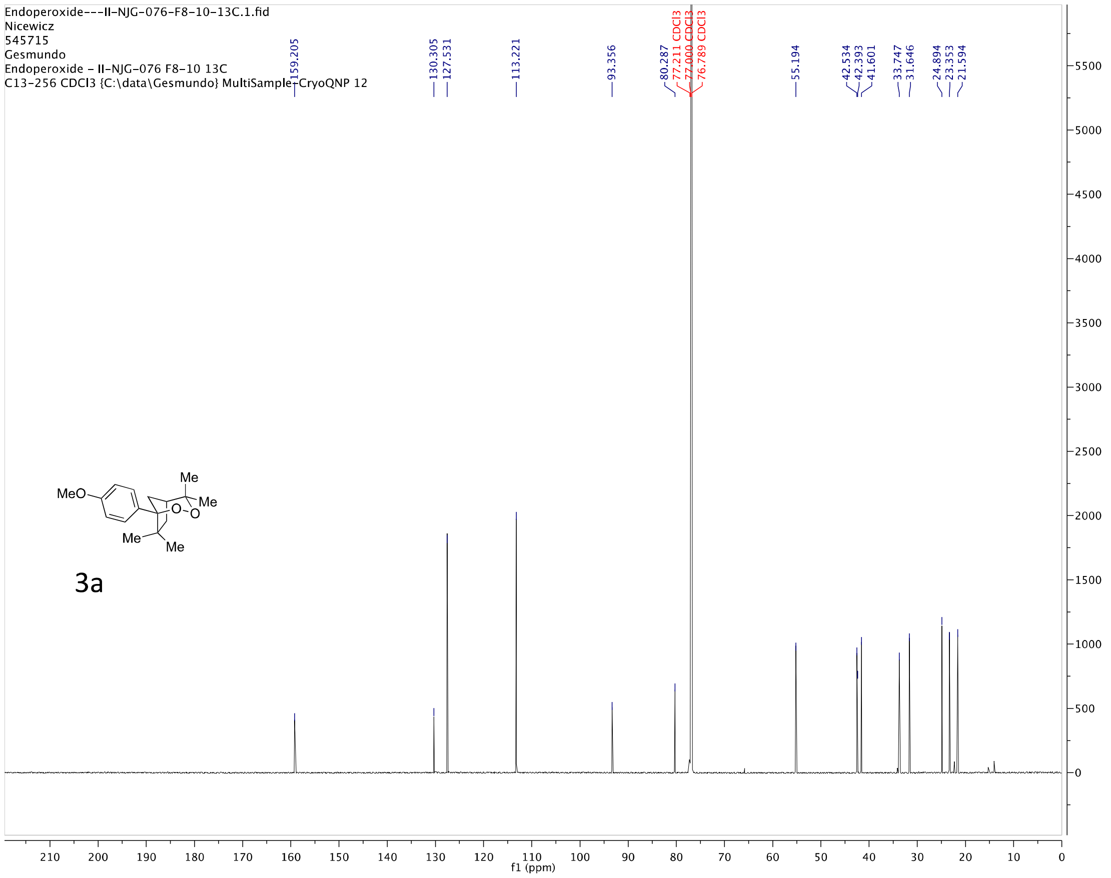

Endoperoxide---II-NJG-076-F8-10-13C.1.fid
Nicewicz
545715
Gesmundo
Endoperoxide - II-NJG-076 F8-10 13C
C13-256 CDCl3 {C:\data\Gesmundo} MultiSample-CryoQNP 12
159.205
130.305
127.531
113.221
93.356
80.287
77.211 CDCl3
77.000 CDCl3
76.789 CDCl3
55.194
42.534
42.393
41.601
33.747
31.646
24.894
23.353
21.594
MeO
Me
Me
O
O
Me
Me
3a
f1 (ppm)

1-(4-Methoxyphenyl)-4,4-dimethyl-2,3-dioxabicyclo[3.2.1]octane (3f)

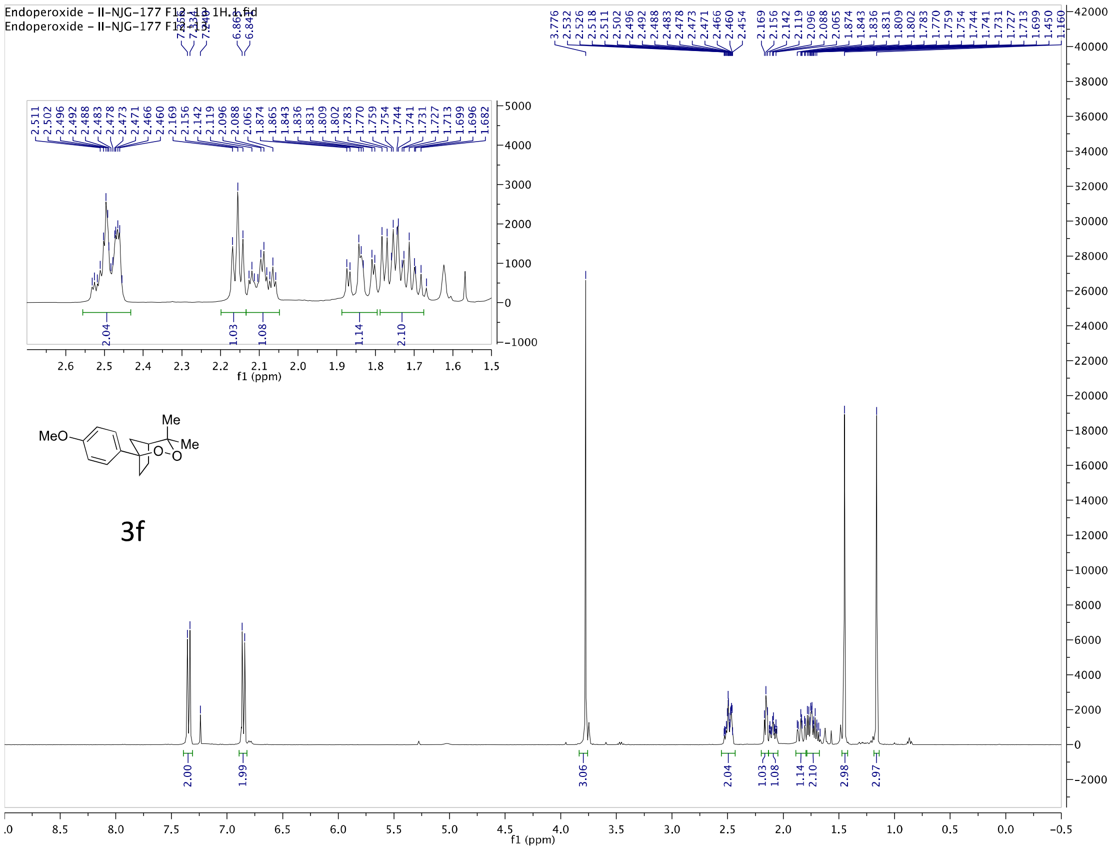

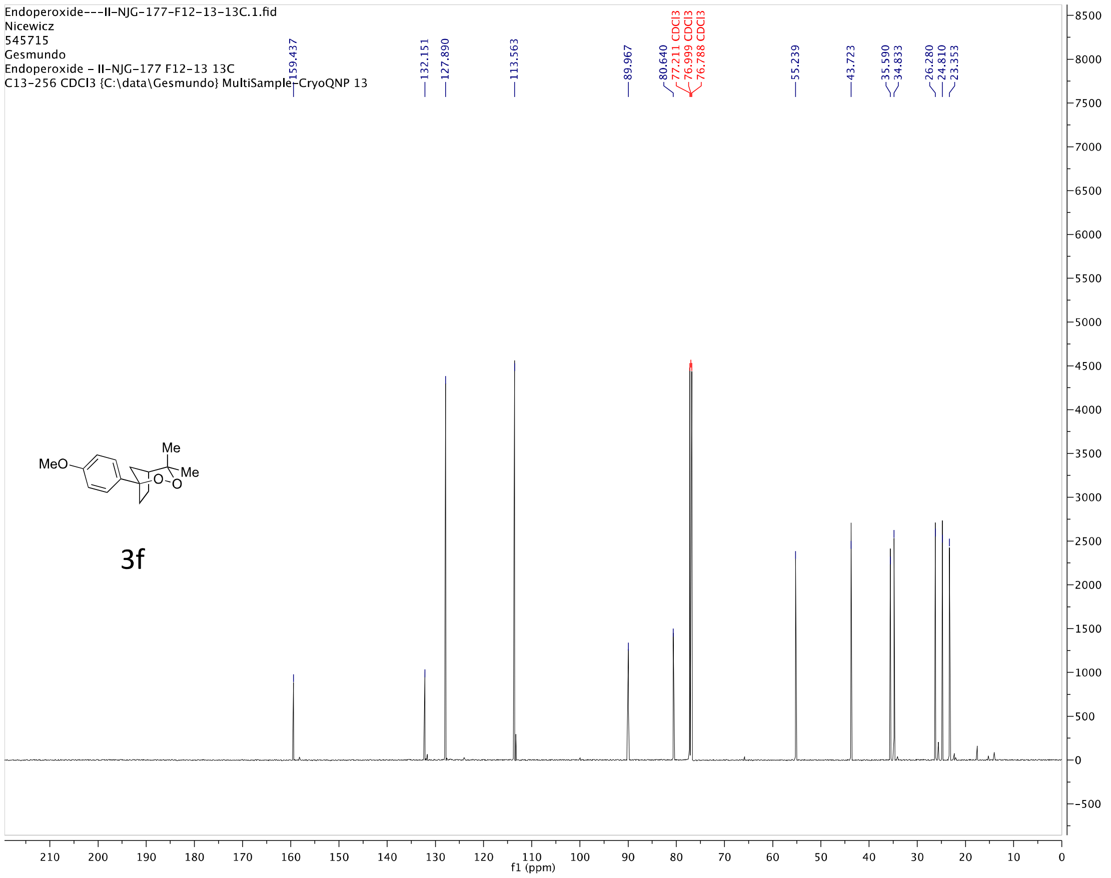
Endoperoxide---II-NJG-177-F12-13-13C.1.fid
Nicewicz
545715
Gesmundo
Endoperoxide - II-NJG-177 F12-13 13C
C13-256 CDCl3 {C:\data\Gesmundo} MultiSample-CryoQNP 13
159.437
132.151
127.890
113.563
89.967
80.640
77.211 CDCl3
76.999 CDCl3
76.788 CDCl3
55.239
43.723
35.590
34.833
26.280
24.810
23.353
MeO
Me
Me
O
O
f1 (ppm)

3f

1-(2,4-Dimethoxyphenyl)-4,4-dimethyl-2,3-dioxabicyclo[3.2.1]octane (3g)

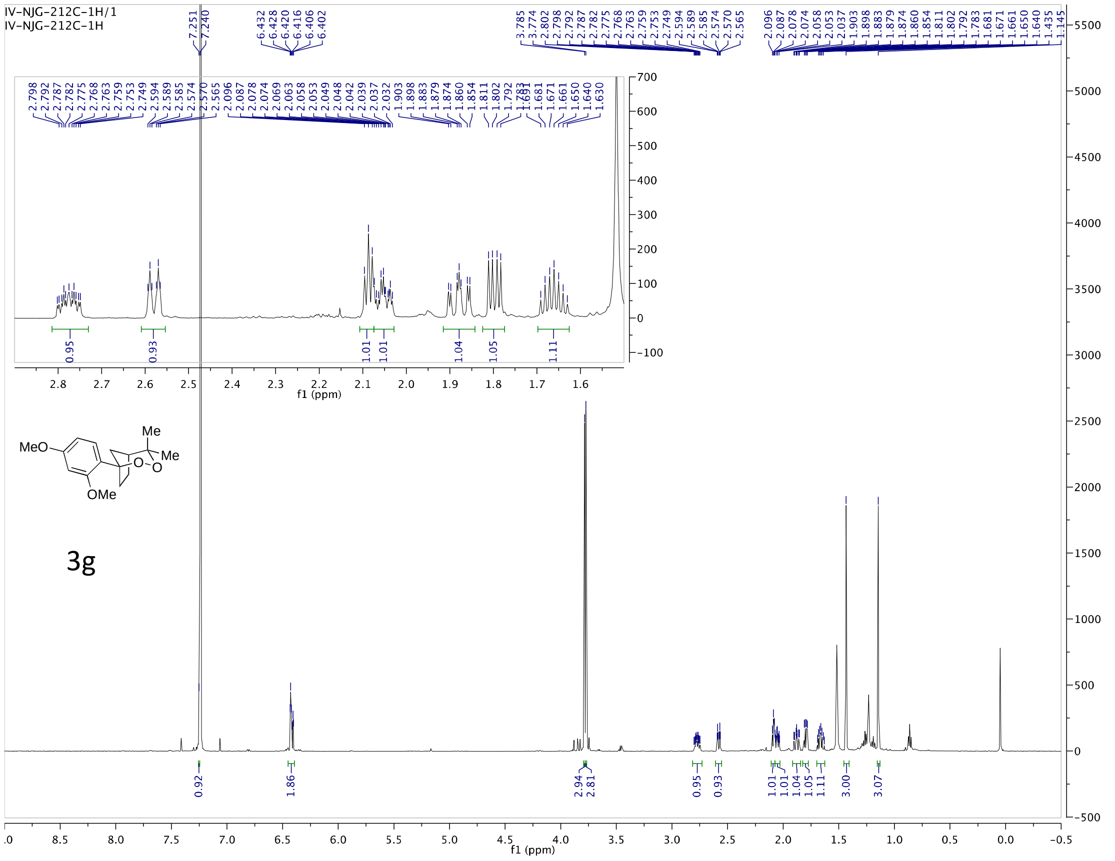

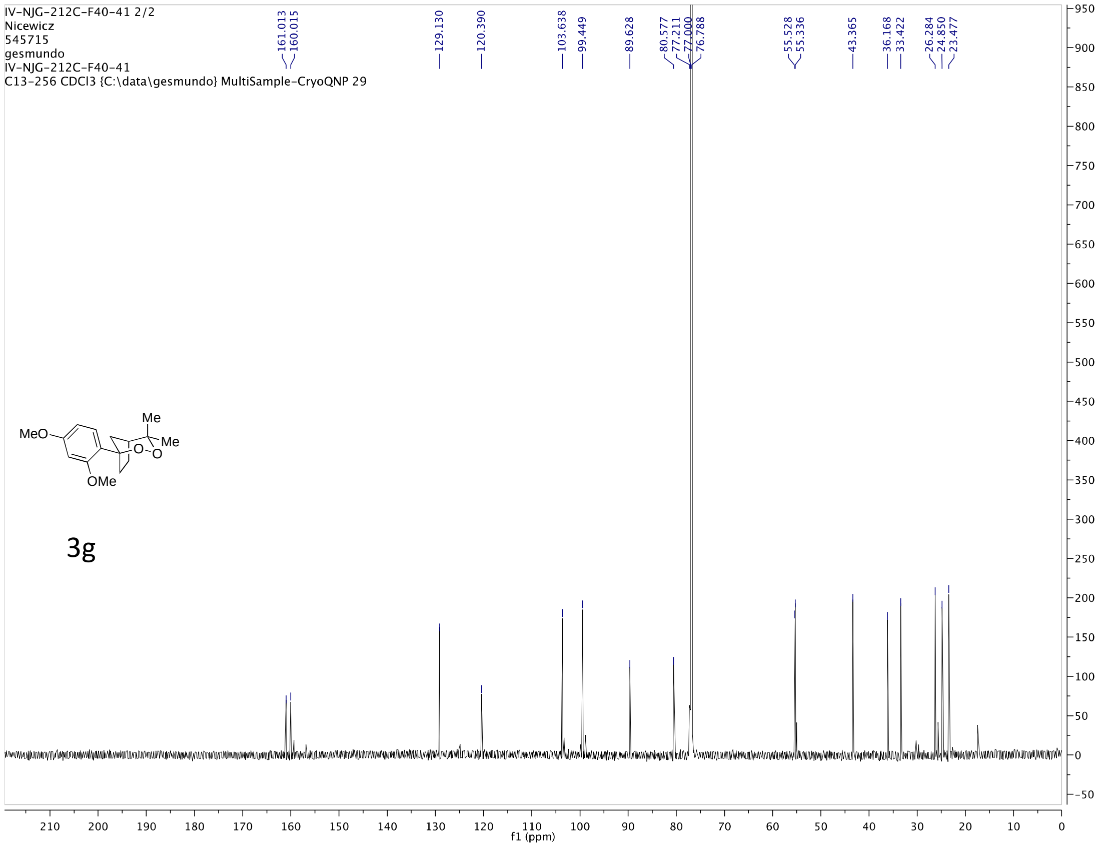
IV-NJG-212C-F40-41 2/2
Nicewicz
545715
gesmundo
IV-NJG-212C-F40-41
C13-256 CDCl3 {C:\data\gesmundo} MultiSample-CryoQNP 29
161.013
160.015
129.130
120.390
103.638
99.449
89.628
80.577
77.211
77.000
76.788
55.528
55.336
43.365
36.168
33.422
26.284
24.850
23.477
MeO
OMe
Me
Me
O
O
f1 (ppm)

3g

1-(2-Methoxyphenyl)-4,4-dimethyl-2,3-dioxabicyclo[3.2.1]octane (3i)

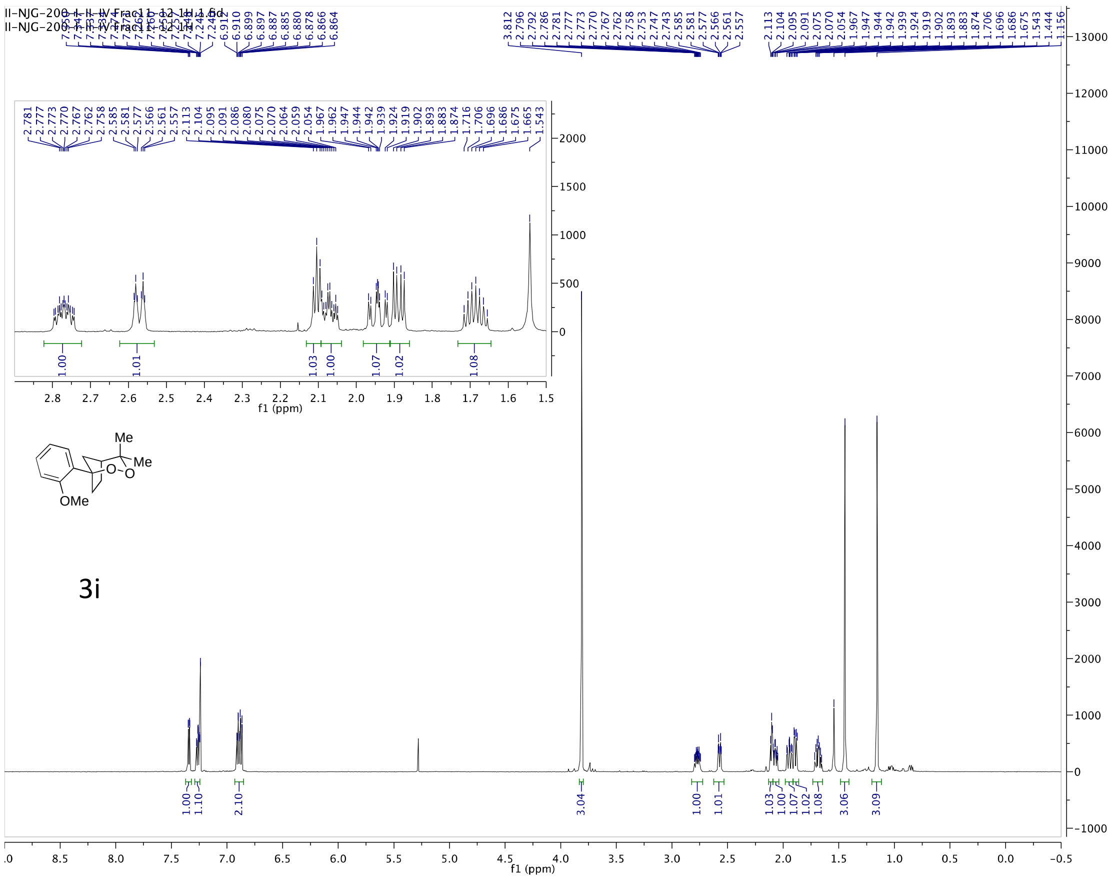

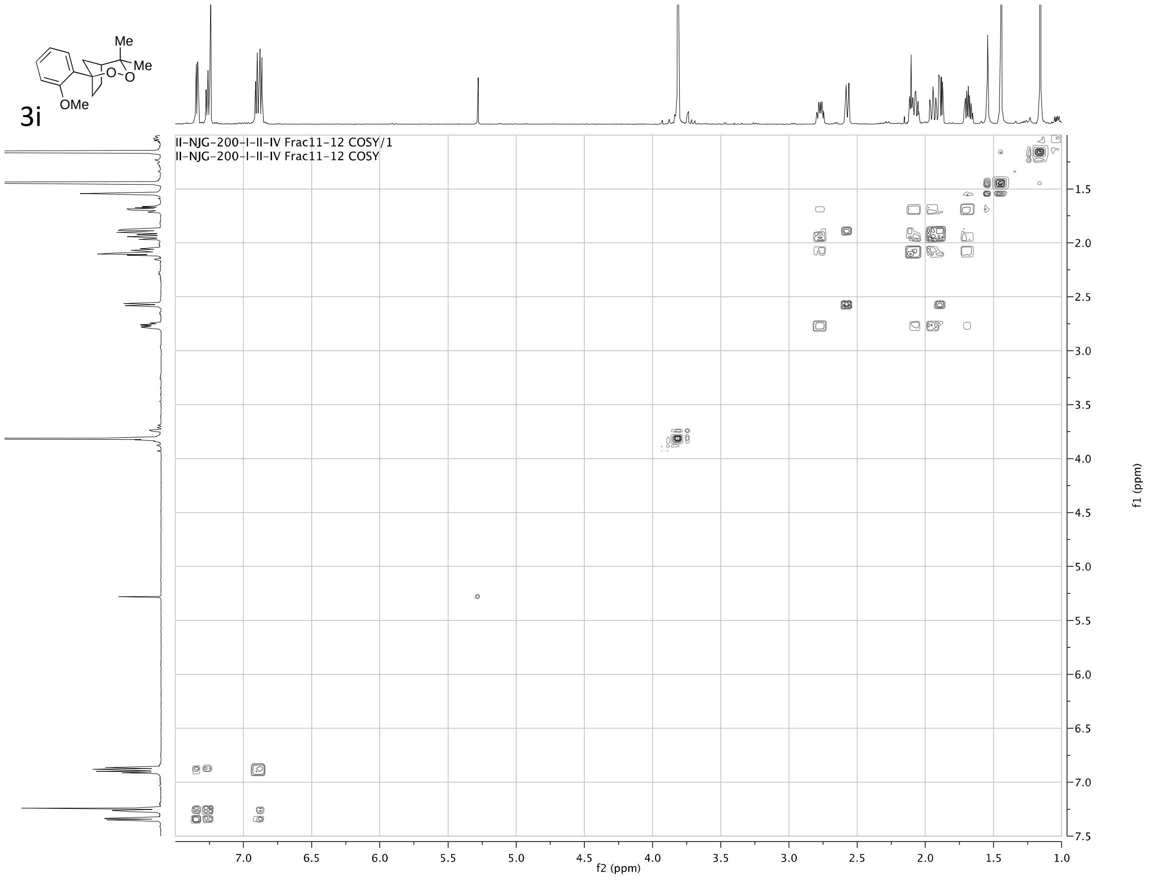
Me
Me
O–O
OMe
3i
II-NJG-200-I-II-IV Frac11-12 COSY/1
II-NJG-200-I-II-IV Frac11-12 COSY
f2 (ppm)
f1 (ppm)

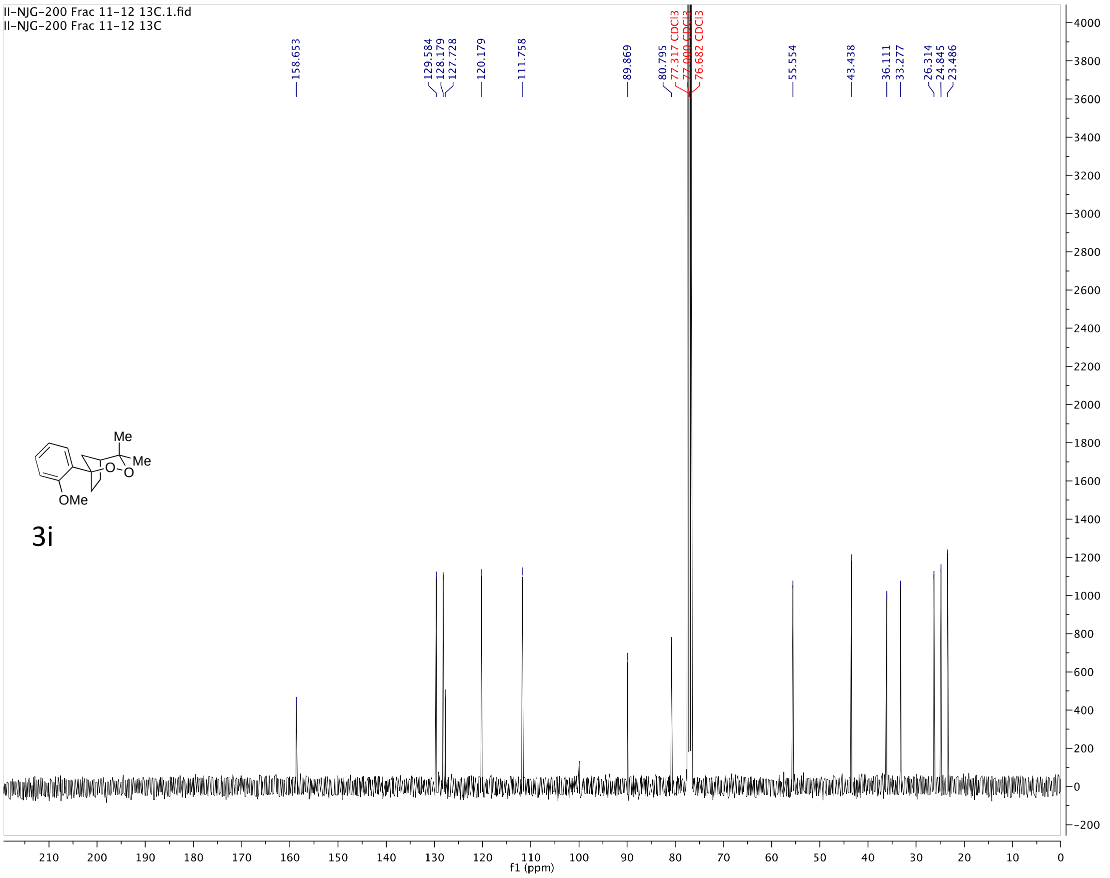

II-NJG-200 Frac 11-12 13C.1.fid
II-NJG-200 Frac 11-12 13C
158.653
129.584
128.179
127.728
120.179
111.758
89.869
80.795
77.317 CDCl3
77.000 CDCl3
76.682 CDCl3
55.554
43.438
36.111
33.277
26.314
24.845
23.486
Me
Me
O
O
OMe
3i
f1 (ppm)

1-(4-Methoxyphenyl)-7,7-dimethyl-4-phenyl-2,3-dioxabicyclo[3.2.1]octane (3m)

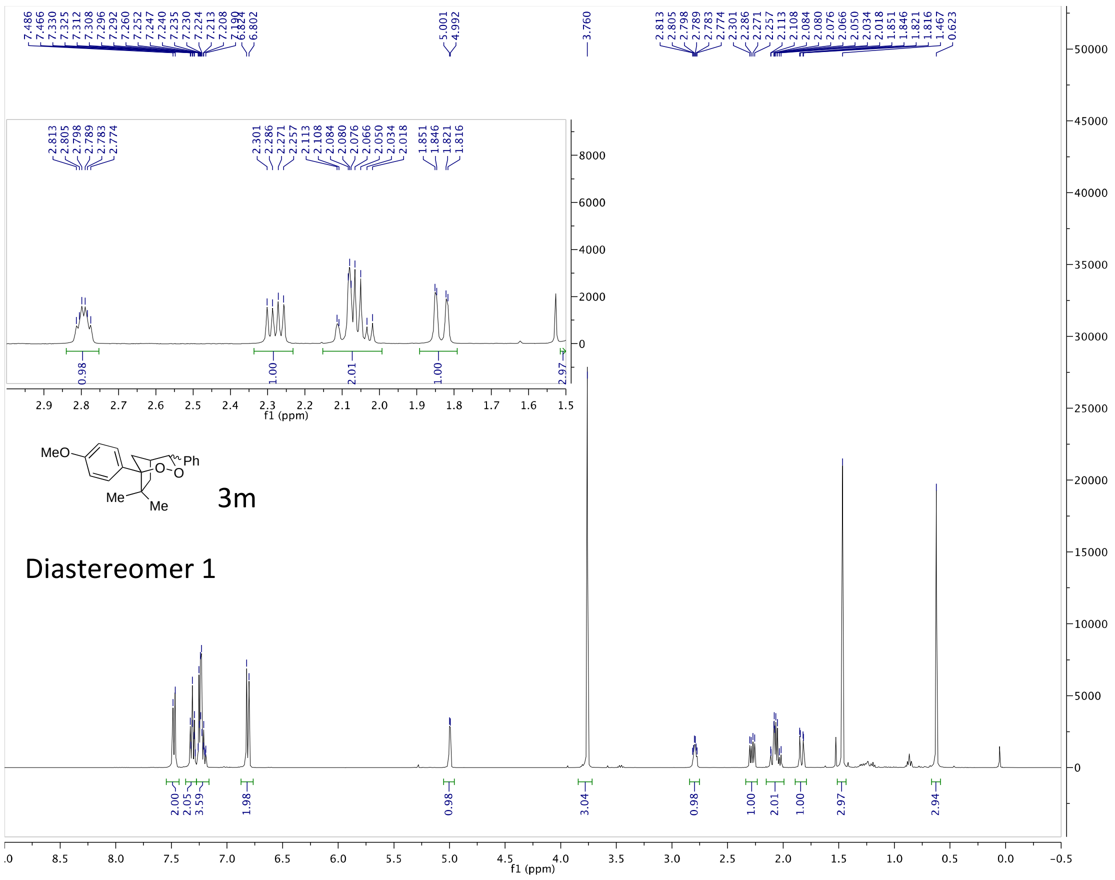

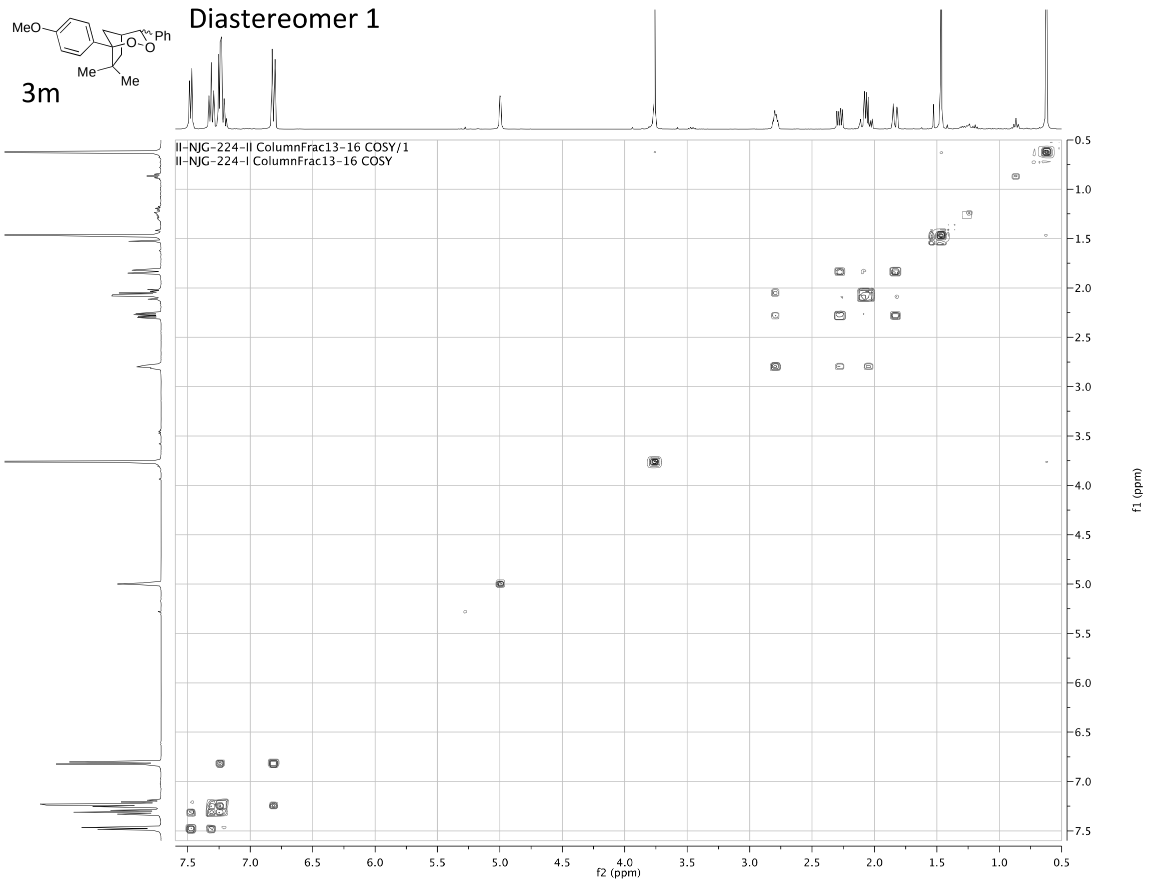

MeO
Ph
Me
Me
3m
Diastereomer 1
II-NJG-224-II ColumnFrac13-16 COSY/1
II-NJG-224-I ColumnFrac13-16 COSY
f2 (ppm)
f1 (ppm)

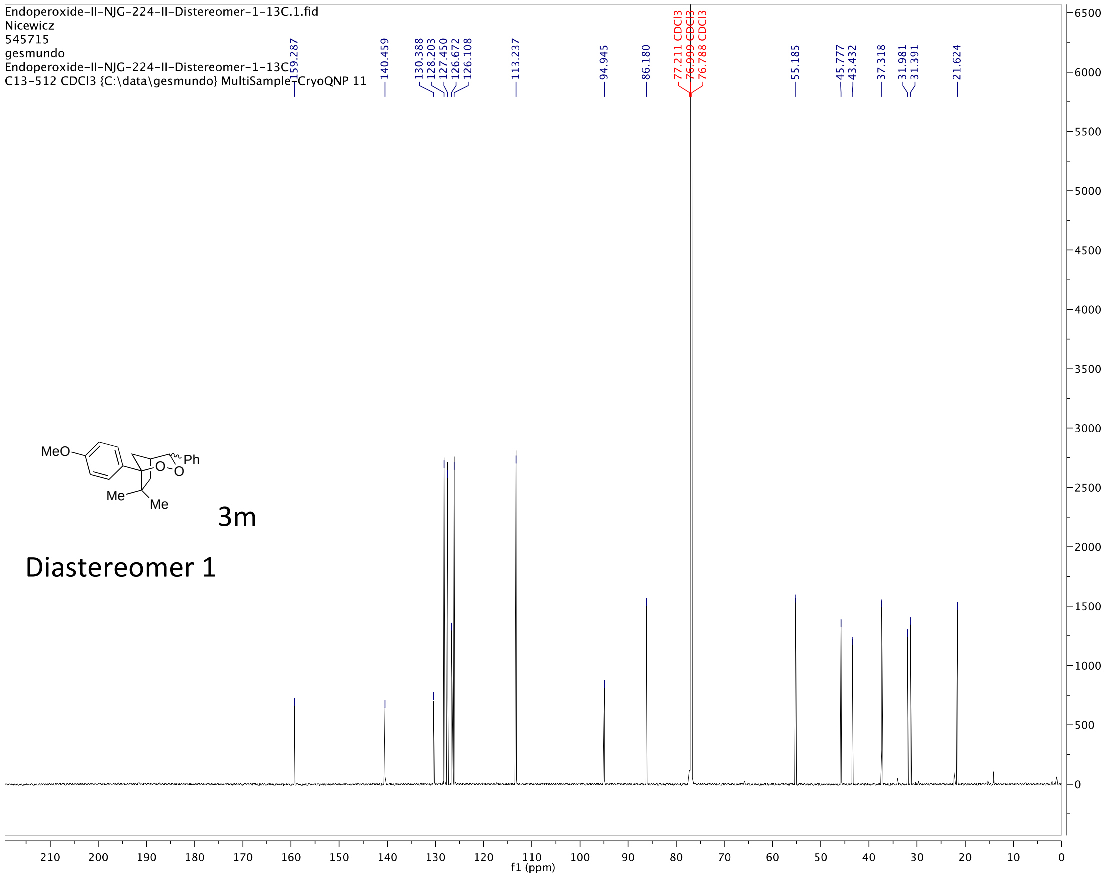
Endoperoxide-II-NJG-224-II-Distereomer-1-13C.1.fid
Nicewicz
545715
gesmundo
Endoperoxide-II-NJG-224-II-Distereomer-1-13C
C13-512 CDCl3 {C:\data\gesmundo} MultiSample-CryoQNP 11
159.287
140.459
130.388
128.203
127.450
126.672
126.108
113.237
94.945
86.180
77.211 CDCl3
76.999 CDCl3
76.788 CDCl3
55.185
45.777
43.432
37.318
31.981
31.391
21.624
MeO
Ph
O
O
Me
Me
3m
Diastereomer 1
f1 (ppm)

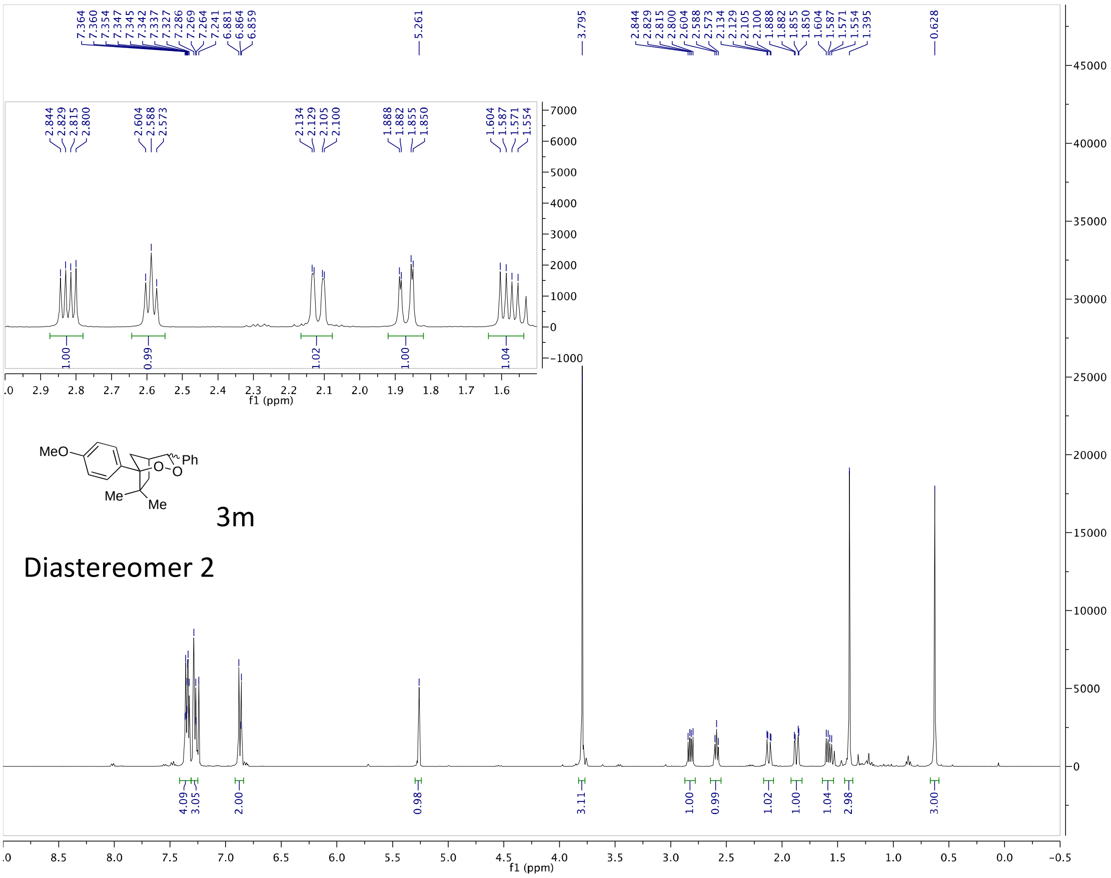
MeO
Ph
O
O
Me
Me
3m
Diastereomer 2
f1 (ppm)

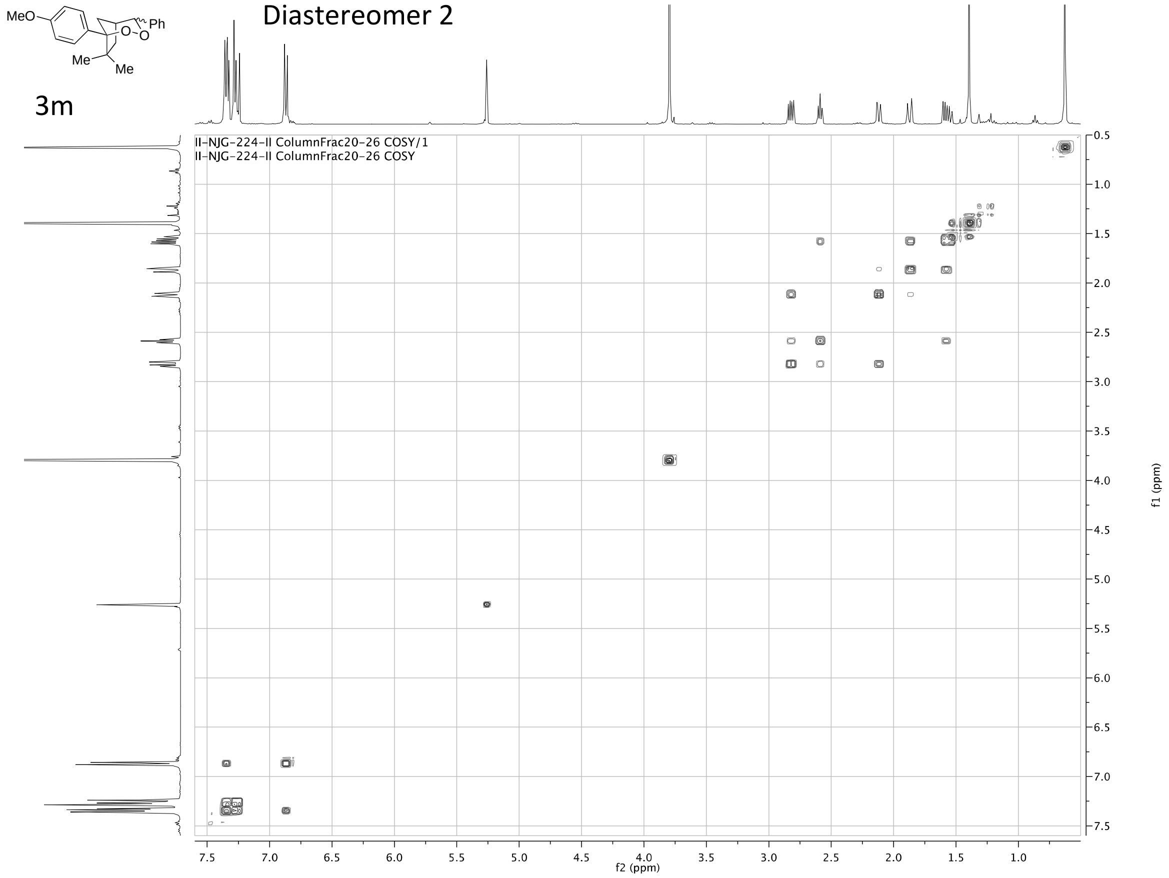
MeO
Ph
O
O
Me
Me
3m
Diastereomer 2
II-NJG-224-II ColumnFrac20-26 COSY/1
II-NJG-224-II ColumnFrac20-26 COSY
f2 (ppm)
f1 (ppm)

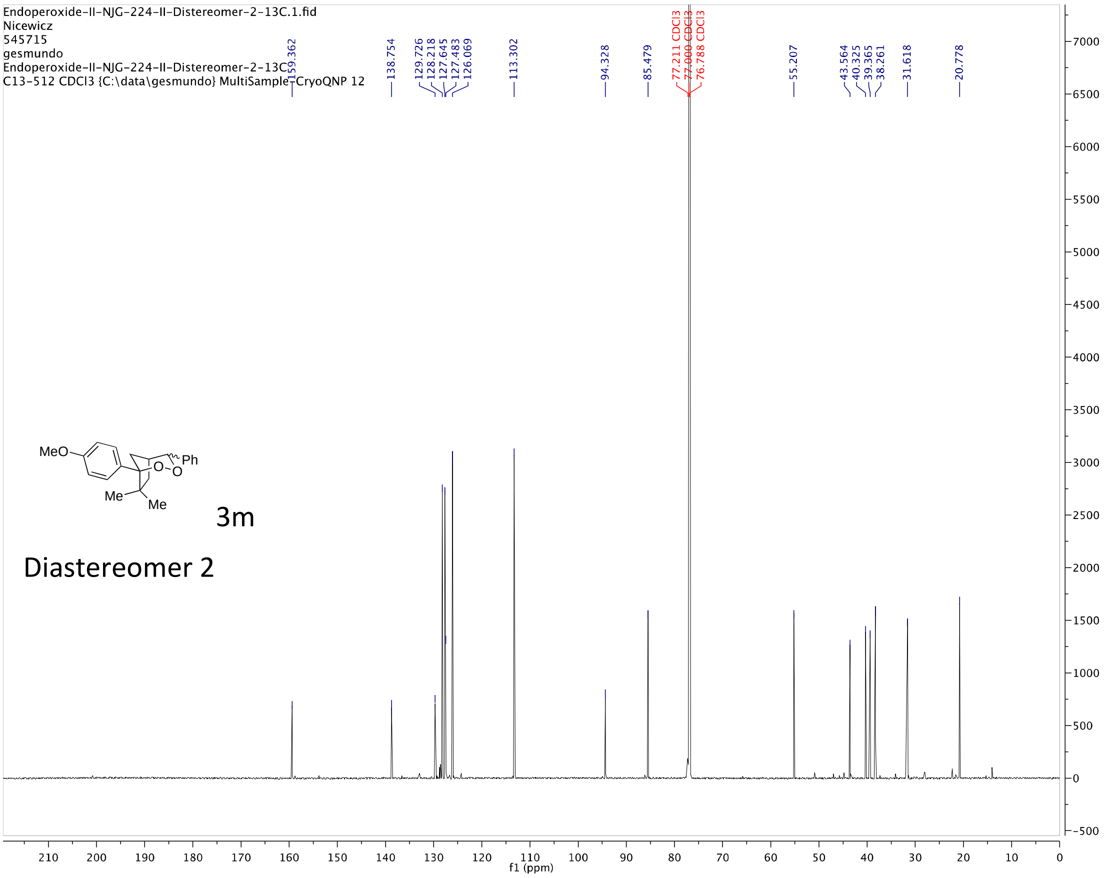
Endoperoxide-II-NJG-224-II-Distereomer-2-13C.1.fid
Nicewicz
545715
gesmundo
Endoperoxide-II-NJG-224-II-Distereomer-2-13C
C13-512 CDCl3 {C:\data\gesmundo} MultiSample-CryoQNP 12
159.362
138.754
129.726
128.218
127.645
127.483
126.069
113.302
94.328
85.479
77.211 CDCl3
77.000 CDCl3
76.788 CDCl3
55.207
43.564
40.325
39.365
38.261
31.618
20.778
MeO
O
O
Ph
Me
Me
3m
Diastereomer 2
f1 (ppm)

3-(4-Methoxyphenyl)-4,4,6,6,9a-pentamethylhexahydro-3*H*,6*H*-3,5a-methanobenzo[*c*][1,2]dioxepine (3n)

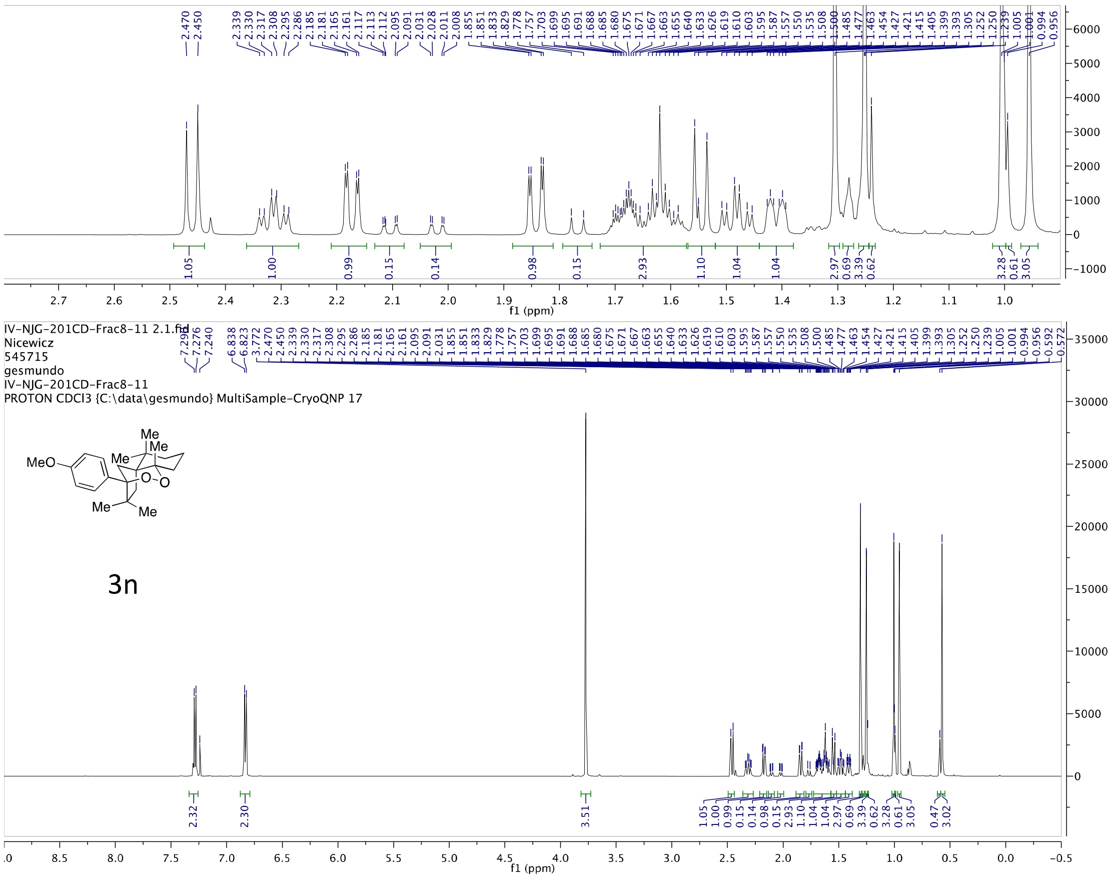

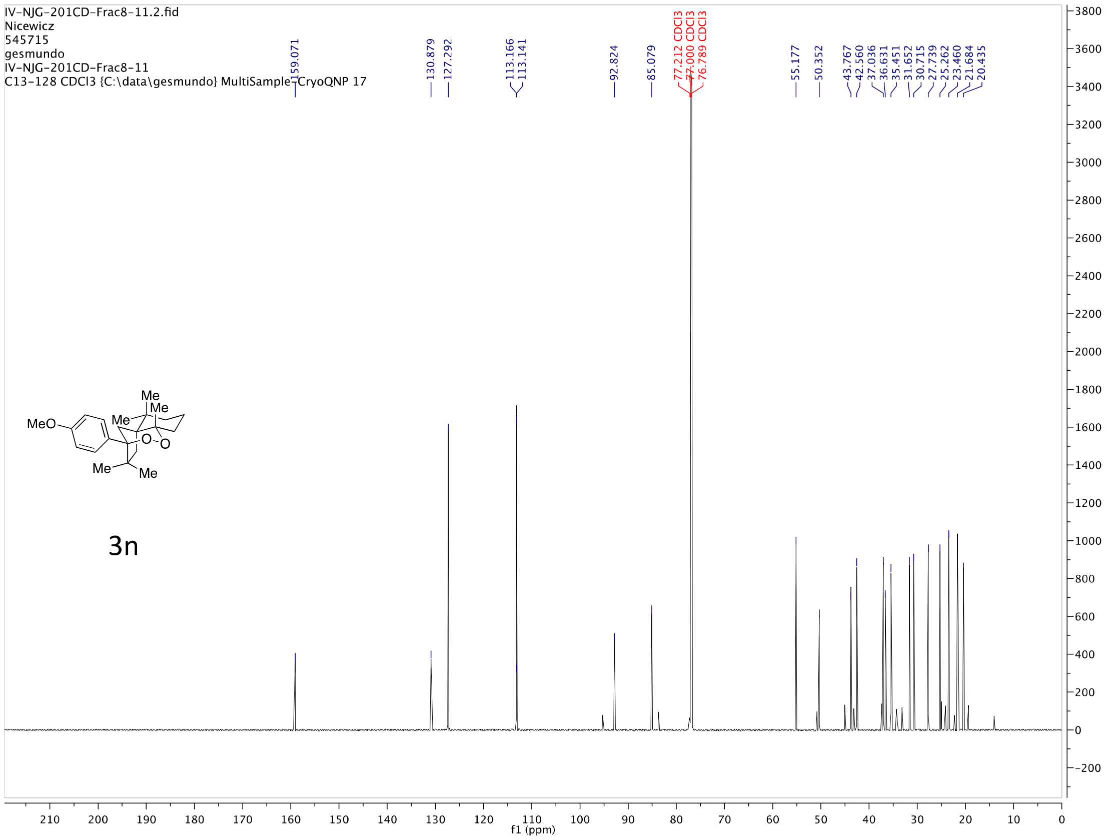
IV-NJG-201CD-Frac8-11.2.fid
Nicewicz
545715
gesmundo
IV-NJG-201CD-Frac8-11
C13-128 CDCl3 {C:\data\gesmundo} MultiSample-CryoQNP 17
159.071
130.879
127.292
113.166
113.141
92.824
85.079
77.212 CDCl3
77.000 CDCl3
76.789 CDCl3
55.177
50.352
43.767
42.560
37.036
36.631
35.451
31.652
30.715
27.739
25.262
23.460
21.684
20.435
MeO
Me
Me
Me
O
O
Me
Me
3n
f1 (ppm)

1-(4-Methoxyphenyl)-3-methylpent-4-en-1-one (3o)

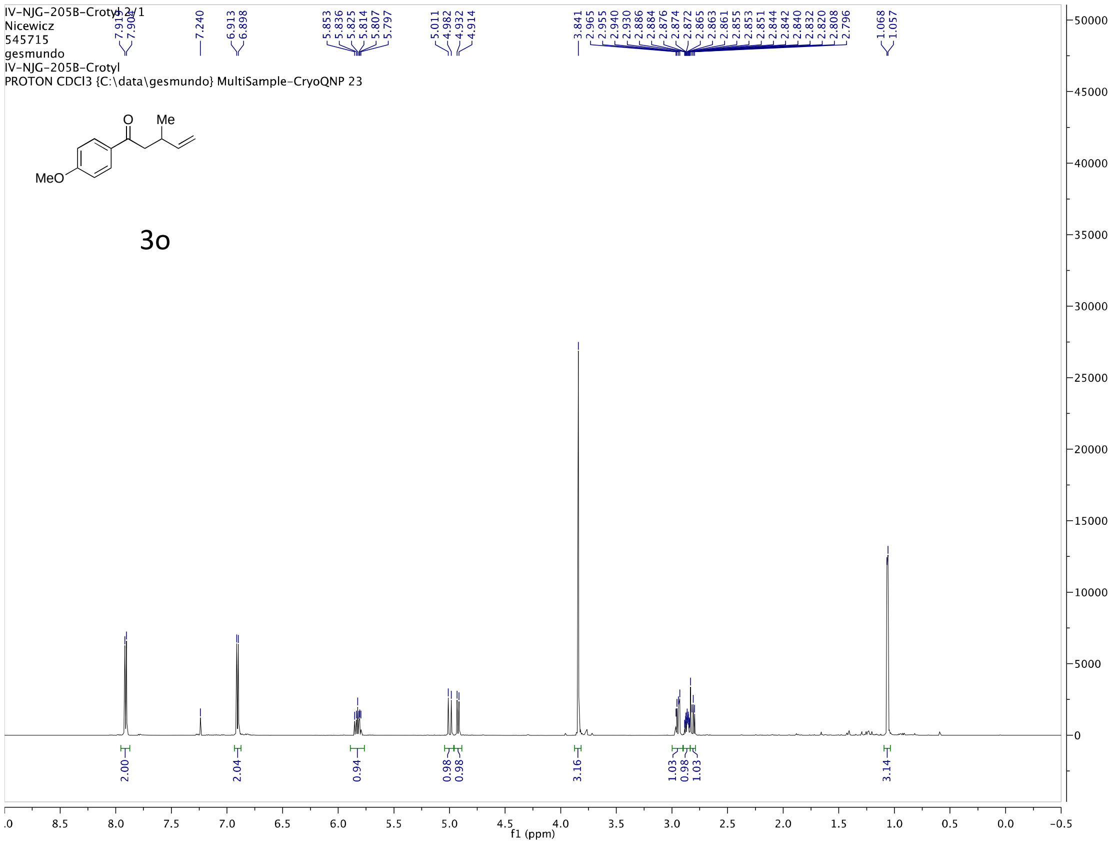

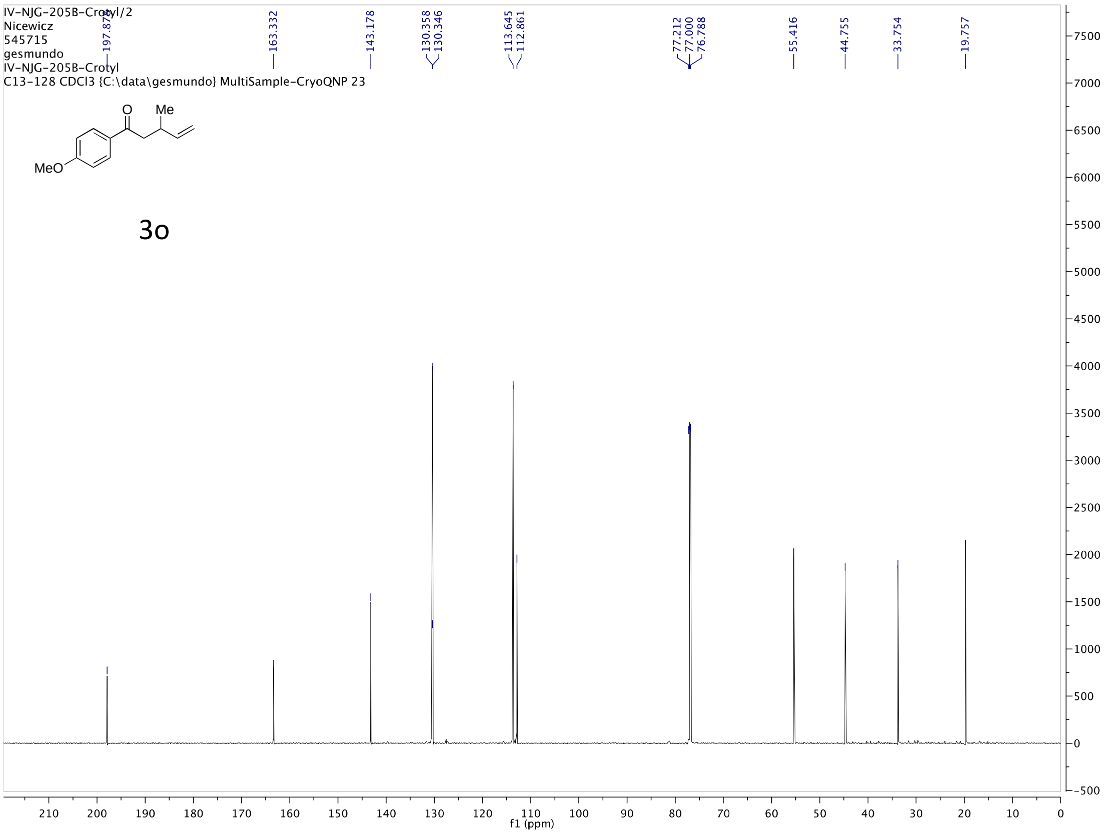
IV-NJG-205B-Crotyl/2
Nicewicz
545715
gesmundo
IV-NJG-205B-Crotyl
C13-128 CDCl3 {C:\data\gesmundo} MultiSample-CryoQNP 23
197.872
163.332
143.178
130.358
130.346
113.645
112.861
77.212
77.000
76.788
55.416
44.755
33.754
19.757
O
Me
MeO
3o
f1 (ppm)

1-(4-Methoxyphenyl)-4-methylpent-4-en-1-one (3p)

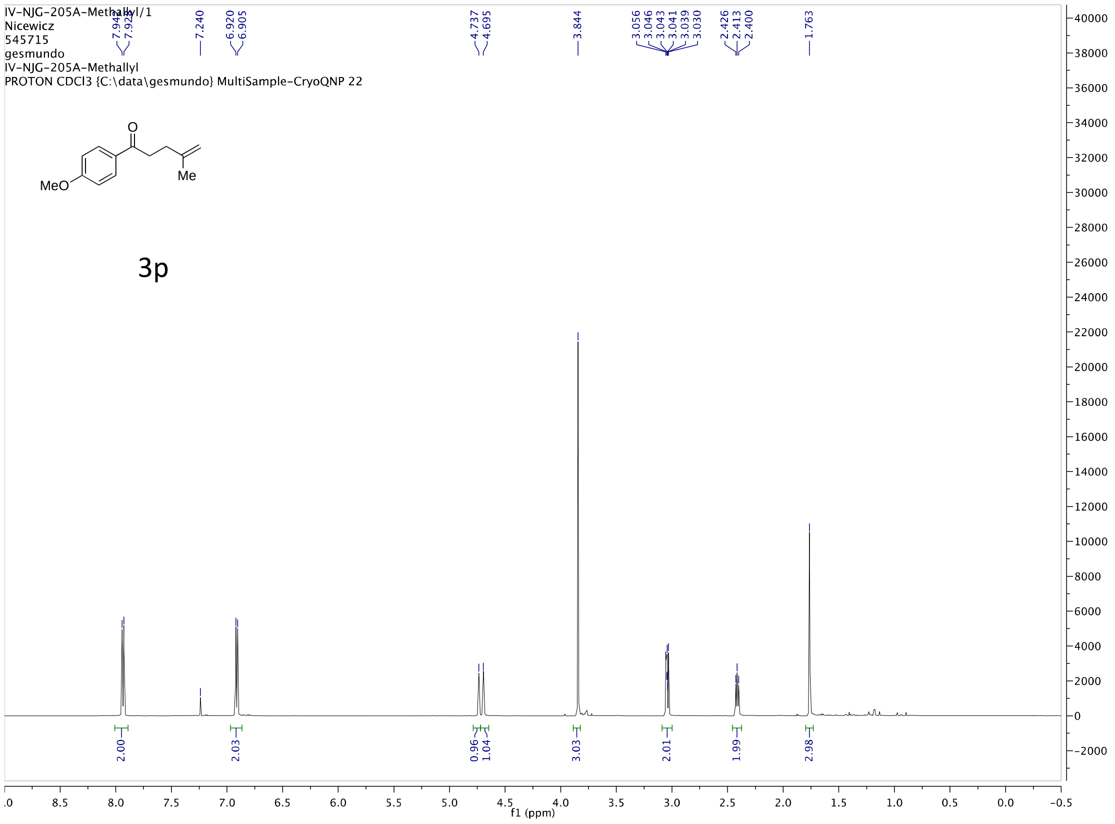

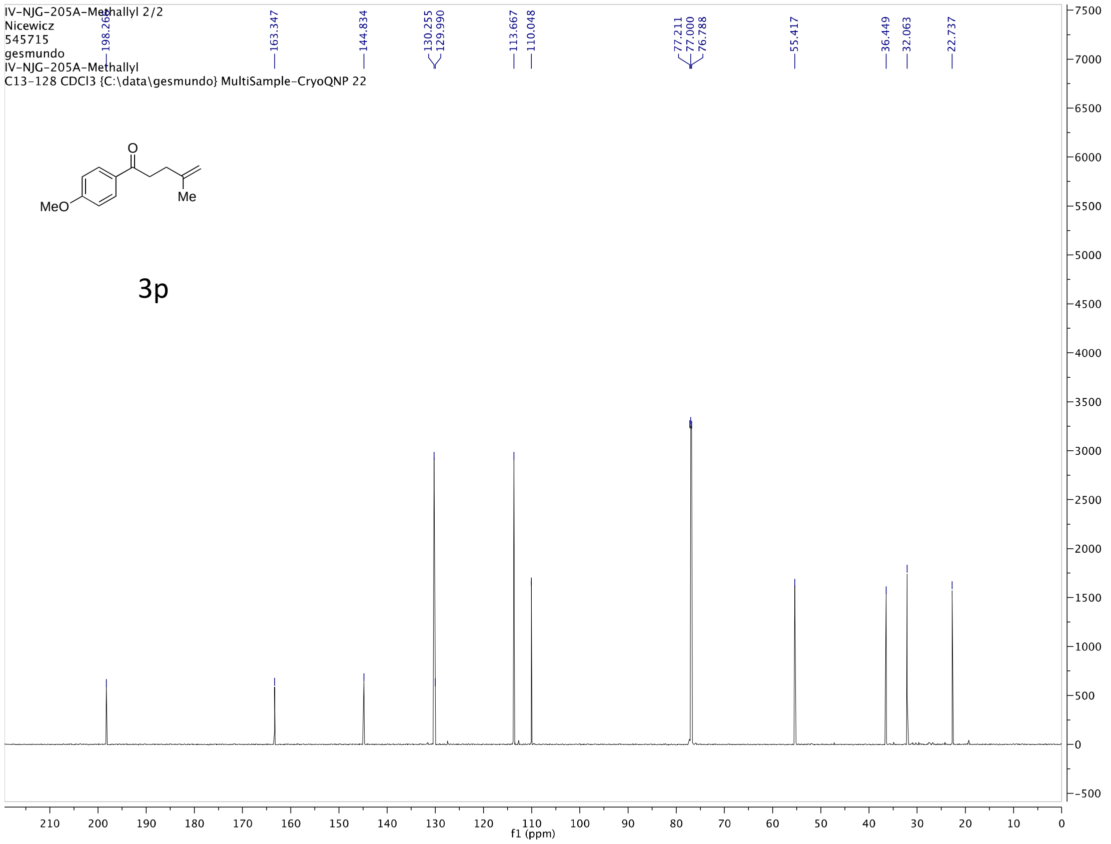
IV-NJG-205A-Methallyl 2/2
Nicewicz
545715
gesmundo
IV-NJG-205A-Methallyl
C13-128 CDCl3 {C:\data\gesmundo} MultiSample-CryoQNP 22
198.26
163.347
144.834
130.255
129.990
113.667
110.048
77.211
77.000
76.788
55.417
36.449
32.063
22.737
MeO
O
Me
3p
f1 (ppm)

1-(4-Methoxyphenyl)pent-4-en-1-one (3q)

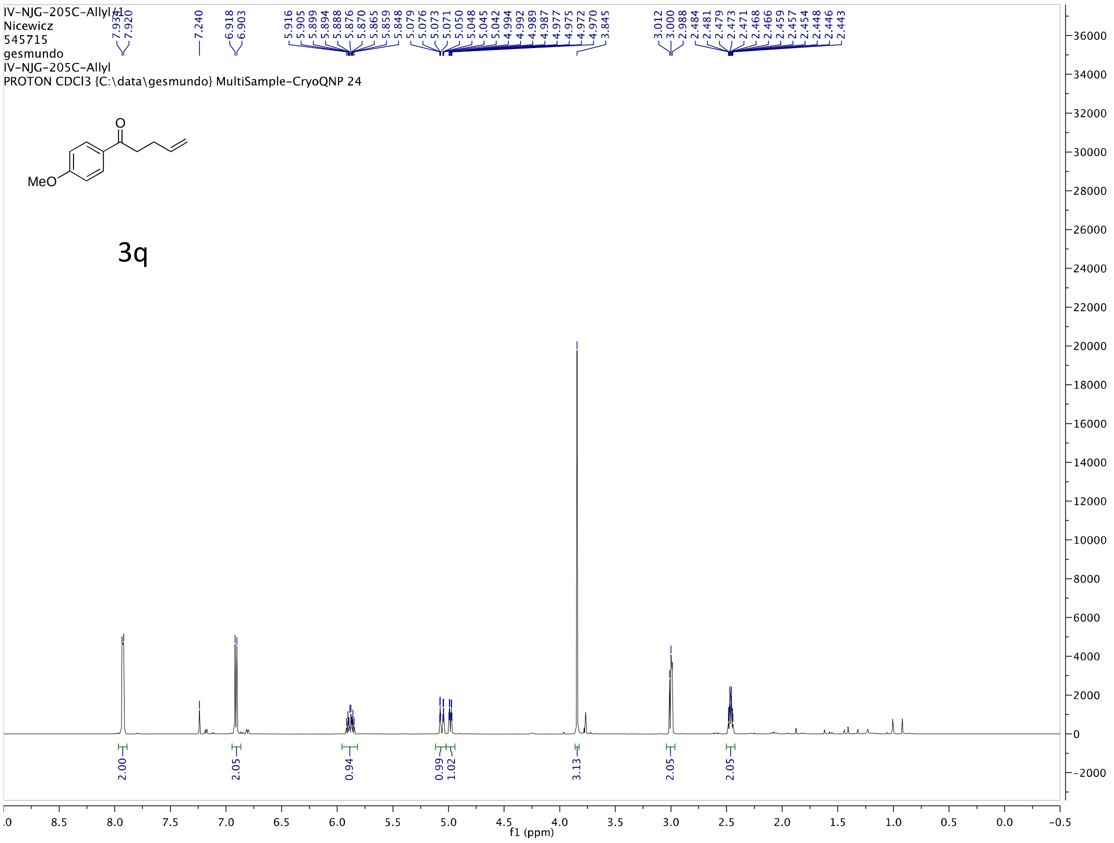

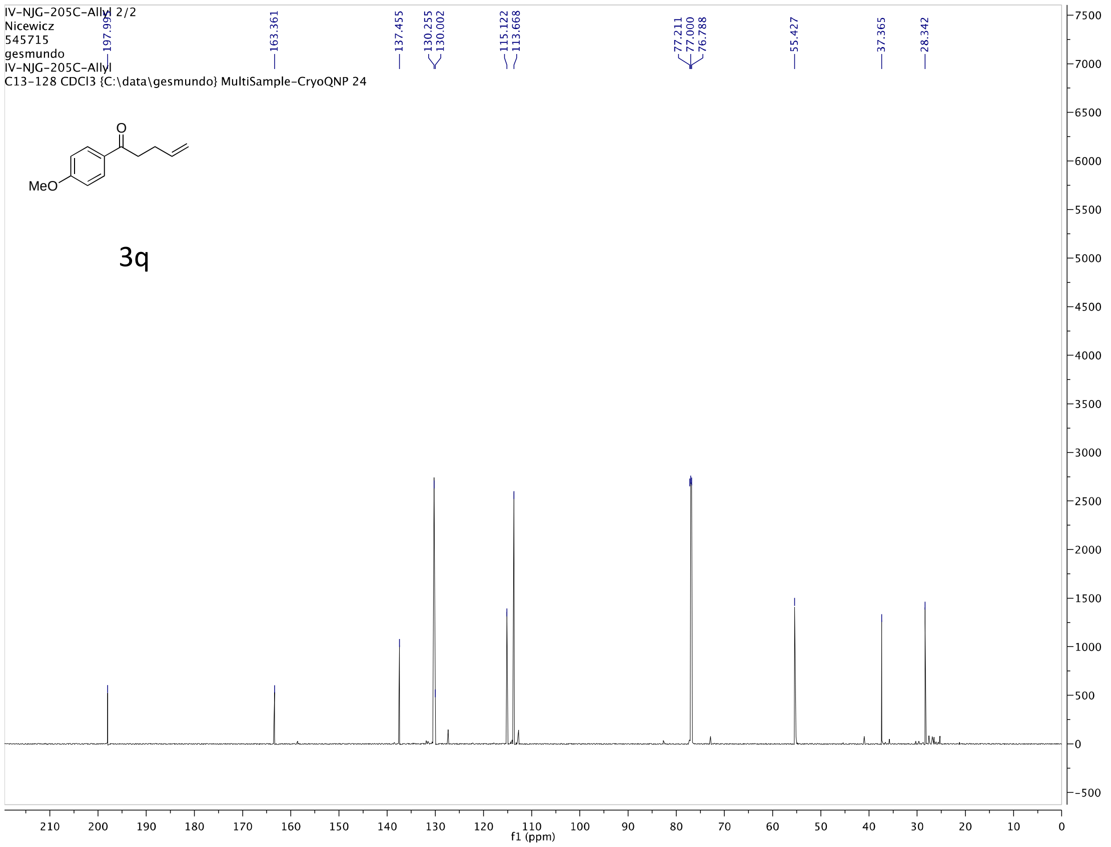
IV-NJG-205C-Allyl 2/2
Nicewicz
545715
gesmundo
IV-NJG-205C-Allyl
C13-128 CDCl3 {C:\data\gesmundo} MultiSample-CryoQNP 24
197.995
163.361
137.455
130.255
130.002
115.122
113.668
77.211
77.000
76.788
55.427
37.365
28.342
MeO
O
3q
f1 (ppm)

1-(4-Methoxyphenyl)-4,4-dimethyl-2,3,7-trioxabicyclo[3.3.1]nonane (3t)

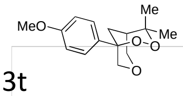

3t

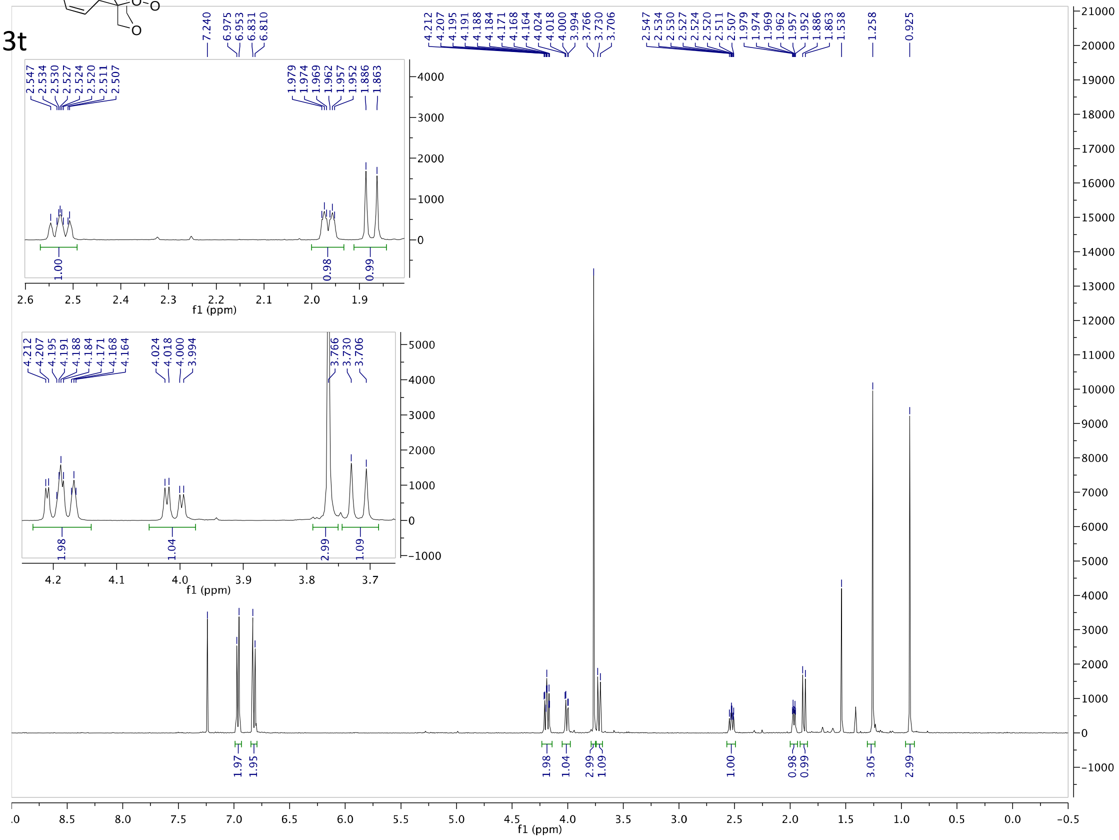

3t

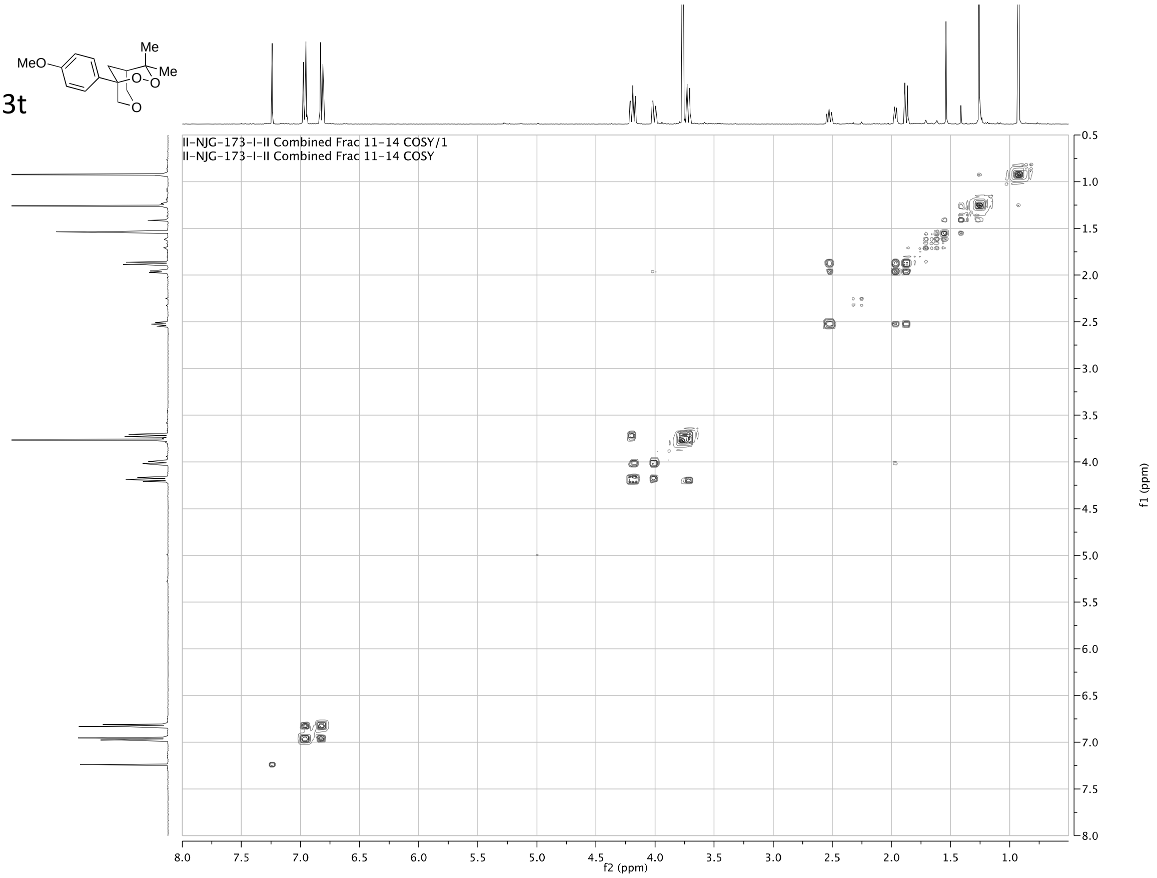
MeO
Me
Me
O
O
O
II-NJG-173-I-II Combined Frac 11-14 COSY/1
II-NJG-173-I-II Combined Frac 11-14 COSY
f2 (ppm)
f1 (ppm)

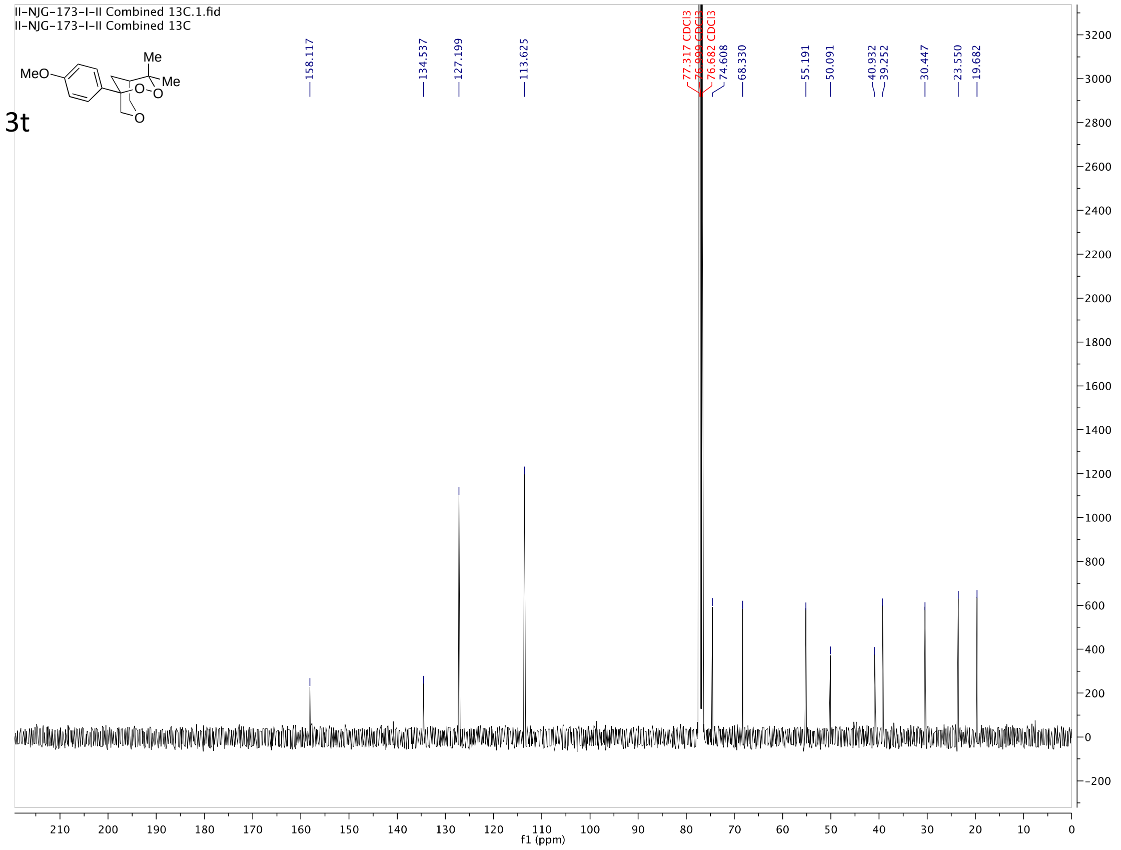
II-NJG-173-I-II Combined 13C.1.fid
II-NJG-173-I-II Combined 13C
MeO
Me
Me
O
O
O
3t
158.117
134.537
127.199
113.625
77.317 CDCl3
76.999 CDCl3
76.682 CDCl3
74.608
68.330
55.191
50.091
40.932
39.252
30.447
23.550
19.682
f1 (ppm)

1-(4-Methoxyphenyl)-4-phenyltetrahydro-1*H*,4*H*-furo[3,4-*d*][1,2]dioxine (3u)

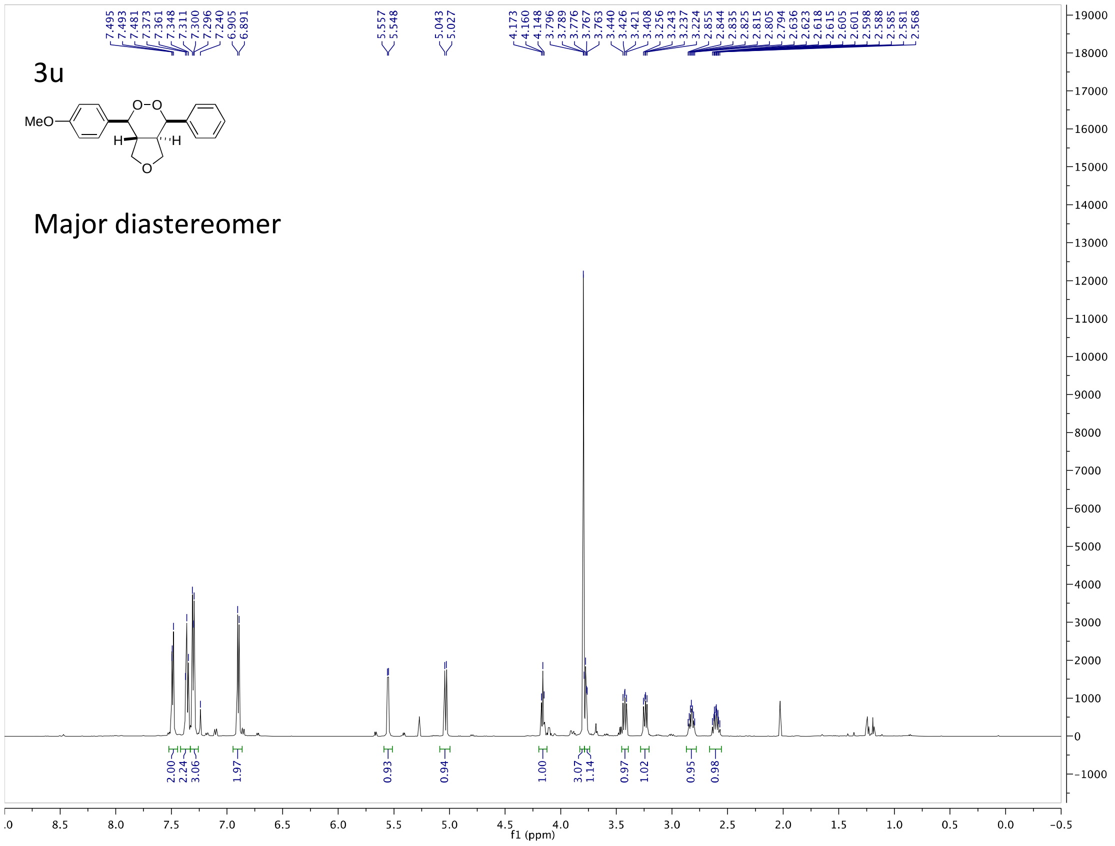

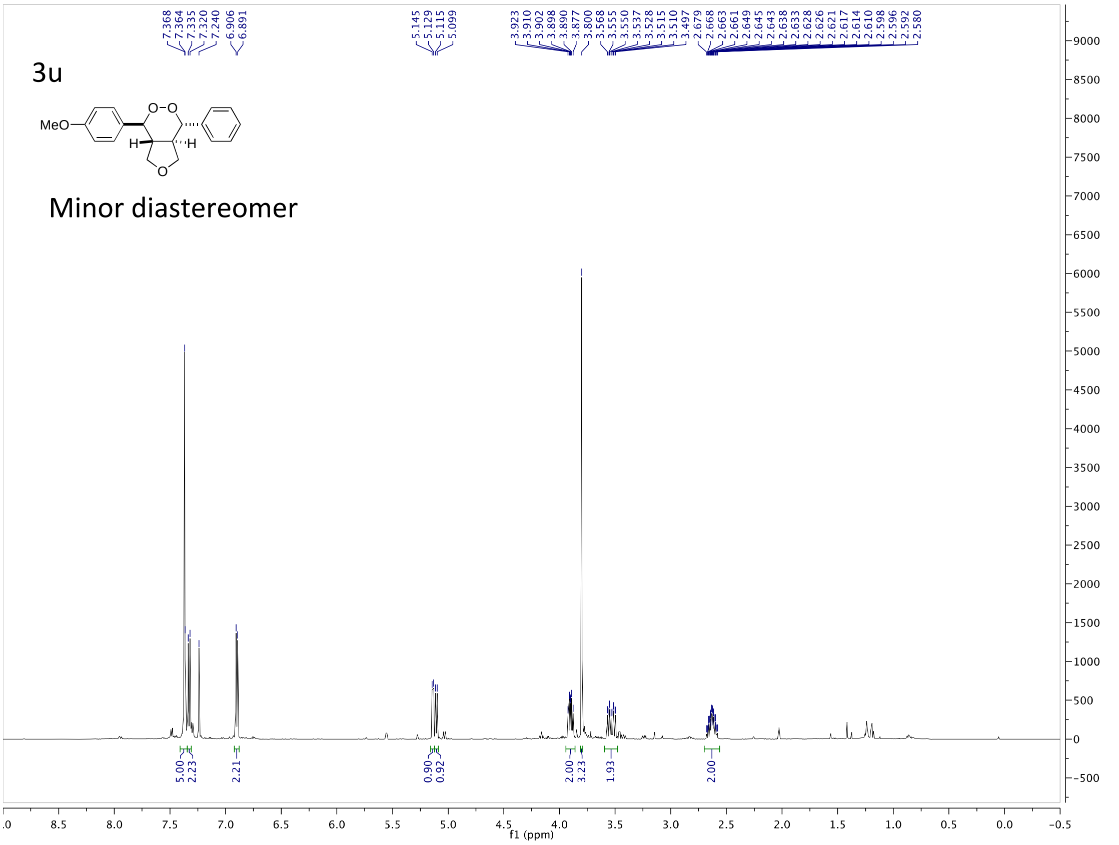
3u
MeO
O–O
H
H
O
Minor diastereomer
7.368
7.364
7.335
7.320
7.240
6.906
6.891
5.145
5.129
5.115
5.099
3.923
3.910
3.902
3.898
3.890
3.877
3.800
3.568
3.555
3.550
3.537
3.528
3.515
3.510
3.497
2.679
2.668
2.663
2.661
2.649
2.645
2.643
2.638
2.633
2.628
2.626
2.621
2.617
2.614
2.610
2.598
2.596
2.592
2.580
5.00
2.23
2.21
0.90
0.92
2.00
3.23
1.93
2.00
f1 (ppm)

(*E*)-1-(6-Hydroperoxy-3,3,6-trimethylhepta-1,4-dien-2-yl)-4-methoxybenzene

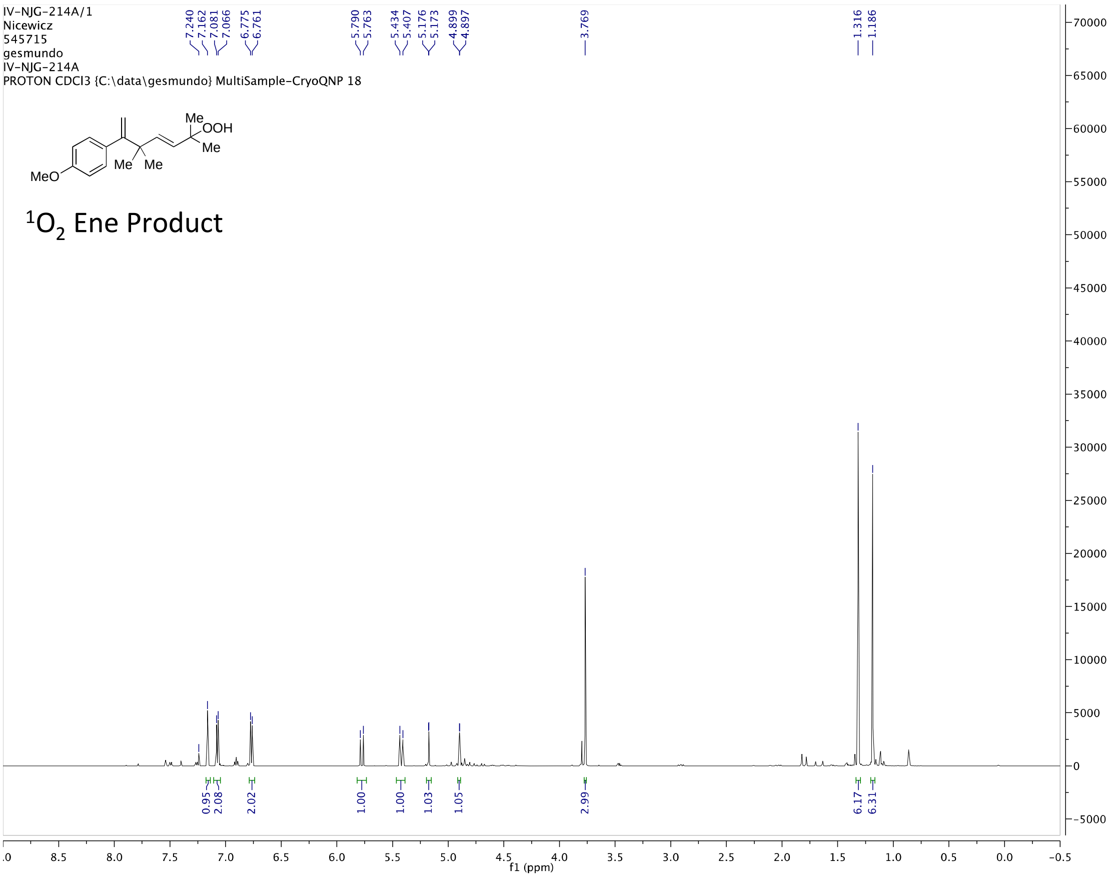

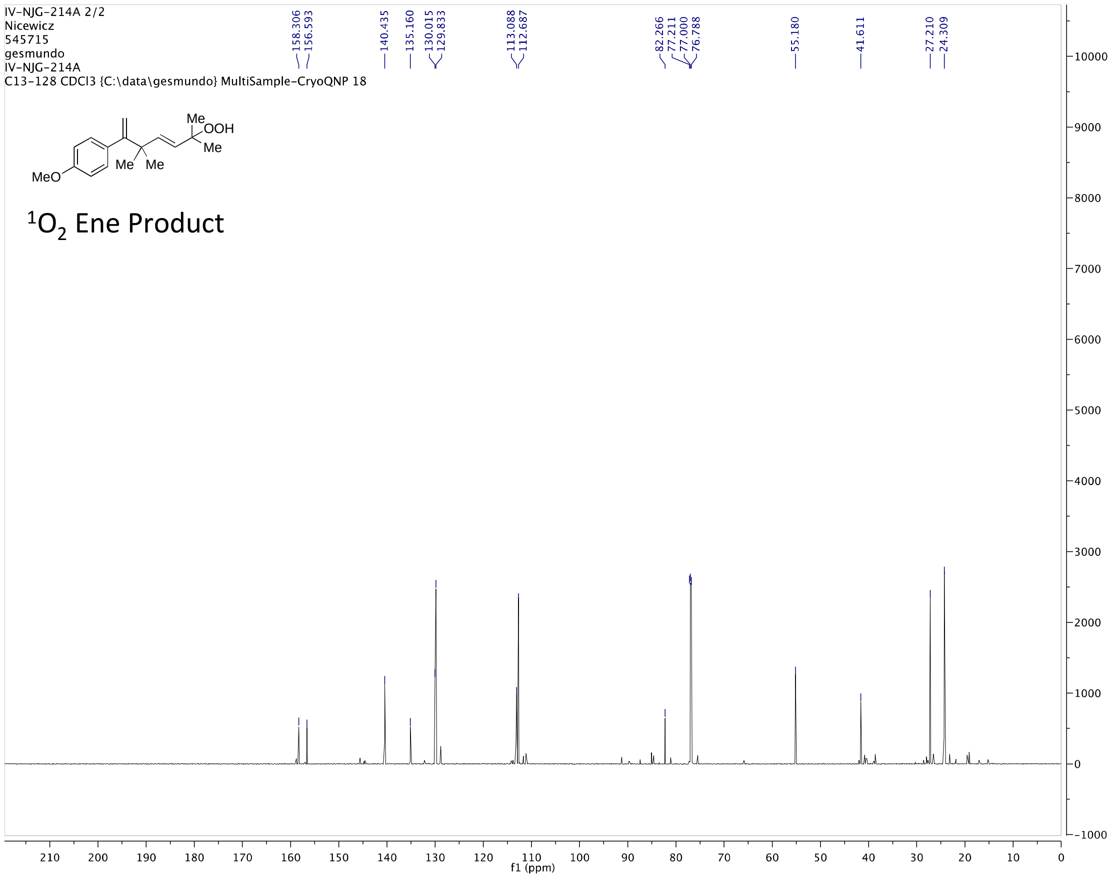

---

[1] Martiny, M.; Steckhan, E.; Esch, T. *Chem. Ber.* **1993**, *126*, 1671-1682.
[2] Gupton, J. T.; Layman, W. J. *J. Org. Chem.* **1987**, *52*, 3683-3686.
[3] Tokuyasu, T.; Kunikawa, S.; McCullough, K. J.; Masuyama, A.; Nojima, M. *J. Org. Chem.* **2005**, *70*, 251-260.
[4] Encina, M. V.; Lissi, E. A.; Lemp, E.; Zanocco, A.; Scaiano, J. C. *J. Am. Chem. Soc.* **1983**, *105*, 1856-1860.
[5] Crombie, B. S.; Smith, C.; Varnavas, C. Z.; Wallace, T. W. *J. Chem. Soc., Perkin Trans. 1*, **2001**, 206-215.
[6] Weyerstahl, P.; Marschall-Weyerstahl, H.; Scholz, S. *Liebigs Ann. Chem.* **1986**, *1986*, 1021–1029.
[7] Vyvan, J. R.; Loitz, C.; Looper, R. E.; Mattingly, C. S.; Peterson, E. A.; Staben, S. T. *J. Org. Chem.* **2004**, *69*, 2461-2468.
[8] Takemiya, A.; Hartwig, J. F. *J. Am. Chem. Soc.* **2006**, *128*, 6042-6043.
[9] Ikeda, H.; Minegishi, T.; Miyashi, T.; Lakkaraju, P. S.; Sauers, R. R.; Roth, H. D. *J. Phys. Chem. B*, **2005**, *109*, 2504-2511.
[10] Duan, Z.; Hu, X.; Zhang, C.; Wang, D.; Yu, S.; Zheng, Z. *J. Org. Chem.* **2009**, *74*, 9191-9194.

[11] Parrish, J. D.; Ischay, M. A.; Lu, Z.; Guo, S.; Peters, N. R.; Yoon, T. P. *Org. Lett.* **2012**, *14*, 1640-1643.

[12] Miyashi, T.; Konno, A.; Takahashi, Y. *J. Am. Chem. Soc.* **1988**, *110*, 3677-3679.

[13] Dörr, A. A.; Lubell, W. D. *Can. J. Chem.* **2007**, *85*, 1006-1017.
