# Supplementary material for: Cyclization–endoperoxidation cascade reactions of dienes mediated by a pyrylium photoredox catalyst
Source: Beilstein J Org Chem. 2014 Jun 3;10:1272–81. doi: 10.3762/bjoc.10.128 (PMC4077508; doi:10.3762/bjoc.10.128)
Supplement: File 2 — X-ray data. [file Beilstein_J_Org_Chem-10-1272-s002.pdf]

Supporting Information File 2

for

Cyclization–endoperoxidation cascade reactions of dienes mediated by a pyrylium photoredox catalyst

Nathan J. Gesmundo and David A. Nicewicz*

Address: Department of Chemistry, University of North Carolina at Chapel Hill, Chapel Hill, NC 27599-3290, USA

Email: David A. Nicewicz - nicewicz@unc.edu

*Corresponding author

X-ray data

3a

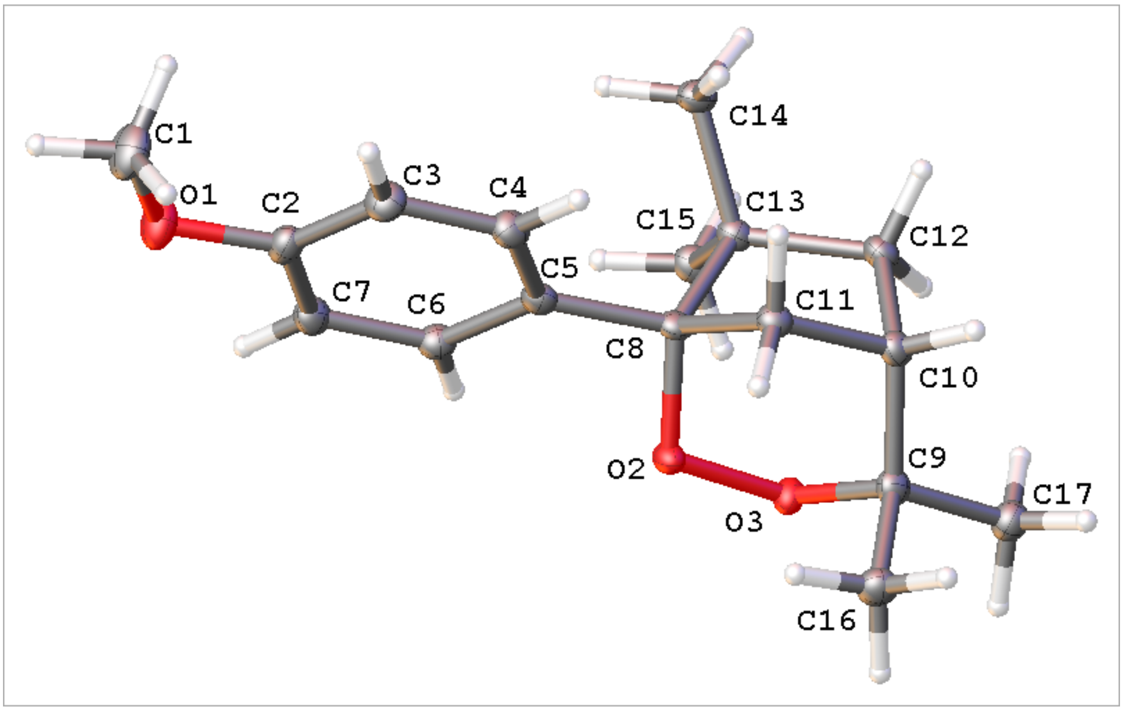

Table 1 Crystal data and structure refinement for x1402007.

Identification code	x1402007
Empirical formula	$C_{17}H_{24}O_3$
Formula weight	276.36
Temperature/K	100
Crystal system	monoclinic
Space group	$P2_1/c$
a/Å	11.4559(2)
b/Å	5.90270(10)
c/Å	22.2867(4)
α/°	90
β/°	102.5127(10)
γ/°	90
Volume/Å^3	1471.25(4)
Z	4
ρ_{calc}mg/mm^3	1.248
m/mm^{-1}	0.668
F(000)	600.0
Crystal size/mm^3	0.225 × 0.141 × 0.054
Radiation	CuKα (λ = 1.54178)
2Θ range for data collection	7.904 to 140.106°
Index ranges	$-13 \le h \le 13$, $-6 \le k \le 7$, $-27 \le l \le 27$
Reflections collected	15784

Independent reflections	2786 [R_{int} = 0.0285, R_{sigma} = 0.0191]
Data/restraints/parameters	2786/0/186
Goodness-of-fit on F^2	1.033
Final R indexes [I>=2σ (I)]	R_1 = 0.0351, wR_2 = 0.0852
Final R indexes [all data]	R_1 = 0.0407, wR_2 = 0.0891
Largest diff. peak/hole / e $Å^{-3}$	0.27/-0.20

Table 2 Fractional Atomic Coordinates (×10^4) and Equivalent Isotropic Displacement Parameters (Å^2×10^3) for x1402007. U_{eq} is defined as 1/3 of of the trace of the orthogonalised U_{IJ} tensor.

Atom	*x*	*y*	*z*	U(eq)
O1	3117.4(8)	8879.9(16)	6855.4(4)	23.6(2)
O2	7848.5(7)	5821.0(13)	6216.7(4)	15.08(19)
O3	8854.8(7)	5164.9(14)	5935.0(4)	15.6(2)
C1	2864.7(12)	10817(3)	7189.2(7)	28.3(3)
C2	4205.2(11)	8838(2)	6693.1(6)	17.8(3)
C3	5062.0(11)	10532(2)	6829.9(6)	19.4(3)
C4	6138.3(11)	10300(2)	6643.4(6)	18.3(3)
C5	6382.2(11)	8417(2)	6314.1(5)	15.1(3)
C6	5503.5(11)	6723(2)	6180.1(5)	16.5(3)
C7	4434.4(11)	6923(2)	6368.5(6)	17.9(3)
C8	7541.9(11)	8217.7(19)	6101.8(5)	13.8(3)
C9	9901.2(11)	6488(2)	6207.9(6)	16.2(3)
C10	9548.4(11)	8993(2)	6099.3(5)	15.6(3)
C11	8595.4(11)	9608(2)	6458.8(5)	15.3(3)
C12	8849.3(11)	9402(2)	5430.0(5)	16.4(3)
C13	7506.1(11)	8919(2)	5420.1(5)	14.9(3)
C14	6773.2(11)	11104(2)	5270.7(6)	18.0(3)
C15	6954.5(11)	7131(2)	4945.4(5)	17.6(3)
C16	10339.6(12)	5845(2)	6882.7(6)	20.4(3)
C17	10825.2(11)	5739(2)	5848.6(6)	20.0(3)

Table 3 Anisotropic Displacement Parameters ($Å^2×10^3$) for x1402007. The Anisotropic displacement factor exponent takes the form: -$2\pi^2[h^2a^{*2}U_{11}+2hka^*b^*U_{12}+…]$.

Atom	U_{11}	U_{22}	U_{33}	U_{23}	U_{13}	U_{12}
O1	17.9(5)	27.6(5)	27.2(5)	-8.3(4)	9.1(4)	-1.2(4)
O2	16.0(4)	11.9(4)	19.1(4)	1.6(3)	7.7(3)	1.3(3)
O3	13.1(4)	14.2(4)	20.4(4)	-2.0(3)	6.0(3)	0.0(3)
C1	22.0(7)	32.8(8)	32.4(8)	-10.6(6)	11.4(6)	1.2(6)
C2	15.2(6)	22.2(6)	16.3(6)	1.5(5)	3.8(5)	2.0(5)
C3	21.8(7)	17.5(6)	19.7(6)	-3.8(5)	6.0(5)	1.7(5)
C4	18.7(6)	15.6(6)	20.1(6)	-1.6(5)	3.2(5)	-2.2(5)
C5	17.0(6)	14.4(6)	13.6(6)	2.0(4)	2.4(5)	0.4(5)
C6	18.0(6)	14.4(6)	16.8(6)	-1.1(5)	3.2(5)	0.6(5)
C7	16.3(6)	17.4(6)	19.0(6)	-0.6(5)	1.6(5)	-2.7(5)
C8	16.2(6)	9.2(5)	16.0(6)	0.3(4)	3.2(5)	-0.2(4)
C9	14.4(6)	16.3(6)	17.4(6)	-0.3(5)	2.1(5)	-2.1(5)
C10	14.7(6)	15.1(6)	17.0(6)	0.5(4)	3.3(5)	-2.4(4)
C11	16.6(6)	12.6(6)	16.3(6)	-0.9(4)	3.0(5)	-1.0(4)
C12	18.3(6)	14.3(6)	17.1(6)	1.8(5)	5.0(5)	-1.5(5)
C13	16.7(6)	13.3(6)	14.5(6)	0.8(4)	3.2(5)	-0.7(4)
C14	19.2(6)	14.2(6)	19.7(6)	2.0(5)	2.3(5)	0.4(5)
C15	20.0(6)	16.8(6)	15.7(6)	-1.1(5)	2.8(5)	-1.1(5)
C16	20.1(6)	21.3(6)	19.1(6)	2.2(5)	2.5(5)	1.6(5)
C17	17.2(6)	20.8(6)	22.3(6)	-0.1(5)	4.8(5)	0.9(5)

Table 4 Bond Lengths for x1402007.

Atom	Atom	Length/Å	Atom	Atom	Length/Å
O1	C1	1.4274(16)	C6	C7	1.3832(17)
O1	C2	1.3710(15)	C8	C11	1.5320(16)
O2	O3	1.4788(11)	C8	C13	1.5666(16)
O2	C8	1.4668(13)	C9	C10	1.5381(17)
O3	C9	1.4497(14)	C9	C16	1.5267(17)
C2	C3	1.3883(18)	C9	C17	1.5246(17)
C2	C7	1.3969(17)	C10	C11	1.5311(17)
C3	C4	1.3906(18)	C10	C12	1.5515(16)
C4	C5	1.3930(17)	C12	C13	1.5604(16)
C5	C6	1.4046(17)	C13	C14	1.5356(16)
C5	C8	1.5083(17)	C13	C15	1.5310(16)

Table 5 Bond Angles for x1402007.

Atom	Atom	Atom	Angle/°	Atom	Atom	Atom	Angle/°
C2	O1	C1	116.79(10)	C11	C8	C13	102.36(9)
C8	O2	O3	110.96(8)	O3	C9	C10	106.67(9)
C9	O3	O2	109.30(8)	O3	C9	C16	110.18(10)
O1	C2	C3	124.66(11)	O3	C9	C17	103.21(9)
O1	C2	C7	115.79(11)	C16	C9	C10	114.40(10)
C3	C2	C7	119.55(12)	C17	C9	C10	112.64(10)
C2	C3	C4	119.60(11)	C17	C9	C16	109.12(10)
C3	C4	C5	121.88(12)	C9	C10	C12	111.35(10)
C4	C5	C6	117.63(12)	C11	C10	C9	109.77(10)
C4	C5	C8	121.23(11)	C11	C10	C12	100.85(9)
C6	C5	C8	121.13(11)	C10	C11	C8	99.92(9)
C7	C6	C5	121.03(11)	C10	C12	C13	106.69(9)
C6	C7	C2	120.31(11)	C12	C13	C8	102.48(9)
O2	C8	C5	102.39(9)	C14	C13	C8	109.31(10)
O2	C8	C11	107.10(9)	C14	C13	C12	109.93(10)
O2	C8	C13	112.29(9)	C15	C13	C8	114.08(10)
C5	C8	C11	116.33(10)	C15	C13	C12	113.19(10)
C5	C8	C13	116.35(10)	C15	C13	C14	107.76(10)

Table 6 Torsion Angles for x1402007.

A	B	C	D	Angle/°	A	B	C	D	Angle/°
O1	C2	C3	C4	179.72(11)	C5	C8	C11	C10	-178.65(9)
O1	C2	C7	C6	179.60(11)	C5	C8	C13	C12	159.27(10)
O2	O3	C9	C10	-57.77(11)	C5	C8	C13	C14	42.69(13)
O2	O3	C9	C16	66.95(11)	C5	C8	C13	C15	-78.00(13)
O2	O3	C9	C17	-176.65(8)	C6	C5	C8	O2	-41.40(13)
O2	C8	C11	C10	67.60(11)	C6	C5	C8	C11	-157.80(11)
O2	C8	C13	C12	-83.23(11)	C6	C5	C8	C13	81.43(13)
O2	C8	C13	C14	160.19(9)	C7	C2	C3	C4	-0.17(18)
O2	C8	C13	C15	39.49(14)	C8	O2	O3	C9	61.55(10)
O3	O2	C8	C5	169.76(8)	C8	C5	C6	C7	-179.16(11)
O3	O2	C8	C11	-67.37(11)	C9	C10	C11	C8	-68.43(11)
O3	O2	C8	C13	44.23(11)	C9	C10	C12	C13	86.59(12)
O3	C9	C10	C11	65.88(12)	C10	C12	C13	C8	-0.80(11)
O3	C9	C10	C12	-44.93(13)	C10	C12	C13	C14	115.34(10)
C1	O1	C2	C3	-0.08(18)	C10	C12	C13	C15	-124.12(11)
C1	O1	C2	C7	179.81(11)	C11	C8	C13	C12	31.30(11)
C2	C3	C4	C5	0.81(19)	C11	C8	C13	C14	-85.28(11)
C3	C2	C7	C6	-0.50(18)	C11	C8	C13	C15	154.02(10)
C3	C4	C5	C6	-0.76(18)	C11	C10	C12	C13	-29.82(11)
C3	C4	C5	C8	178.48(11)	C12	C10	C11	C8	49.14(10)
C4	C5	C6	C7	0.08(17)	C13	C8	C11	C10	-50.67(10)
C4	C5	C8	O2	139.39(11)	C16	C9	C10	C11	-56.20(13)
C4	C5	C8	C11	22.99(16)	C16	C9	C10	C12	-167.01(10)
C4	C5	C8	C13	-97.79(13)	C17	C9	C10	C11	178.42(10)
C5	C6	C7	C2	0.54(18)	C17	C9	C10	C12	67.60(13)

Table 7 Hydrogen Atom Coordinates (Å×10^4) and Isotropic Displacement Parameters (Å^2×10^3) for x1402007.

Atom	*x*	*y*	*z*	U(eq)
H1A	2860	12177	6937	42
H1B	3480	10969	7568	42
H1C	2081	10634	7292	42
H3	4914	11842	7049	23
H4	6724	11458	6744	22
H6	5646	5419	5957	20
H7	3853	5754	6277	21
H10	10261	10011	6209	19
H11A	8419	11251	6439	18
H11B	8830	9127	6894	18
H12A	9141	8373	5144	20
H12B	8955	10985	5305	20
H14A	5933	10794	5272	27
H14B	6836	11656	4864	27
H14C	7082	12257	5581	27
H15A	7444	5754	5010	26
H15B	6923	7717	4531	26
H15C	6143	6775	4992	26
H16A	9696	6088	7102	31
H16B	11029	6788	7066	31
H16C	10576	4246	6913	31
H17A	10938	4096	5888	30
H17B	11587	6504	6013	30
H17C	10545	6136	5414	30

Experimental

Single crystals of $C_{17}H_{24}O_3$ **[x1402007]** were **[]**. A suitable crystal was selected and **[]** on a **'Bruker APEX-II CCD'** diffractometer. The crystal was kept at 100 K during data collection. Using Olex2 [1], the structure was solved with the olex2.solve [2] structure solution program using Charge Flipping and refined with the ShelXL [3] refinement package using Least Squares minimisation.

1. Dolomanov, O.V., Bourhis, L.J., Gildea, R.J, Howard, J.A.K. & Puschmann, H. (2009), J. Appl. Cryst. 42, 339-341.
2. Bourhis, L.J., Dolomanov, O.V., Gildea, R.J., Howard, J.A.K., Puschmann, H. (2013). in preparation
3. Sheldrick, G.M. (2008). Acta Cryst. A64, 112-122

Crystal structure determination of [x1402007]

Crystal Data for $C_{17}H_{24}O_3$ (M =276.36): monoclinic, space group P2$_1$/c (no. 14), a = 11.4559(2) Å, b = 5.90270(10) Å, c = 22.2867(4) Å, β = 102.5127(10)°, V = 1471.25(4) Å^3, Z = 4, T = 100 K, μ(CuKα) = 0.668 mm^{-1}, $Dcalc$ = 1.248 g/mm^3, 15784 reflections measured (7.904 ≤ 2Θ ≤ 140.106), 2786 unique (R_{int} = 0.0285, R_{sigma} = 0.0191) which were used in all calculations. The final R_1 was 0.0351 (I > 2σ(I)) and wR_2 was 0.0891 (all data).

Refinement model description

Number of restraints - 0, number of constraints - unknown.

Details:

```
1. Fixed Uiso
 At 1.2 times of:
  All C(H) groups, All C(H,H) groups
 At 1.5 times of:
  All C(H,H,H) groups
2.a Ternary CH refined with riding coordinates:
 C10(H10)
2.b Secondary CH2 refined with riding coordinates:
 C11(H11A,H11B), C12(H12A,H12B)
2.c Aromatic/amide H refined with riding coordinates:
 C3(H3), C4(H4), C6(H6), C7(H7)
2.d Idealised Me refined as rotating group:
 C1(H1A,H1B,H1C), C14(H14A,H14B,H14C), C15(H15A,H15B,H15C), C16(H16A,H16B,
 H16C), C17(H17A,H17B,H17C)
```

This report has been created with Olex2, compiled on 2013.12.10 svn.r2850 for OlexSys. Please let us know if there are any errors or if you would like to have additional features.
